# Electrophotocatalytic Undirected C−H Trifluoromethylations of (Het)Arenes

**DOI:** 10.1002/chem.201905774

**Published:** 2020-02-19

**Authors:** Youai Qiu, Alexej Scheremetjew, Lars H. Finger, Lutz Ackermann

**Affiliations:** ^1^ Institut für Organische und Biomolekulare Chemie Georg-August-Universität Göttingen Tammannstrasse 2 37077 Göttingen Germany

**Keywords:** arenes, catalysis, C−H trifluoromethylation, electrophotochemistry, oxidant-free

## Abstract

Electrophotochemistry has enabled arene C−H trifluoromethylation with the Langlois reagent CF_3_SO_2_Na under mild reaction conditions. The merger of electrosynthesis and photoredox catalysis provided a chemical oxidant‐free approach for the generation of the CF_3_ radical. The electrophotochemistry was carried out in an operationally simple manner, setting the stage for challenging C−H trifluoromethylations of unactivated arenes and heteroarenes. The robust nature of the electrophotochemical manifold was reflected by a wide scope, including electron‐rich and electron‐deficient benzenes, as well as naturally occurring heteroarenes. Electrophotochemical C−H trifluoromethylation was further achieved in flow with a modular electro‐flow‐cell equipped with an in‐operando monitoring unit for on‐line flow‐NMR spectroscopy, providing support for the single electron transfer processes.

## Graphical abstract

**Cooperation**: Electrophotochemical C−H trifluoromethylations of unbiased arenes were accomplished by the cooperative action of electricity and light under mild reaction conditions.

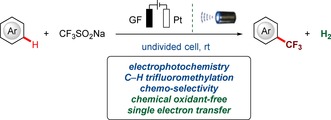

In the past decade, electrosynthesis has been identified as an increasingly powerful tool for replacing stoichiometric chemical redox reagents.^1^ Based on recent contributions by Baran,^2^ Waldvogel,^3^ Ackermann,^4^ Yoshida,^5^ and Xu,^6^ among others,^7^ this strategy has recently gained momentum by alkene functionalizations,^8^ directed C−H oxygenations,^9^ as well as C−H activations^10^ by transition metal catalysis.^11^ Meanwhile, visible light photoredox catalysis has emerged as a transformative technique for molecular syntheses,^12^ proving particularly effective for single electron transfer cross‐coupling‐type reactions. Despite these indisputable advances in the respective areas, the merger of electrochemistry with photochemistry continues to be underdeveloped.

Trifluoromethylated compounds display unique bioactivity and lipophilicity, and are hence in particularly high demand in medicinal chemistry and pharmaceutical industries.^13^ Very recently, aromatic trifluoromethylations have made the transition from more traditional processes^14^ to photoredox catalysis^15^ and electro‐synthesis.^16^ In the meantime, we became intrigued by the prospect of combining^17^ electrochemistry and photochemistry for C−H trifluoromethylations of non‐activated arenes under mild reaction conditions. In this context, Moutet and Reverdy17i reported the electrophotocatalytic oxidation of benzylic alcohol, albeit with limited scope. Further, Scheffold17h demonstrated the nucleophilic acylation of Michael olefins using vitamin B12 as an electrophotocatalyst. Very recently, elegant examples of electrophotochemical transformations were achieved by Xu,17d Stahl,17f Hu,17c and Lambert.17a, 17b within our program on sustainable C−H activation^18^ induced by photocatalysis,^19^ we have now developed the first electrophotochemical C−H trifluoromethylation through single electron transfer (SET) with the inexpensive, solid, and stable Langlois reagent (CF_3_SO_2_Na)^20^ (Figure 1). Hence, our strategy set the stage for the merger of sustainable electrochemical and photochemical transformations towards trifluoromethylation. Notable features of our approach include (a) unprecedented electrophotochemical C−H trifluoromethylations with electricity as the sustainable sole oxidant, (b) electrophotochemical C−H activation in a flow setup, and (c) in‐operando flow‐NMR monitoring.

**Figure 1:**
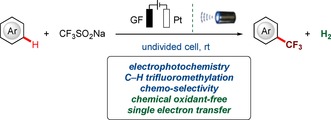
Electrophotochemical undirected C−H trifluoromethylation.

Our studies were initiated by exploring the electrophotochemical nondirected C−H trifluoromethylation of readily accessible mesitylene (**1 a**)^21^ with the Langlois reagent (CF_3_SO_2_Na, **2**) in an operationally‐simple, user‐friendly undivided cell set‐up. These orienting studies utilized a platinum plate cathode and a graphite felt (GF) anode, employing [Mes‐Acr^+^] ClO_4_^−^ as a photocatalyst (PC) and cost‐effective KOAc as a conductive additive in CH_3_CN at a room temperature (rt) of 23 °C (Scheme 1).

**Scheme 1:**
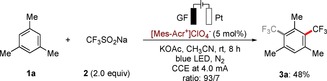
Preliminary electrophotochemical C−H trifluoromethylation.

With these preliminary results in hand, we further interrogated the electrophotochemical C−H trifluoromethylation regime (Table 1). After considerable experimentation, we were pleased to observe that the desired product **3 a** was best obtained with LiClO_4_ as additive, and CH_3_CN as the solvent in an operationally simple undivided cell (entries 1–6). Notably, further catalyst optimization showed that [Ru(bpy)_3_](PF_6_)_2_ efficiently furnished the corresponding product **3 a** at lower catalyst loading (entry 7). Control experiments verified the indispensable role of electricity and light, while confirming the key importance of the photocatalyst (entries 9–13). The use of further additives, such as water or TFA, fell short in improving the efficiency of the desired transformation (entries 14–15), whereas a less expensive nickel foam cathode gave the corresponding products with comparable levels of efficiency under otherwise identical reaction conditions (entry 16). Thus, two optimized conditions were identified featuring a metal‐free photocatalyst (conditions A: [Mes‐Acr^+^]ClO_4_^−^) or alternatively a ruthenium photocatalyst (conditions B: [Ru(bpy)_3_](PF_6_)_2_).

**Table 1:** Optimization of the electrophotochemical C−H trifluoromethylation.^[a]^

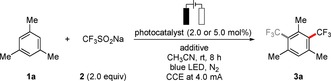					
Entry	Photocatalyst	Additive	Solvent	Yield [%]^[b]^	Ratio [%]^[b]^
1	[Mes‐Acr^+^]ClO_4_^−^	KOAc	CH_3_CN	48	93:7
2	[Mes‐Acr^+^]ClO_4_^−^	TBAPF_6_	CH_3_CN	10	–
**3**	**[Mes‐Acr^+^]ClO_4_** ^−^	**LiClO_4_**	**CH_3_CN**	**85**	**93:7**
4	[Mes‐Acr^+^]ClO_4_^−^	LiClO_4_	DCE	38	94:6
5	[Mes‐Acr^+^]ClO_4_^−^	LiClO_4_	TFE	45	80:20
6	[Mes‐Acr^+^]ClO_4_^−^	LiClO_4_	HFIP	68	62:38
**7**	**[Ru(bpy)_3_](PF_6_)_2_**	**LiClO_4_**	**CH_3_CN**	**88**	**78:22**
8	Eosin Y	LiClO_4_	CH_3_CN	75	83:17
9	[Mes‐Acr^+^]ClO_4_^−^	LiClO_4_	CH_3_CN	5	–^[c]^
10	[Mes‐Acr^+^]ClO_4_^−^	LiClO_4_	CH_3_CN	4	–^[d]^
11	[Mes‐Acr^+^]ClO_4_^−^	LiClO_4_	CH_3_CN	8	–^[e]^
12	–	LiClO_4_	CH_3_CN	9	–
13	[Mes‐Acr^+^]ClO_4_^−^	–	CH_3_CN	55	92:8
14	[Mes‐Acr^+^]ClO_4_^−^	LiClO_4_	CH_3_CN	70	92:8^[f]^
15	[Mes‐Acr^+^]ClO_4_^−^	LiClO_4_	CH_3_CN	23	–^[g]^
16	[Mes‐Acr^+^]ClO_4_^−^	LiClO_4_	CH_3_CN	52	87:13^[h]^

[a] Undivided cell, graphite felt anode, Pt cathode, constant current=4.0 mA, **1** (0.25 mmol), **2** (0.50 mmol), photocatalyst (2.0 or 5.0 mol %), additive (0.1 m), solvent (4.0 mL), 23 °C, blue LED, under N_2_, 8 h. [b] Yields determined by ^1^H NMR with CH_2_Br_2_ as internal standard, and ratio is mono‐/bis‐ CF_3_ substituents. [c] Without electricity under N_2_ in degassed solvent. [d] Without electricity under air. [e] Without blue light. [f] Additive: H_2_O (2.0 equiv). [g] Additive: TFA (2.0 equiv). [h] Nickel foam as cathode. Standard conditions A: [Mes‐Acr^+^]ClO_4_^−^ (5.0 mol %) as catalyst (Faradaic yield: 36 %); standard conditions B: [Ru(bpy)_3_](PF_6_)_2_ (2.0 mol %) as catalyst (Faradaic yield: 37 %).

With the thus optimized reaction conditions for the electrophotochemical C−H trifluoromethylation in hand, we explored their versatility with a set of representative arenes **1** (Scheme 2). We were delighted to observe that substrates **1** bearing either electron‐donating or electron‐withdrawing substituents underwent the C−H transformations efficiently with high regioselectivity to afford the corresponding products **3 a**–**3 g** and **3 h**, respectively. Even sensitive electrophilic functional groups, such as ester and chloro substituents, or sterically‐encumbered substituents (**3 c**), were fully tolerated. The synergistic electrophotochemistry was hence characterized by high levels of chemo‐selectivity control. Some inert examples were also exploited for this transformation.^22^

**Scheme 2:**
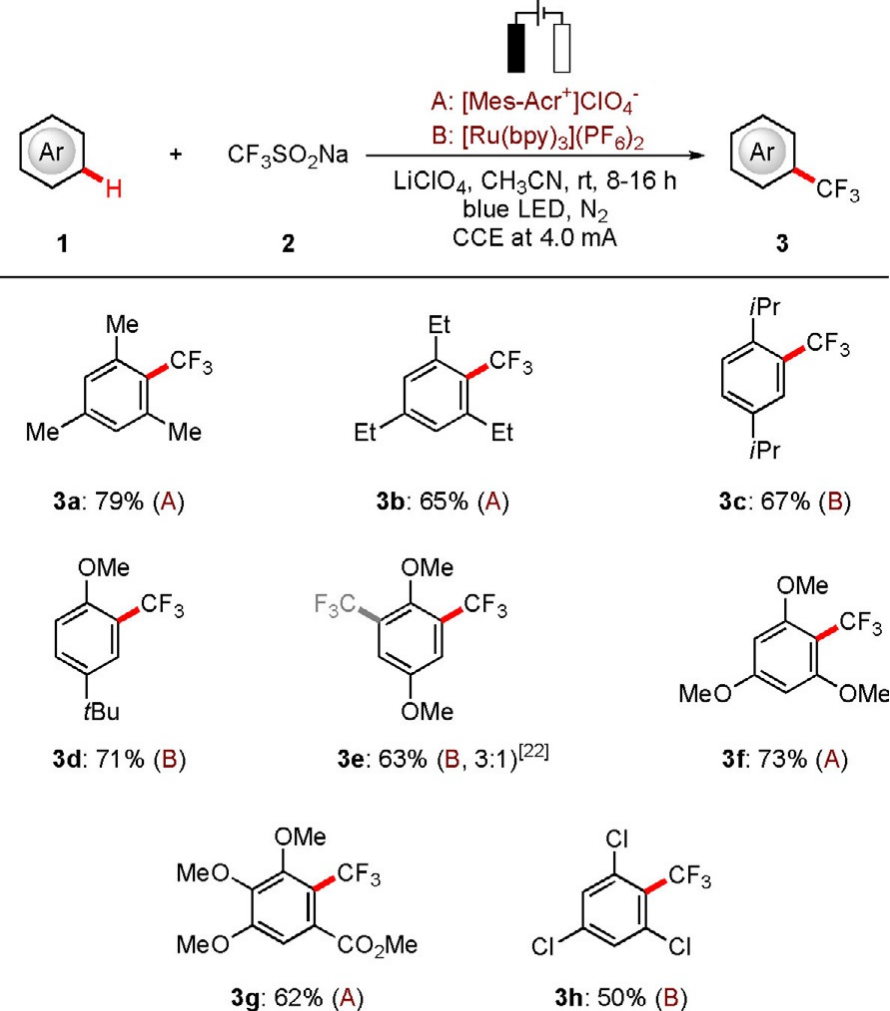
Electrophotochemical C−H trifluoromethylation of arenes **1**.

Thereafter, we turned our attention towards probing the scope of viable heteroarenes **4** (Scheme 3). A wide range of heteroarenes **4** proved to be compatible with the optimized electrophotochemical transformation, including furan, thiophene, benzofuran, benzo[*b*]thiophene, indole, quinoline, and pyrimidine, furnishing the desired trifluoromethylated products **5** with high selectivity and good efficiency. Notably, synthetically useful ester, amide, and acetyl substituents were well tolerated, which should prove valuable for further late‐stage modifications.

**Scheme 3:**
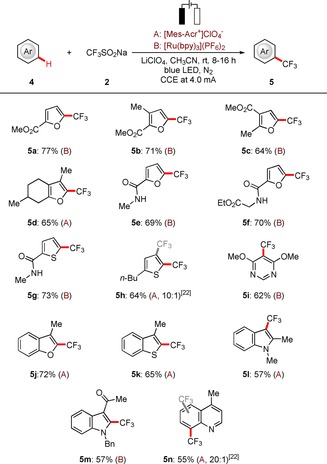
Electrophotochemical C−H trifluoromethylation of heteroarenes **4**.

The power of direct C−H functionalizations is arguably best represented by late‐stage diversification of structurally complex molecules.^23^ Remarkably, the unique robustness of the synergistic electrophotochemical C−H trifluoromethylation allowed for the efficient conversion of naturally occurring motifs, such as caffeine, pentoxifylline, doxofylline, theobromine, methyl estrone, and tryptophan derivatives, thereby highlighting the utility of the electrophotochemical C−H transformation strategy for late‐stage trifluoromethylations (Scheme 4).

**Scheme 4:**
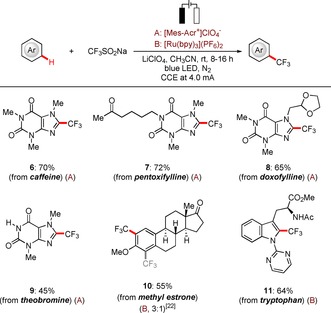
Electrophotochemical late‐stage diversification of bioactive and natural products.

Based on the results of the electrophotochemical C−H trifluoromethylation in batch, we became intrigued by probing the C−H trifluoromethylation in flow.^24^ To our delight, a modular electro‐flow‐cell equipped with a GF anode and a nickel cathode followed by a looped transparent tube delivered under irradiation the desired product **3 a** with high efficiency, when using the PC **A** (Scheme 5).^25^ Notably, these findings show that the electro‐oxidation and the photo‐catalysis steps can occur with spatial and temporal separation, for the organic photocatalyst.

**Scheme 5:**
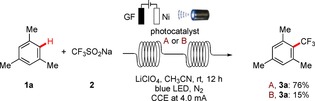
Electrophotochemical C−H trifluoromethylation in a flow setup, flow rate: 1.0 mL min^−1^, residence time in the electrochemical flow reactor: 6 min.

Considering the remarkable performance of the electrophotochemical C−H activation, we became intrigued to delineate its mode of action. To this end, we first conducted typical radical trap experiments (Scheme 6). Hence, the desired product **3 a** was not formed in the presence of 2.0 equivalents of TEMPO (2,2,6,6‐tetramethyl‐1‐piperidinyloxide). Further, the C−H trifluoromethylation was significantly suppressed upon the addition of stoichiometric (3,5‐di‐*tert*‐butyl‐4‐hydroxytoluene) (BHT), the observations being suggestive of a single‐electron‐transfer process. In the latter case, the products **12** and **13** were obtained instead, providing further support for a SET manifold.

**Scheme 6:**
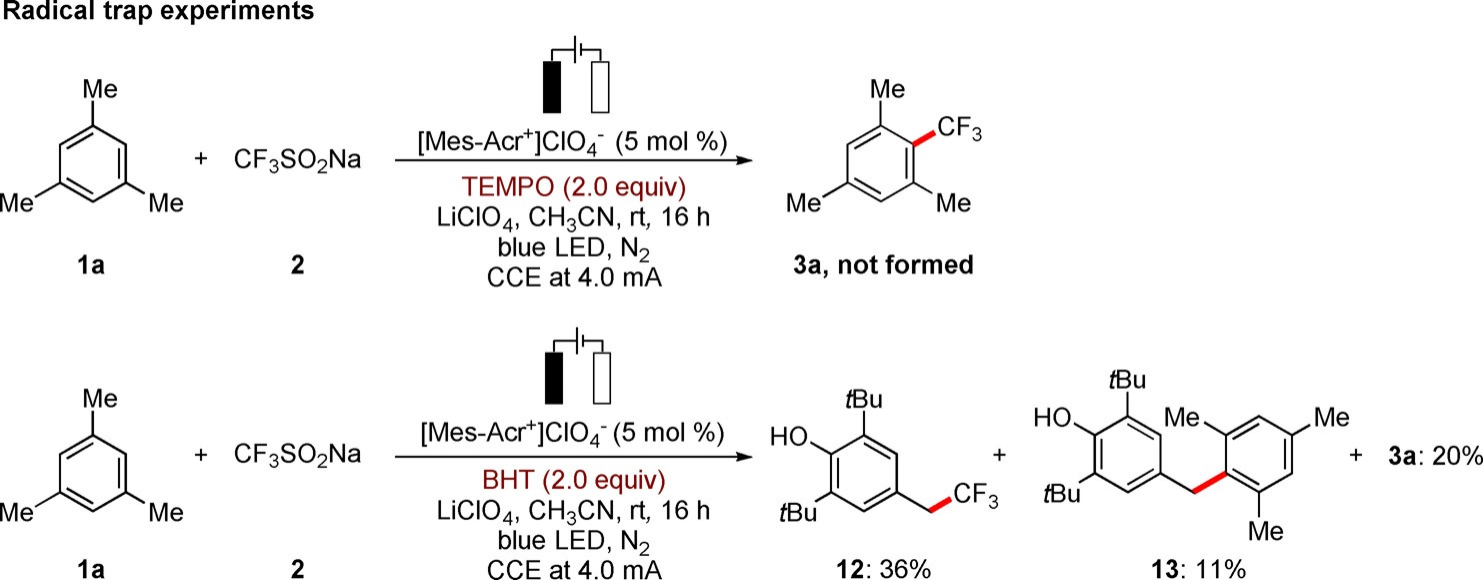
Mechanistic studies.

Moreover, we performed fluorescence quenching experiments towards a Stern–Volmer plot analyses (see the Supporting Information), and monitored the conversion profile of the electrophotochemical C−H trifluoromethylation. Thus, a Stern–Volmer plot analysis provided strong support for an effective quenching of the photo‐excited MesAcr* PC, occurring preferentially by the reagent **2** (Figure 2 a).^22^ These studies revealed the transformations being suppressed in the absence of light (Figure S7), thus demonstrating the key influence of continuous irradiation. We further conducted electricity on/off experiments (Figure S8), and the transformations were fully suppressed in the absence of electricity. These findings highlight the importance of visible‐light irradiation and electricity for the success of the C−H trifluoromethylation.^26^

**Figure 2:**
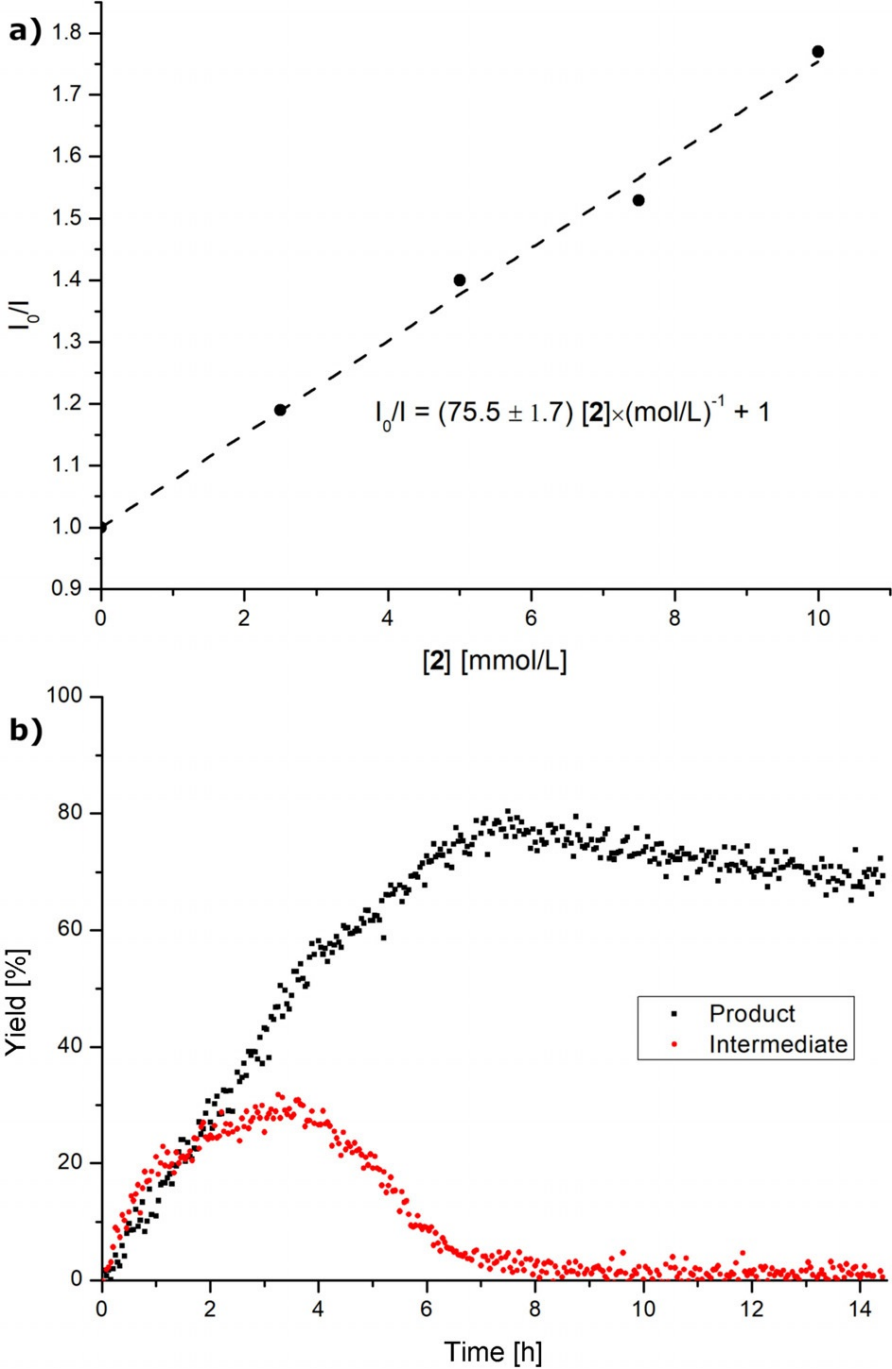
a) Stern–Volmer plot analysis: Fluorescence quenching of [Mes‐Acr^+^]ClO_4_^−^ with reagent **2**. b) On‐line reaction monitoring in flow by ^19^F NMR spectroscopy.

To rationalize the reaction profile in more detail we additionally monitored the reaction progress by ^1^H and ^19^F NMR spectroscopy in flow. The ^19^F NMR monitoring flow‐experiment depicted in Figure 2 b features the readily formed reaction product **3 a** and remarkably the emergence and decomposition of a long‐lived intermediate. To our delight, the intermediate could also be observed during ^1^H NMR monitoring (see the Supporting Information) indicating the intermediate to possess two equivalent protons with a resonance at 5.80 ppm. Thereby, the intermediate is tentatively assigned as **Int‐B**—a Wheland type arenium cation,^27^ for which related resonances have been observed.27a

At the same time, we conducted cyclic voltammetry (CV) studies in acetonitrile with LiClO_4_ (0.1 m) as the electrolyte (Figure 3). The onset of the irreversible sodium trifluoromethanesulfinate **2** oxidation was observed at E_onset_=+1.02 V vs. SCE. In this process, a species is generated, which exhibits a reductive current that initiates at E_onset_=−0.57 V vs. SCE. We attribute this event to the formation of SO_2_.^20^ In the presence of the photocatalyst (PC) this behavior remained unaltered. However, upon blue light irradiation, a consumption of sodium trifluoromethanesulfinate **2** was observed. These findings suggest the formation of the CF_3_ radical, which occurs by oxidation with the excited state of the PC (E_red_=+2.06 V vs. SCE in MeCN).^28^ The PC can be regenerated in the ground state by anodic oxidation, which was indicated by the reversible redox event at E_1/2_=−0.62 V vs. SCE. At the counter electrode, molecular hydrogen is generated through cathodic reduction, as was evidenced by headspace gas chromatographic analysis.^22^

**Figure 3:**
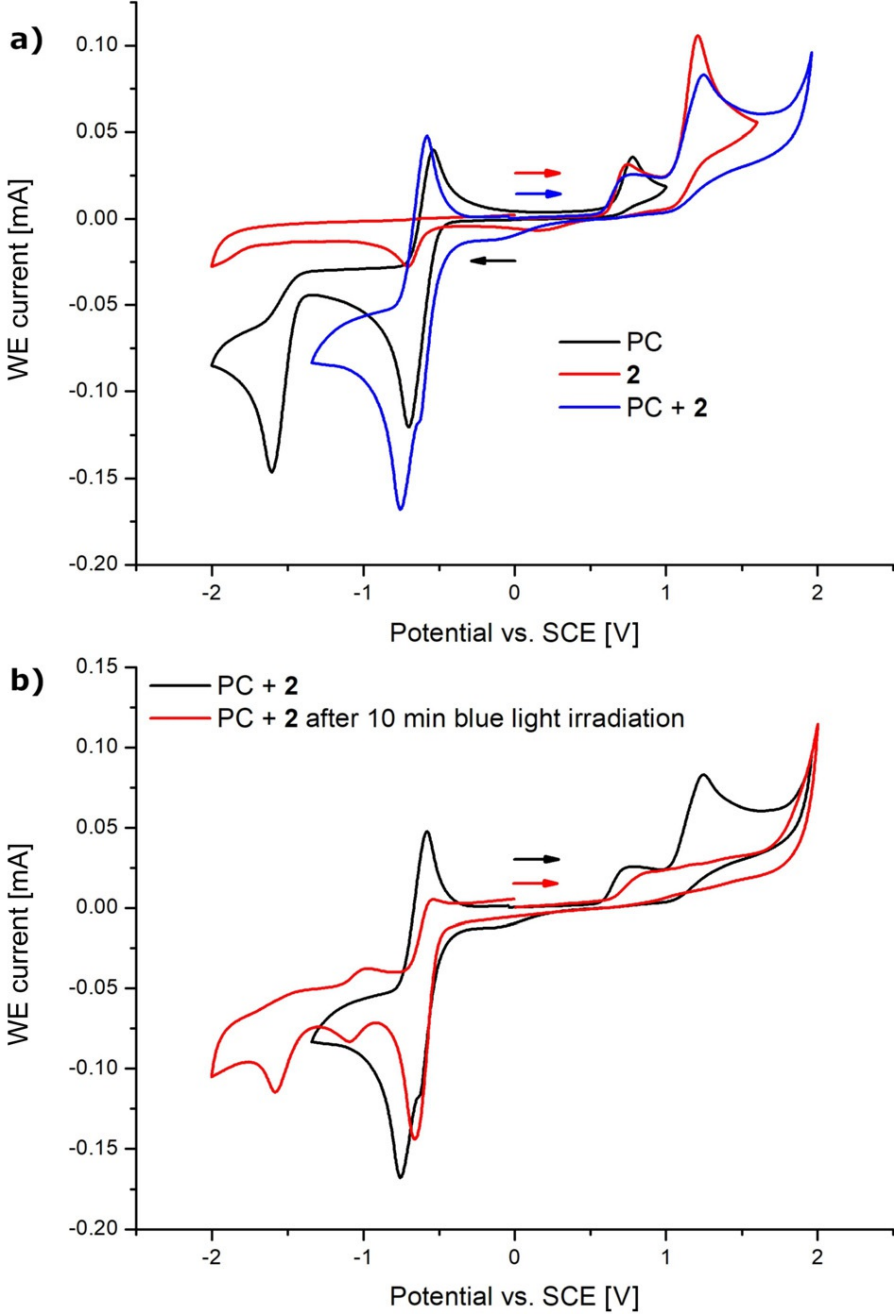
Cyclic voltammetry. Conditions: (a) and (b) substrates (5 mmol L^−1^), LiClO_4_ (100 mmol L^−1^), MeCN, 100 mV s^−1^. (a) PC (black), **2** (red), PC+**2** (blue). (b) PC+**2** (black), PC+**2** after being irradiated for 10 minutes with blue light (red).

On the basis of our mechanistic findings, we propose a plausible mechanism as depicted in Scheme 7.^25^ Initial irradiation of the organic dye Mes‐Acr^+^ generates its oxidized excited state Mes‐Acr^+^*. A SET process between Mes‐Acr^+^* and the sulfinate anion results in the generation of the acridinyl radical and the CF_3_SO_2_ radical. Anodic electrooxidation of the acridinyl radical subsequently regenerates the ground state catalyst Mes‐Acr^+^. Simultaneously, attack of the trifluoromethyl radical on the substrate **1 a** forms the **Int‐A** radical, which undergoes SET oxidation to form the cationic Wheland complex **Int‐B**. In the meantime, **Int‐A** could also be directly oxidized at the anode. Finally, proton abstraction delivers the desired product **3 a**, whereas protons are reduced to generate H_2_ at the cathode.

**Scheme 7:**
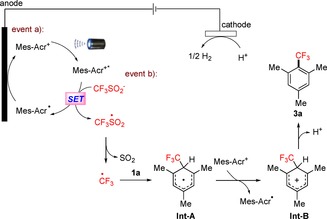
Plausible mechanism.

In conclusion, we have developed the first electrophotochemical C−H trifluoromethylation enabled by the cooperative action of electrochemistry and photochemistry. This strategy avoids the use of expensive and toxic chemical oxidants. Under the electrophotochemical conditions, trifluoromethyl radicals are efficiently generated, which readily participate in intermolecular oxidative C−H transformations. The reaction features a broad substrate scope and a high tolerance of synthetically useful functional groups. The electrophotochemical C−H trifluoromethylations were further achieved in a flow setup. The practical utility of the electrophotochemical C−H trifluoromethylation in flow was reflected by operationally simple on‐line NMR‐monitoring. Mechanistic studies provided strong support for key SET processes, overall indicating the synthetic potential of electrophotochemical transformations.

## Conflict of interest

The authors declare no conflict of interest.

## Supporting information

As a service to our authors and readers, this journal provides supporting information supplied by the authors. Such materials are peer reviewed and may be re‐organized for online delivery, but are not copy‐edited or typeset. Technical support issues arising from supporting information (other than missing files) should be addressed to the authors.

Supplementary
