# Supplementary material for: Electrophotocatalytic Undirected C−H Trifluoromethylations of (Het)Arenes
Source: Chemistry. 2020 Feb 19;26(15):3241–6. doi: 10.1002/chem.201905774 (PMC7155051; doi:10.1002/chem.201905774)
Supplement: Supplementary file 1 — Supplementary [file CHEM-26-3241-s001.pdf]

CHEMISTRY
A European Journal

Supporting Information

Electrophotocatalytic Undirected C—H Trifluoromethylations of (Het)Arenes

Youai Qiu, Alexej Scheremetjew, Lars H. Finger, and Lutz Ackermann*[a]

chem_201905774_sm_miscellaneous_information.pdf

Table of Contents

General Remarks S-2

Optimization of the Electrophotochemical C–H Trifluoromethylation S-3

General Procedure for the Electrophotochemical C–H Trifluoromethylation S-5

Characterization Data of Products S-6

General Procedure for the Electrophotochemical C–H Trifluoromethylation in Flow S-21

On-Line NMR Monitoring in Flow S-23

Radical Trap Experiments S-26

Light on/off Experiments S-29

Electricity on/off Experiments S-30

Estimation of Quantum Yield S-31

Fluorescence Quenching Experiments S-33

GC-Headspace Detection S-36

Cyclic Voltammetry S-37

Plausible Mechanism with $[Ru(bpy)_3](PF_6)_2$ as Catalyst S-40

References S-41

NMR-Spectra S-42

General Remarks

Catalytic reactions were carried out in undivided electrochemical cells (10 mL) using pre-dried glassware, if not noted otherwise. Substrates, CF_3SO_2Na and solvents were obtained from commercial sources. Platinum electrodes (10 mm × 15 mm × 0.25 mm, 99.9%; obtained from ChemPur® Karlsruhe, Germany) and graphite felt electrodes (10 mm × 15 mm × 6 mm, SIGRACELL® GFA 6 EA, obtained from SGL Carbon, Wiesbaden, Germany) were connected using stainless steel adapters. Electrocatalysis was conducted using an AXIOMET AX-3003P potentiostat in constant current mode. For reactions in flow an Ismatec REGLO Digital MS-2/12 (ISM 596) peristaltic pump was employed. The ^{19}F and ^{1}H NMR spectroscopy experiments in flow were performed on a Magritek Spinsolve 60^{ULTRA} (from Magritek GmbH, Germany). Cyclic Voltammetry studies were performed using a Metrohm Autolab PGSTAT204 workstation and Nova 2.1 software. Yields refer to isolated compounds, estimated to be >95% pure as determined by ^{1}H-NMR. Chromatography was carried out on Merck silica gel 60 (40–63 µm). NMR spectra were recorded on a Varian Mercury VX 300, Inova 500 or Bruker Avance III 300, Avance III 400 and Avance III HD 500 in the solvent indicated; chemical shifts (δ) are given in ppm relative to the residual solvent peak. All IR spectra were recorded on a Bruker FT-IR Alpha-P device. EI-MS was recorded on Jeol AccuTOF at 70eV, ESI-MS on Bruker MicrOTOF and maXis. GC-MS was recorded on Agilent 7890B and Agilent 5977B. M. p.: Stuart melting point apparatus SMP3, Barloworld Scientific, values are uncorrected. Headspace analysis of the reaction mixture was performed on a Shimadzu S2014 GC System using a Thermal Conductivity Detector and a 5Å MS column.

Optimization of the Electrophotochemical C–H Trifluoromethylation

Table S-1: Optimization of the electrophotochemical C–H trifluoromethylation.[a]

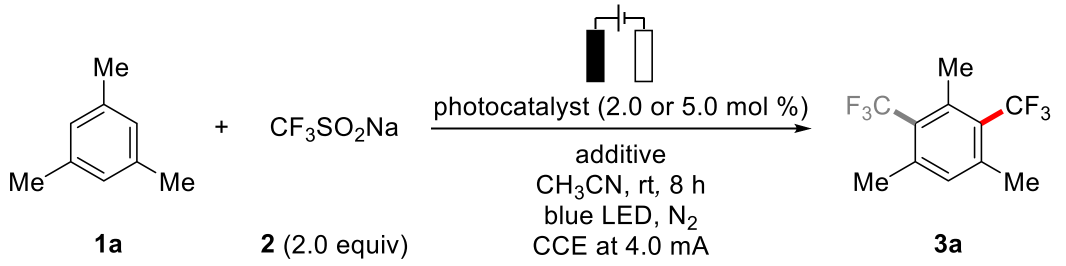

Entry	Photocatalyst	Additive	Solvent	Yield (%)[b]	Ratio (%)[b]
1	$[Mes\text{-}Acr^+]ClO_4^-$	KOAc	CH_3CN	48	93/7
2	$[Mes\text{-}Acr^+]ClO_4^-$	$TBAPF_6$	CH_3CN	10	-
3	$[Mes\text{-}Acr^+]ClO_4^-$	$LiClO_4$	CH_3CN	85	93/7
4	$[Mes\text{-}Acr^+]ClO_4^-$	$LiClO_4$	DCE	38	94/6
5	$[Mes\text{-}Acr^+]ClO_4^-$	$LiClO_4$	TFE	45	80/20
6	$[Mes\text{-}Acr^+]ClO_4^-$	$LiClO_4$	HFIP	68	62/38
7	$[Ru(bpy)_3](PF_6)_2$	$LiClO_4$	CH_3CN	88	78/22
8	Eosin Y	$LiClO_4$	CH_3CN	75	83/17
9[c]	$[Mes\text{-}Acr^+]ClO_4^-$	$LiClO_4$	CH_3CN	5	-
10[d]	$[Mes\text{-}Acr^+]ClO_4^-$	$LiClO_4$	CH_3CN	8	-
11[c]	$[Ru(bpy)_3](PF_6)_2$	$LiClO_4$	CH_3CN	trace	-
12[d]	$[Ru(bpy)_3](PF_6)_2$	$LiClO_4$	CH_3CN	7	-
13[e]	$[Mes\text{-}Acr^+]ClO_4^-$	$LiClO_4$	CH_3CN	4	-
14[e]	$[Ru(bpy)_3](PF_6)_2$	$LiClO_4$	CH_3CN	3	-
15	-	$LiClO_4$	CH_3CN	9	-
16	$[Mes\text{-}Acr^+]ClO_4^-$	-	CH_3CN	55	92/8
17[f]	$[Mes\text{-}Acr^+]ClO_4^-$	$LiClO_4$	CH_3CN	70	92/8
18[g]	$[Mes\text{-}Acr^+]ClO_4^-$	$LiClO_4$	CH_3CN	23	
19	$[Mes\text{-}Acr^+]ClO_4^-$	-	CH_3CN	55	92/8
10	$[Ru(bpy)_3](PF_6)_2$	-	CH_3CN	60	80/20
21	$[Ru(bpy)_3]Cl_2{\cdot}6H_2O$	-	CH_3CN	10	-
22[h]	$[Ru(bpy)_3](PF_6)_2$	$LiClO_4$	CH_3CN	75	86/14
23[i]	$[Mes\text{-}Acr^+]ClO_4^-$	$LiClO_4$	CH_3CN	52	87/13

[a] Undivided cell, graphite felt (GF) anode, Pt cathode, constant current = 4.0 mA, **1** (0.25 mmol), **2** (0.50 mmol), photocatalyst (2.0 or 5.0 mol %), additive (0.1 M), solvent (4.0 mL), 23 °C, blue LED, under N_2, 8 h. [b] Yields determined by ^1H NMR with CH_2Br_2 as internal standard, and ratio is mono-/bis- CF_3 substituents. [c] Without electricity under N_2 after degassing. [d] Without blue light. [e] Without electricity in air. [f]Additive: H_2O (2.0 equiv). [g] Additive: TFA (2.0 equiv). [h] **2** (1.5 equiv). [i] Nickel foam as cathode. Standard condition A: $[Mes\text{-}Acr^+]ClO_4^-$ (5.0 mol %) as catalyst (Faradaic yield: 36%); standard condition B: $[Ru(bpy)_3](PF_6)_2$ (2.0 mol %) as catalyst (Faradaic yield: 37%).

Figure S-1. Set-up of experiments

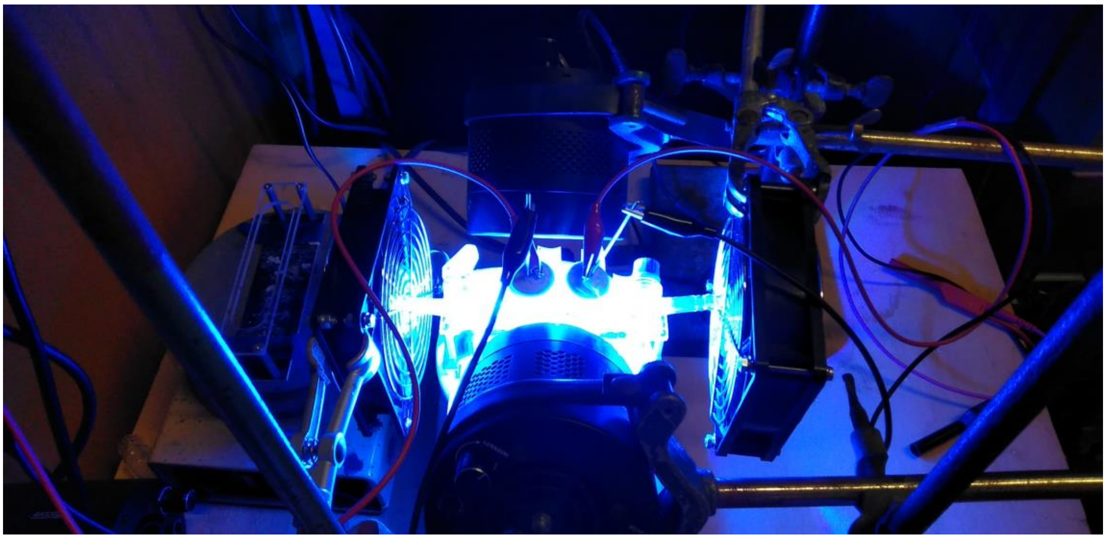

General Procedure A for the Electrophotochemical C–H Trifluoromethylation

The electrophotocatalysis was carried out in an undivided cell with a GF anode (10 mm × 15 mm × 6 mm) and a Pt cathode (10 mm × 15 mm × 0.25 mm). Substrate **1** or **4** (0.25 mmol, 1.0 equiv), CF_3SO_2Na **2** (78 mg, 0.50 mmol, 2.0 equiv), $LiClO_4$ (42 mg, 0.40 mmol) and [Mes-Acr$^+$]ClO_4^- (5.1 mg, 5.0 mol %) were dissolved in CH_3CN (4.0 mL) under N_2. The electrophotocatalysis was performed at 23 °C with a constant current of 4.0 mA maintained for 8-16 h under visible light irradiation (2 × Kessil A360N lamp). The GF anode was washed with CH_2Cl_2 (3 × 10 mL) in an ultrasonic bath. Evaporation of the solvent and subsequent column chromatography on silica gel afforded the corresponding products.

General Procedure B for the Electrophotochemical C–H Trifluoromethylation

The electrophotocatalysis was carried out in an undivided cell with a GF anode (10 mm × 15 mm × 6 mm) and a Pt cathode (10 mm × 15 mm × 0.25 mm). Substrate **1** or **4** (0.25 mmol, 1.0 equiv), CF_3SO_2Na **2** (78 mg, 0.50 mmol, 2.0 equiv), $LiClO_4$ (42 mg, 0.40 mmol) and $[Ru(bpy)_3](PF_6)_2$ (4.3 mg, 2.0 mol %) were dissolved in CH_3CN (4.0 mL) under N_2. The electrophotocatalysis was performed at 23 °C with a constant current of 4.0 mA maintained for 8-16 h under visible light irradiation (2 × Kessil A360N). The GF anode was washed with CH_2Cl_2 (3 × 10 mL) in an ultrasonic bath. Evaporation of the solvent and subsequent column chromatography on silica gel afforded the corresponding products.

Characterization Data of Products.

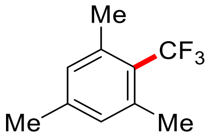

3a

1,3,5-Trimethyl-2-(trifluoromethyl)benzene (3a)

The general procedure A was followed using **1a** (30 mg, 0.25 mmol) at 23 °C for 8 h. Purification by column chromatography on silica gel (pentane) yielded **3a** (37 mg, 79%) as a colorless oil. ^{1}H-NMR (400 MHz, $CDCl_3$): δ = 6.88 (s, 2H), 2.44–2.40 (m, 6H), 2.27 (s, 3H). ^{13}C-NMR (100 MHz, $CDCl_3$): δ = 140.8 (C_q), 137.3 (q, $^{3}J_{C\text{–}F}$ = 2.2 Hz, C_q), 130.8 (CH), 126.2 (q, $^{1}J_{C\text{–}F}$ = 275.8 Hz, C_q), 124.8 (q, $^{2}J_{C\text{–}F}$ = 28.0 Hz, C_q), 21.31 (q, $^{4}J_{C\text{–}F}$ = 4.1 Hz, CH_3), 20.9 (CH_3). ^{19}F-NMR (376 MHz, $CDCl_3$): δ = −53.7 (s). IR (ATR): 2925, 2854, 1459, 1379, 1294, 1152, 1115 cm^{-1}. MS (EI) *m/z* (relative intensity): 188 (100) $[M]^+$. HR-MS (EI) *m/z* calc. for $C_{10}H_{11}F_3$ $[M]^+$: 188.0807, found: 188.0815. The analytical data correspond with those reported in the literature.[1]

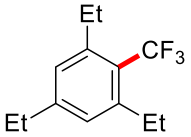

3b

1,3,5-Triethyl-2-(trifluoromethyl)benzene (3b)

The general procedure A was followed using **1b** (41 mg, 0.25 mmol) at 23 °C for 16 h. Purification by column chromatography on silica gel (pentane) yielded **3b** (38 mg, 65%) as a colorless oil. ^{1}H-NMR (300 MHz, $CDCl_3$): δ = 6.99 (s, 2H), 2.88–2.76 (m, 4H), 2.65 (q, J = 7.6 Hz, 2H), 1.27 (t, J = 7.6 Hz, 3H), 1.26 (t, J = 7.4 Hz, 6H). ^{13}C-NMR (100 MHz, $CDCl_3$): δ = 147.3 (C_q), 143.9 (q, $^{3}J_{C\text{–}F}$ = 1.9 Hz, C_q), 128.5 (CH), 126.2 (q, $^{1}J_{C\text{–}F}$ = 276.2 Hz, C_q), 123.7 (q, $^{2}J_{C\text{–}F}$ = 28.4 Hz, C_q), 28.4 (CH_2), 27.9 (q, $^{4}J_{C\text{–}F}$ = 3.8 Hz, CH_2), 16.6 (q, $^{5}J_{C\text{–}F}$ = 1.6 Hz, CH_3), 15.1 (CH_3). ^{19}F-NMR (282 MHz, $CDCl_3$): δ = −52.3 (s). IR (ATR): 2968, 2936, 2880, 1609, 1575, 1458, 1294, 1143, 1106, 1037 cm^{-1}. MS (EI) *m/z* (relative intensity): 230 (30) $[M]^+$, 215 (100) $[M\text{-}CH_3]^+$. HR-MS (EI) *m/z* calc. for $C_{13}H_{17}F_3$ $[M]^+$: 230.1277, found: 230.1279.

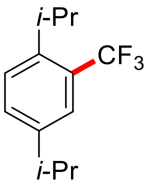

3c

1,4-Diisopropyl-2-(trifluoromethyl)benzene (3c)

The general procedure B was followed using **1c** (41 mg, 0.25 mmol) at 23 °C for 16 h. Purification by column chromatography on silica gel (pentane) yielded **3c** (39 mg, 67%) as a colorless oil. ^{1}H-NMR (400 MHz, $CDCl_3$): δ = 7.42–7.39 (m, 1H), 7.37 (d, J = 8.2 Hz, 1H), 7.35–7.31 (m, 1H), 3.37–3.23 (m, 1H), 2.90 (hept, J = 7.0 Hz, 1H), 1.23 (d, J = 7.0 Hz, 6H), 1.23 (d, J = 6.8 Hz, 6H). ^{13}C-NMR (100 MHz, $CDCl_3$): δ = 146.2 (C_q), 145.3 (q, $^{3}J_{C\text{–}F}$ = 1.4 Hz, C_q), 130.0 (CH), 127.2 (CH), 127.1 (q, $^{2}J_{C\text{–}F}$ = 28.4 Hz, C_q), 124.8 (q, $^{1}J_{C\text{–}F}$ = 272.3 Hz, C_q), 123.5 (q, $^{3}J_{C\text{–}F}$ = 5.9 Hz, CH), 33.6 (CH), 28.9 (q, $^{4}J_{C\text{–}F}$ = 1.8 Hz, CH), 24.3 (CH_3), 23.8 (CH_3). ^{19}F-NMR (376 MHz, $CDCl_3$): δ = −58.9 (s). IR (ATR): 2960, 2926, 1462, 1315, 1148, 1122, 1054 cm^{-1}. MS (EI) *m/z* (relative intensity): 230 (30) $[M]^+$, 215 (100) $[M\text{-}CH_3]^+$. HR-MS (EI) *m/z* calc. for $C_{13}H_{17}F_3$ $[M]^+$: 230.1277, found: 230.1282.

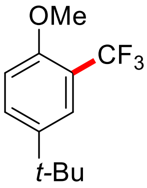

3d

4-(*tert*-Butyl)-1-methoxy-2-(trifluoromethyl)benzene (3d)

The general procedure B was followed using **1d** (41 mg, 0.25 mmol) at 23 °C for 8 h. Purification by column chromatography on silica gel (*n*-hexane) yielded **3d** (41 mg, 71%) as a colorless oil. ^{1}H-NMR (500 MHz, $CDCl_3$): δ = 7.57–7.52 (m, 1H), 7.51–7.44 (m, 1H), 6.92 (d, J = 8.6 Hz, 1H), 3.87 (s, 3H), 1.29 (s, 9H). ^{13}C-NMR (125 MHz, $CDCl_3$): δ = 155.2 (q, $^{3}J_{C\text{–}F}$ = 1.6 Hz, C_q), 142.9 (C_q), 129.9 (CH), 123.9 (q, $^{3}J_{C\text{–}F}$ = 5.3 Hz, CH), 123.9 (q, $^{1}J_{C\text{–}F}$ = 272.4 Hz, C_q), 118.0 (q, $^{2}J_{C\text{–}F}$ = 30.2 Hz, C_q), 111.7 (CH) , 56.0 (CH_3), 34.2 (C_q), 31.3 (CH_3). ^{19}F-NMR (470 MHz, $CDCl_3$): δ = −62.1 (s). IR (ATR): 2963, 2910, 1620, 1509, 1325, 1281, 1254, 1127, 1058, 1028 cm^{-1}. MS (EI) *m/z* (relative intensity): 232 (30) $[M]^+$, 217 (100) $[M\text{-}CH_3]^+$. HR-MS (EI) *m/z* calc. for $C_{12}H_{15}F_3O$ $[M]^+$: 232.1070, found: 232.1069. The analytical data correspond with those reported in the literature.[1]

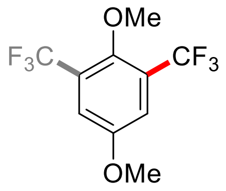

3e (mono/bis = 3/1)

1,4-Dimethoxy-2-(trifluoromethyl)benzene (3e)

The mono/bis ratio was determined to be 3:1 by ^{1}H-NMR analysis of the crude reaction mixture. The general procedure B was followed using **1e** (35 mg, 0.25 mmol) at 23 °C for 16 h. Purification by column chromatography on silica gel (*n*-hexane/EtOAc = 50:1) yielded **3e** (33 mg, 63%) as a colorless oil. ^{1}H-NMR (300 MHz, $CDCl_3$): δ = 7.17–7.11 (m, 1H), 7.08–7.01 (m, 1H), 6.97 (d, J = 9.0 Hz, 1H), 3.88 (s, 3H), 3.82 (s, 3H). ^{13}C-NMR (100 MHz, $CDCl_3$): δ = 152.9 (C_q), 151.6 (q, $^3J_{C\text{–}F}$ = 1.7 Hz, C_q), 123.4 (q, $^1J_{C\text{–}F}$ = 272.5 Hz, C_q), 119.4 (q, $^2J_{C\text{–}F}$ = 31.0 Hz, C_q), 118.1 (CH), 113.6 (CH), 112.8 (q, $^3J_{C\text{–}F}$ = 5.4 Hz, CH), 56.6 (CH_3), 55.9 (CH_3). ^{19}F-NMR (470 MHz, $CDCl_3$): δ = −62.4 (s). IR (ATR): 2954, 2921, 1504, 1415, 1306, 1122, 1043 cm^{-1}. MS (EI) *m/z* (relative intensity): 206 (90) $[M]^+$, 191 (100) $[M\text{-}CH_3]^+$. HR-MS (EI) *m/z* calc. for $C_9H_9F_3O_2$ $[M]^+$: 206.0549, found: 206.0552. The analytical data correspond with those reported in the literature.[2]

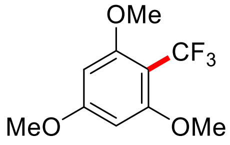

3f

1,3,5-Trimethoxy-2-(trifluoromethyl)benzene (3f)

The general procedure A was followed using **1f** (42 mg, 0.25 mmol) at 23 °C for 8 h. Purification by column chromatography on silica gel (*n*-hexane/EtOAc = 10:1) yielded **3f** (43 mg, 73%) as a white solid. M. p.: 64–65 °C. ^{1}H-NMR (400 MHz, $CDCl_3$): δ = 6.11 (s, 2H), 3.82 (s, 6H), 3.82 (s, 3H). ^{13}C-NMR (100 MHz, $CDCl_3$): δ = 163.5 (C_q), 160.4 (q, $^3J_{C\text{–}F}$ = 1.4 Hz, C_q), 124.3 (q, $^1J_{C\text{–}F}$ = 273.3 Hz, C_q), 100.4 (q, $^2J_{C\text{–}F}$ = 30.2 Hz, C_q), 91.2 (CH), 56.2 (CH_3), 55.4 (CH_3). ^{19}F-NMR (376 MHz, $CDCl_3$): δ = −54.2 (s). IR (ATR): 2951, 2920, 1594, 1460, 1417, 1276, 1204, 1090, 1025 cm^{-1}. MS (ESI) *m/z* (relative intensity): 259 (20) $[M+Na]^+$, 237 (100) $[M+H]^+$. HR-MS (ESI) *m/z* calc. for $C_{10}H_{12}F_3O_3$ $[M+H]^+$: 237.0733, found: 237.0735. The analytical data correspond with those reported in the literature.[2]

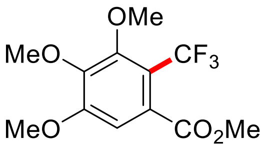

3g

Methyl 3,4,5-trimethoxy-2-(trifluoromethyl)benzoate (3g)

The general procedure A was followed using **1g** (57 mg, 0.25 mmol) at 23 °C for 16 h. Purification by column chromatography on silica gel (*n*-hexane/EtOAc = 5:1) yielded **3ag** (46 mg, 62%) as a colorless oil. ^{1}H-NMR (400 MHz, $CDCl_3$): δ = 6.73 (s, 1H), 3.93 (s, 3H), 3.89 (s, 3H), 3.87 (s, 3H), 3.87 (s, 3H). ^{13}C-NMR (100 MHz, $CDCl_3$): δ = 168.4 (C_q), 155.9 (C_q), 153.0 (q, $^{3}J_{C–F}$ = 1.6 Hz, C_q), 144.2 (C_q), 128.4 (q, $^{3}J_{C–F}$ = 2.8 Hz, C_q), 123.0 (q, $^{1}J_{C–F}$ = 273.1 Hz, C_q), 114.6 (q, $^{2}J_{C–F}$ = 31.0 Hz, C_q), 106.9 (CH), 61.9 (CH_3), 60.9 (CH_3), 56.2 (CH_3), 53.0 (CH_3). ^{19}F-NMR (376 MHz, $CDCl_3$): δ = −56.9 (s). IR (ATR): 2952, 1735, 1580, 1457, 1404, 1342, 1300, 1222, 1034 cm^{-1}. MS (ESI) *m/z* (relative intensity): 317 (100) $[M+Na]^+$. HR-MS (ESI) *m/z* calc. for $C_{12}H_{13}F_3O_5Na$ $[M+Na]^+$: 317.0607, found: 317.0611. The analytical data correspond with those reported in the literature.[3]

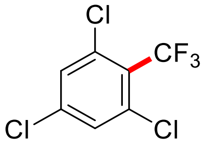

3h

1,3,5-Trichloro-2-(trifluoromethyl)benzene (3h)

The general procedure B was followed using **1h** (45 mg, 0.25 mmol) at 23 °C for 16 h. Purification by column chromatography on silica gel (pentane) yielded **3h** (31 mg, 50%) as a colorless oil. ^{1}H-NMR (400 MHz, $CDCl_3$): δ = 7.44–7.42 (m, 2H). ^{13}C-NMR (100 MHz, $CDCl_3$): δ = 138.0 (C_q), 135.3 (q, $^{3}J_{C–F}$ = 1.3 Hz, C_q), 130.6 (CH), 124.9 (q, $^{2}J_{C–F}$ = 31.3 Hz, C_q), 122.2 (q, $^{1}J_{C–F}$ = 276.2 Hz, C_q). ^{19}F-NMR (376 MHz, $CDCl_3$): δ = −55.7 (s). IR (ATR): 2956, 2925, 1580, 1567, 1375, 1283, 1209, 1142, 1114, 1030 cm^{-1}. MS (EI) *m/z* (relative intensity): 248 (100) $[M]^+$. HR-MS (EI) *m/z* calc. for $C_7H_2{}^{35}Cl_3F_3$ $[M]^+$: 247.9169, found: 247.9178. The analytical data correspond with those reported in the literature.[1]

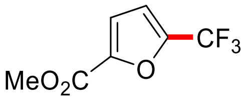

5a

Methyl 5-(trifluoromethyl)furan-2-carboxylate (5a)

The general procedure B was followed using **4a** (32 mg, 0.25 mmol) at 23 °C for 8 h. Purification by column chromatography on silica gel (*n*-hexane/EtOAc = 50:1) yielded **5a** (38 mg, 77%) as a colorless oil. ^{1}H-NMR (400 MHz, $CDCl_3$): δ = 7.18 (dq, J = 3.6, 0.9 Hz, 1H), 6.86 (dq, J = 3.6, 1.1 Hz, 1H), 3.92 (s, 3H). ^{13}C-NMR (100 MHz, $CDCl_3$): δ = 158.1 (C_q), 146.3 (q, $^{4}J_{C-F}$ = 1.4 Hz, C_q), 144.6 (q, $^{2}J_{C-F}$ = 43.3 Hz, C_q), 118.3 (q, $^{1}J_{C-F}$ = 268.2 Hz, C_q), 117.6 (CH), 112.8 (q, $^{3}J_{C-F}$ = 2.7 Hz, CH), 52.5 (CH_3). ^{19}F-NMR (376 MHz, $CDCl_3$): δ = −64.5 (s). IR (ATR): 2957, 2923, 1740, 1461, 1378, 1309, 1147 cm^{-1}. MS (ESI) *m/z* (relative intensity): 217 (20) $[M+Na]^+$, 195 (100) $[M+H]^+$. HR-MS (ESI) *m/z* calc. for $C_7H_5F_3O_3Na$ $[M+Na]^+$: 217.0083, found: 217.0086. The analytical data correspond with those reported in the literature.[1]

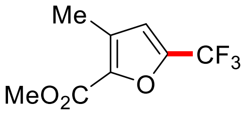

5b

Methyl 3-methyl-5-(trifluoromethyl)furan-2-carboxylate (5b)

The general procedure B was followed using **4b** (35 mg, 0.25 mmol) at 23 °C for 16 h. Purification by column chromatography on silica gel (*n*-hexane/DCM = 1:1) yielded **5b** (37 mg, 71%) as a colorless oil. ^{1}H-NMR (400 MHz, $CDCl_3$): δ = 6.74–6.69 (m, 1H), 3.90 (s, 3H), 2.36 (s, 3H). ^{13}C-NMR (100 MHz, $CDCl_3$): δ = 159.1 (C_q), 143.0 (q, $^{2}J_{C-F}$ = 43.0 Hz, C_q), 141.7 (q, $^{4}J_{C-F}$ = 1.5 Hz, C_q), 130.8 (C_q), 118.4 (q, $^{1}J_{C-F}$ = 268.3 Hz, C_q), 115.7 (q, $^{3}J_{C-F}$ = 2.6 Hz, CH), 52.0 (CH_3), 11.4 (CH_3). ^{19}F-NMR (376 MHz, $CDCl_3$): δ = −64.6 (s). IR (ATR): 2954, 2924, 1747, 1460, 1377, 1261, 1105 cm^{-1}. MS (ESI) *m/z* (relative intensity): 231 (30) $[M+Na]^+$, 209 (10) $[M+H]^+$. HR-MS (ESI) *m/z* calc. for $C_8H_8F_3O_3$ $[M+H]^+$: 209.0420, found: 209.0418.

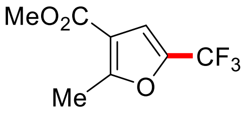

5c

Methyl 2-methyl-5-(trifluoromethyl)furan-3-carboxylate (5c)

The general procedure B was followed using **4c** (35 mg, 0.25 mmol) at 23 °C for 16 h. Purification by column chromatography on silica gel (*n*-hexane/DCM = 1:1) yielded **5c**

(33 mg, 64%) as a colorless oil. ^{1}H-NMR (400 MHz, $CDCl_3$): δ = 7.06 (s, 1H), 3.88 (s, 3H), 2.66 (s, 3H). ^{13}C-NMR (100 MHz, $CDCl_3$): δ = 163.2 (C_q), 161.5 (C_q), 139.8 (q, $^2J_{C-F}$ = 43.4 Hz, C_q), 118.7 (q, $^1J_{C-F}$ = 267.0 Hz, C_q), 114.4 (C_q), 112.6 (q, $^3J_{C-F}$ = 2.8 Hz, CH), 51.7 (CH_3), 13.8 (CH_3). ^{19}F-NMR (376 MHz, $CDCl_3$): δ = −64.5 (s). IR (ATR): 2956, 2925, 1730, 1621, 1448, 1255, 1135, 1063 cm^{-1}. MS (ESI) *m/z* (relative intensity): 231 (5) $[M+Na]^+$, 209 (20) $[M+H]^+$. HR-MS (ESI) *m/z* calc. for $C_8H_8F_3O_3$ $[M+H]^+$: 209.0420, found: 209.0426.

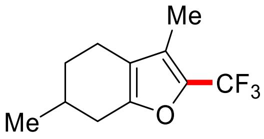

5d

3,6-Dimethyl-2-(trifluoromethyl)-4,5,6,7-tetrahydrobenzofuran (5d)

The general procedure A was followed using **4d** (38 mg, 0.25 mmol) at 23 °C for 16 h. Purification by column chromatography on silica gel (pentane) yielded **5d** (36 mg, 65%) as a colorless oil. ^{1}H-NMR (400 MHz, $CDCl_3$): δ = 2.75–2.64 (m, 1H), 2.45–2.27 (m, 2H), 2.25–2.15 (m, 1H), 2.06 (q, J = 2.0 Hz, 3H), 2.02–1.91 (m, 1H), 1.91–1.83 (m, 1H), 1.45–1.32 (m, 1H), 1.11 (d, J = 6.7 Hz, 3H). ^{13}C-NMR (100 MHz, $CDCl_3$): δ = 152.4 (q, $^4J_{C-F}$ = 1.4 Hz, C_q), 134.9 (q, $^2J_{C-F}$ = 39.9 Hz, C_q), 122.5 (q, $^3J_{C-F}$ = 2.4 Hz, C_q), 120.7 (q, $^1J_{C-F}$ = 266.9 Hz, C_q), 119.0 (C_q), 31.1 (CH_2), 30.8 (CH_2), 29.4 (CH), 21.3 (CH_3), 19.5 (CH_2), 7.9 (CH_3). ^{19}F-NMR (282 MHz, $CDCl_3$): δ = −61.2 (s). IR (ATR): 2927, 1590, 1424, 1368, 1355, 1113, 1045 cm^{-1}. MS (EI) *m/z* (relative intensity): 218 (30) $[M]^+$. HR-MS (EI) *m/z* calc. for $C_{11}H_{13}F_3O$ $[M]^+$: 218.0913, found: 218.0923.

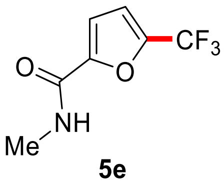

5e

N-Methyl-5-(trifluoromethyl)furan-2-carboxamide (5e)

The general procedure B was followed using **4e** (31 mg, 0.25 mmol) at 23 °C for 16 h. Purification by column chromatography on silica gel (*n*-hexane/EtOAc = 2:1) yielded **5e** (33 mg, 69%) as a white solid. M. p.: 78–80 °C. ^{1}H-NMR (500 MHz, $CDCl_3$): δ = 7.16–7.09 (m, 1H), 6.89–6.83 (m, 1H), 6.45 (brs, 1H), 2.99 (d, J = 5.0 Hz, 3H). ^{13}C-NMR (125 MHz, $CDCl_3$): δ = 157.8 (C_q), 149.7 (q, $^4J_{C-F}$ = 1.3 Hz, C_q), 142.4 (q, $^2J_{C-F}$ = 43.2 Hz, C_q), 118.4 (q,

$^{1}J_{C\text{–}F}$ = 267.8 Hz, C_q), 114.0 (CH), 113.4 (q, $^{3}J_{C\text{–}F}$ = 2.7 Hz, CH), 26.0 (CH_3). ^{19}F-NMR (470 MHz, $CDCl_3$): δ = −64.3 (s). IR (ATR): 3294, 2953, 2922, 1656, 1574, 1309, 1178, 1107, 1017 cm^{-1}. MS (ESI) *m/z* (relative intensity): 216 (100) $[M+Na]^+$, 194 (45) $[M+H]^+$. HR-MS (ESI) *m/z* calc. for $C_7H_7F_3NO_2$ $[M+H]^+$: 194.0423, found: 194.0422.

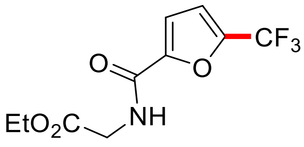

5f

Ethyl (5-(trifluoromethyl)furan-2-carbonyl)glycinate (5f)

The general procedure B was followed using **4f** (49 mg, 0.25 mmol) at 23 °C for 16 h. Purification by column chromatography on silica gel (*n*-hexane/EtOAc = 2:1) yielded **5f** (46 mg, 70%) as a white solid. M. p.: 66–68 °C. ^{1}H-NMR (400 MHz, $CDCl_3$): δ = 7.15 (dq, *J* = 3.6, 0.9 Hz, 1H), 6.90 (s, 1H), 6.87 (dq, *J* = 3.6, 1.1 Hz, 1H), 4.24 (q, *J* = 7.2 Hz, 2H), 4.19 (d, *J* = 5.4 Hz, 2H), 1.29 (t, *J* = 7.2 Hz, 3H). ^{13}C-NMR (100 MHz, $CDCl_3$): δ = 169.3 (C_q), 157.1 (C_q), 149.0 (q, $^{4}J_{C\text{–}F}$ = 1.3 Hz, C_q), 142.9 (q, $^{2}J_{C\text{–}F}$ = 43.3 Hz, C_q), 118.3 (d, $^{1}J_{C\text{–}F}$ = 267.9 Hz, C_q), 114.8 (CH), 113.4 (q, $^{3}J_{C\text{–}F}$ = 2.7 Hz, CH), 61.8 (CH_2), 41.1 (CH_2), 14.1 (CH_3). ^{19}F-NMR (376 MHz, $CDCl_3$): δ = −64.3 (s). IR (ATR): 3329, 2988, 2943, 1744, 1665, 1572, 1307, 1182, 1109, 1019 cm^{-1}. MS (ESI) *m/z* (relative intensity): 288 (100) $[M+Na]^+$, 266 (10) $[M+H]^+$. HR-MS (ESI) *m/z* calc. for $C_{10}H_{11}F_3NO_4$ $[M+H]^+$: 266.0635, found: 266.0634.

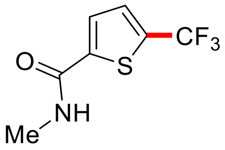

5g

***N*-Methyl-5-(trifluoromethyl)thiophene-2-carboxamide (5g)**

The general procedure B was followed using **4g** (35 mg, 0.25 mmol) at 23 °C for 16 h. Purification by column chromatography on silica gel (*n*-hexane/EtOAc = 2:1) yielded **5g** (38 mg, 73%) as a white solid. M. p.: 130–132 °C. ^{1}H-NMR (400 MHz, $CDCl_3$): δ = 7.38 (dq, *J* = 3.9, 1.1 Hz, 1H), 7.36 (dq, *J* = 3.9, 0.9 Hz, 1H), 6.24 (brs, 1H), 2.99 (d, *J* = 4.9 Hz, 3H). ^{13}C-NMR (100 MHz, $CDCl_3$): δ = 161.4 (C_q), 142.6 (C_q), 134.9 (q, $^{2}J_{C\text{–}F}$ = 38.7 Hz, C_q), 128.6 (q, $^{3}J_{C\text{–}F}$ = 3.7 Hz, CH), 126.6 (CH), 121.9 (q, $^{1}J_{C\text{–}F}$ = 269.6 Hz, C_q), 26.9 (CH_3). ^{19}F-NMR (376 MHz, $CDCl_3$): δ = −56.0 (s). IR (ATR): 3276, 2986, 1734, 1373, 1239, 1045, 913 cm^{-1}. MS (ESI) *m/z* (relative intensity): 232 (100) $[M+Na]^+$, 210 (30) $[M+H]^+$. HR-MS (ESI) *m/z* calc. for $C_7H_7F_3NOS$ $[M+H]^+$: 210.0195, found: 210.0190.

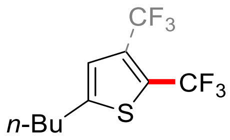

5h (ratio: 10/1)

2-Butyl-5-(trifluoromethyl)thiophene (5h)

The ratio of two mono-substituted products was determined to be 10:1 by ^{1}H-NMR analysis of the crude reaction mixture. The general procedure A was followed using **4h** (35 mg, 0.25 mmol) at 23 °C for 16 h. Purification by column chromatography on silica gel (*n*-hexane) yielded **5h** (34 mg, 65%) as a colorless oil. ^{1}H-NMR (300 MHz, $CDCl_3$): δ = 7.28–7.25 (m, 1H), 6.78–6.73 (m, 1H), 2.85 (t, J = 7.6 Hz, 2H), 1.75–1.64 (m, 2H), 1.49–1.35 (m, 2H), 0.97 (t, J = 7.3 Hz, 3H). ^{13}C-NMR (100 MHz, $CDCl_3$): δ = 150.3 (q, $^{4}J_{C-F}$ = 1.3 Hz, C_q), 128.4 (q, $^{3}J_{C-F}$ = 3.8 Hz, CH), 128.3 (q, $^{2}J_{C-F}$ = 38.2 Hz, C_q), 123.8 (CH), 122.6 (q, $^{1}J_{C-F}$ = 268.1 Hz, C_q), 33.6 (CH_2), 29.7 (CH_2), 22.1 (CH_2), 13.7 (CH_3). ^{19}F-NMR (282 MHz, $CDCl_3$): δ = −55.1 (s). IR (ATR): 2957, 2924, 1481, 1378, 1299, 1154, 1125, 1051 cm^{-1}. MS (EI) *m/z* (relative intensity): 208 (30) $[M]^+$, 165 (100). HR-MS (EI) *m/z* calc. for $C_9H_{11}F_3S$ $[M]^+$: 208.0534, found: 208.0526.

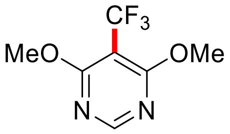

5i

4,6-Dimethoxy-5-(trifluoromethyl)pyrimidine (5i)

The general procedure B was followed using **4i** (35 mg, 0.25 mmol) at 23 °C for 15 h. Purification by column chromatography on silica gel (*n*-hexane/EtOAc = 30:1) yielded **5i** (32 mg, 62%) as a white solid. M. p.: 94–96 °C. ^{1}H-NMR (400 MHz, $CDCl_3$): δ = 8.45 (s, 1H), 4.02 (s, 6H). ^{13}C-NMR (100 MHz, $CDCl_3$): δ = 167.9 (C_q), 158.9 (CH), 122.8 (q, $^{1}J_{C-F}$ = 272.9 Hz, C_q), 95.4 (q, $^{2}J_{C-F}$ = 33.8 Hz, C_q), 55.0 (CH_3). ^{19}F-NMR (376 MHz, $CDCl_3$): δ = −56.9 (s). IR (ATR): 2954, 2924, 1573, 1476, 1386, 1244, 1099, 1034 cm^{-1}. MS (ESI) *m/z* (relative intensity): 231 (50) $[M+Na]^+$, 209 (100) $[M+H]^+$. HR-MS (ESI) *m/z* calc. for $C_7H_8F_3N_2O_2$ $[M+H]^+$: 209.0538, found: 209.0540. The analytical data correspond with those reported in the literature.[1]

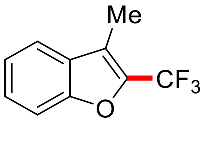

5j

3-Methyl-2-(trifluoromethyl)benzofuran (5j)

The general procedure A was followed using **4j** (33 mg, 0.25 mmol) at 23 °C for 8 h. Purification by column chromatography on silica gel (*n*-hexane) yielded **5j** (36 mg, 72%) as a colorless oil. ^{1}H-NMR (400 MHz, $CDCl_3$): δ = 7.59 (d, J = 7.8 Hz, 1H), 7.52–7.48 (m, 1H), 7.45–7.39 (m, 1H), 7.31 (ddd, J = 7.8, 7.2, 1.0 Hz, 1H), 2.39 (q, J = 2.1 Hz, 3H). ^{13}C-NMR (100 MHz, $CDCl_3$): δ = 154.0 (C_q), 138.5 (q, $^2J_{C\text{–}F}$ = 39.8 Hz, C_q), 128.4 (C_q), 126.9 (CH), 123.3 (CH), 120.6 (CH), 120.5 (q, $^1J_{C\text{–}F}$ = 267.0 Hz, C_q), 118.2 (q, $^3J_{C\text{–}F}$ = 2.7 Hz, C_q), 111.9 (CH), 7.7 (q, $^4J_{C\text{–}F}$ = 1.0 Hz, CH_3). ^{19}F-NMR (376 MHz, $CDCl_3$): δ = −62.0 (s). IR (ATR): 2956, 2925, 1635, 1454, 1385, 1302, 1129, 1083, 1041 cm^{-1}. MS (EI) *m/z* (relative intensity): 200 (100) $[M]^+$. HR-MS (EI) *m/z* calc. for $C_{10}H_7F_3O$ $[M]^+$: 200.0444, found: 200.0443. The analytical data correspond with those reported in the literature.[1]

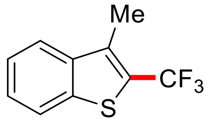

5k

3-Methyl-2-(trifluoromethyl)benzo[*b*]thiophene (5k)

The general procedure A was followed using **4k** (37 mg, 0.25 mmol) at 23 °C for 8 h. Purification by column chromatography on silica gel (*n*-hexane) yielded **5k** (35 mg, 65%) as a colorless oil. ^{1}H-NMR (400 MHz, $CDCl_3$): δ = 7.86–7.81 (m, 1H), 7.80–7.76 (m, 1H), 7.48–7.41 (m, 2H), 2.55 (q, J = 1.8 Hz, 3H). ^{13}C-NMR (100 MHz, $CDCl_3$): δ = 139.6 (C_q), 138.5 (q, $^4J_{C\text{–}F}$ = 1.0 Hz, C_q), 134.7 (q, $^3J_{C\text{–}F}$ = 3.3 Hz, C_q), 126.5 (CH), 124.9 (q, $^2J_{C\text{–}F}$ = 31.4 Hz, C_q), 124.8 (CH), 123.2 (q, $^1J_{C\text{–}F}$ = 270.5 Hz, C_q), 123.0 (CH), 122.6 (CH), 11.9 (CH_3). ^{19}F-NMR (282 MHz, $CDCl_3$): δ = −54.1 (s). IR (ATR): 2957, 2928, 1579, 1438, 1359, 1290, 1120, 989 cm^{-1}. MS (EI) *m/z* (relative intensity): 216 (100) $[M]^+$. HR-MS (EI) *m/z* calc. for $C_{10}H_7F_3O$ $[M]^+$: 216.0215, found: 216.0212. The analytical data correspond with those reported in the literature.[1]

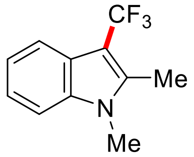

5l

1,2-Dimethyl-3-(trifluoromethyl)-1*H*-indole (5l)

The general procedure A was followed using **4l** (36 mg, 0.25 mmol) at 23 °C for 16 h. Purification by column chromatography on silica gel (*n*-hexane/EtOAc = 30:1) yielded **5l**

(30 mg, 57%) as a colorless oil. ^{1}H-NMR (400 MHz, $CDCl_3$): δ= 7.72–7.67 (m, 1H), 7.30–7.26 (m, 1H), 7.25–7.20 (m, 1H), 7.19–7.14 (m, 1H), 3.67 (s, 3H), 2.52 (q, J = 1.4 Hz, 3H). ^{13}C-NMR (100 MHz, $CDCl_3$): δ= 137.2 (q, $^{3}J_{C\text{–}F}$ = 3.8 Hz, C_q), 136.1 (C_q), 125.5 (q, $^{1}J_{C\text{–}F}$ = 266.7 Hz, C_q), 124.4 (q, $^{3}J_{C\text{–}F}$ = 1.8 Hz, C_q), 122.0 (CH), 121.0 (CH), 119.0 (CH), 109.2 (CH), 102.6 (q, $^{2}J_{C\text{–}F}$ = 35.2 Hz, C_q), 29.5 (CH_3), 10.9 (q, $^{4}J_{C\text{–}F}$ = 1.8 Hz, CH_3). ^{19}F-NMR (376 MHz, $CDCl_3$): δ= −53.7 (s). IR (ATR): 2949, 2926, 1617, 1558, 1475, 1416, 1283, 1226, 1076 cm^{-1}. MS (ESI) m/z (relative intensity): 236 (100) $[M+Na]^+$, 214 (40) $[M+H]^+$. HR-MS (ESI) m/z calc. for $C_{11}H_{11}F_3N$ $[M+H]^+$: 214.0838, found: 214.0833. The analytical data correspond with those reported in the literature.[2]

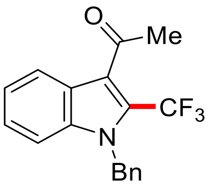

5m

1-(1-Benzyl-2-(trifluoromethyl)-1*H*-indol-3-yl)ethan-1-one (5m)

The general procedure B was followed using **4m** (62 mg, 0.25 mmol) at 23 °C for 16 h. Purification by column chromatography on silica gel (*n*-hexane/EtOAc = 15:1) yielded **5m** (45 mg, 57%) as a colorless oil. ^{1}H-NMR (400 MHz, $CDCl_3$): δ= 7.94 (d, J = 8.0 Hz, 1H), 7.39–7.27 (m, 6H), 7.07–7.00 (m, 2H), 5.6 (s, 2H), 2.7 (s, 3H). ^{13}C-NMR (100 MHz, $CDCl_3$): δ= 196.8 (C_q), 137.0 (C_q), 136.0 (C_q), 128.9 (CH), 127.8 (CH), 125.8 (CH), 125.8 (CH), 125.3 (d, $^{2}J_{C\text{–}F}$ = 37.5 Hz), 124.8 (C_q), 122.8 (CH), 121.9 (CH), 121.1 (q, $^{1}J_{C\text{–}F}$ = 268.9 Hz, C_q), 120.4 (q, $^{3}J_{C\text{–}F}$ = 2.7 Hz, C_q), 111.0 (CH), 48.8 (q, $^{4}J_{C\text{–}F}$ = 2.9 Hz, CH_2), 32.0 (q, $^{5}J_{C\text{–}F}$ = 3.0 Hz, CH_3). ^{19}F-NMR (282 MHz, $CDCl_3$): δ= −54.3 (s). IR (ATR): 2956, 2925, 1680, 1541, 1416, 1352, 1249, 1226, 1164, 1117, 1089 cm^{-1}. MS (ESI) m/z (relative intensity): 340 (100) $[M+Na]^+$, 318 (30) $[M+H]^+$. HR-MS (ESI) m/z calc. for $C_{18}H_{15}F_3NO$ $[M+H]^+$: 318.1100, found: 318.1097.

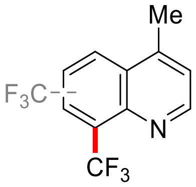

5n (ratio: 20/1)

4-Methyl-2-(trifluoromethyl)quinoline (5n)

The ratio of two mono-substituted products was determined to be 20:1 by ^{1}H-NMR analysis of the crude reaction mixture, while the exact substituent position in the minor component could not be identified. The general procedure A was followed using **4n** (36 mg, 0.25 mmol) at 23 °C for 15 h. Purification by column chromatography on silica gel (*n*-hexane/EtOAc = 10:1) yielded **5n** (29 mg, 55%) as a colorless oil. ^{1}H-NMR (500 MHz, $CDCl_3$): δ = 8.92 (d, J = 4.4 Hz, 1H), 8.20 (dd, J = 8.4, 0.8 Hz, 1H), 8.06 (ddq, J = 7.4, 1.5, 0.8 Hz, 1H), 7.60 (ddd, J = 8.4, 7.4, 0.8 Hz, 1H), 7.32 (dq, J = 4.4, 1.0 Hz, 1H), 2.73 (d, J = 1.0 Hz, 3H). ^{13}C-NMR (125 MHz, $CDCl_3$): δ = 150.9 (CH), 144.6 (q, $^3J_{C\text{–}F}$ = 3.2 Hz, C_q), 144.6 (C_q), 128.8 (C_q), 128.4 (q, $^4J_{C\text{–}F}$ = 0.8 Hz, CH), 128.2 (q, $^2J_{C\text{–}F}$ = 29.2 Hz, C_q), 127.6 (q, $^3J_{C\text{–}F}$ = 5.7 Hz, CH), 124.8 (CH), 124.2 (q, $^1J_{C\text{–}F}$ = 273.4 Hz, C_q), 122.7 (CH), 19.0 (CH_3). ^{19}F-NMR (470 MHz, $CDCl_3$): δ = −60.0 (s). IR (ATR): 2954, 2925, 1601, 1513, 1315, 1296, 1133, 1092 cm^{-1}. MS (ESI) *m/z* (relative intensity): 234 (95) $[M+Na]^+$, 212 (100) $[M+H]^+$. HR-MS (ESI) *m/z* calc. for $C_{11}H_9F_3N$ $[M+H]^+$: 212.0682, found: 212.0682.

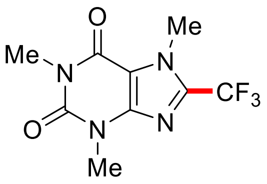

6

1,3,7-Trimethyl-8-(trifluoromethyl)-3,7-dihydro-1*H*-purine-2,6-dione (6)

The general procedure A was followed using caffeine (49 mg, 0.25 mmol) at 23 °C for 8 h. Purification by column chromatography on silica gel (*n*-hexane/EtOAc = 3:1) yielded **6** (46 mg, 70%) as a white solid. M. p.: 128–130 °C. ^{1}H-NMR (400 MHz, $CDCl_3$): δ = 4.13 (q, J = 1.2 Hz, 3H), 3.56 (s, 3H), 3.39 (s, 3H). ^{13}C-NMR (100 MHz, $CDCl_3$): δ = 155.4 (C_q), 151.3 (C_q), 146.5 (C_q), 138.9 (q, $^2J_{C\text{–}F}$ = 40.0 Hz, C_q), 118.2 (q, $^1J_{C\text{–}F}$ = 271.3 Hz, C_q), 109.6 (C_q), 33.2 (q, $^4J_{C\text{–}F}$ = 1.9 Hz, CH_3), 29.9 (CH_3), 28.2 (CH_3). ^{19}F-NMR (376 MHz, $CDCl_3$): δ = −62.4 (s). IR (ATR): 2957, 2927, 1708, 1662, 1548, 1460, 1428, 1243, 1141, 1098 cm^{-1}. MS (ESI) *m/z* (relative intensity): 285 (80) $[M+Na]^+$, 263 (100) $[M+H]^+$. HR-MS (ESI) *m/z* calc. for $C_9H_{10}F_3N_4O_2$ $[M+H]^+$: 263.0750, found: 263.0751. The analytical data correspond with those reported in the literature.[2]

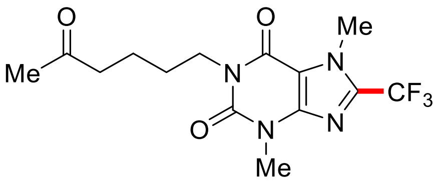

7

3,7-Dimethyl-1-(5-oxohexyl)-8-(trifluoromethyl)-3,7-dihydro-1*H*-purine-2,6-dione (7)

The general procedure A was followed using Pentoxifylline (70 mg, 0.25 mmol) at 23 °C for 16 h. Purification by column chromatography on silica gel (*n*-hexane/EtOAc = 1:1) yielded **7** (63 mg, 72%) as a colorless oil. ^{1}H-NMR (400 MHz, $CDCl_3$): δ = 4.11 (q, *J* = 1.2 Hz, 3H), 3.98 (t, *J* = 8.0 Hz, 2H), 3.54 (s, 3H), 2.46 (t, *J* = 6.9 Hz, 2H), 2.10 (s, 3H), 1.67–1.56 (m, 4H). ^{13}C-NMR (100 MHz, $CDCl_3$): δ = 208.5 (C_q), 155.3 (C_q), 151.0 (C_q), 146.5 (C_q), 138.9 (q, $^{2}J_{C\text{–}F}$ = 40.1 Hz, C_q), 118.2 (q, $^{1}J_{C\text{–}F}$ = 271.4 Hz, C_q), 109.6 (C_q), 43.0 (CH_2), 41.1 (CH_2), 33.1 (q, $^{4}J_{C\text{–}F}$ = 2.0 Hz, CH_3), 29.9 (CH_3), 29. 8 (CH_3), 27.3 (CH_2), 20.8 (CH_2). ^{19}F-NMR (376 MHz, $CDCl_3$): δ = −62.4 (s). IR (ATR): 2957, 1708, 1661, 1609, 1547, 1462, 1334, 1247, 1130, 1098 cm^{-1}. MS (ESI) *m/z* (relative intensity): 369 (100) $[M+Na]^+$, 347 (20) $[M+H]^+$. HR-MS (ESI) *m/z* calc. for $C_{14}H_{18}F_3N_4O_3$ $[M+H]^+$: 347.1326, found: 347.1320. The analytical data correspond with those reported in the literature.[2]

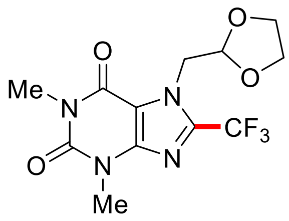

8

7-((1,3-Dioxolan-2-yl)methyl)-1,3-dimethyl-8-(trifluoromethyl)-3,7-dihydro-1*H*-purine-2,6-dione (8)

The general procedure A was followed using Doxofylline (67 mg, 0.25 mmol) at 23 °C for 16 h. Purification by column chromatography on silica gel (*n*-hexane/EtOAc = 2:1) yielded **8** (55 mg, 65%) as a colorless oil. ^{1}H-NMR (400 MHz, $CDCl_3$): δ = 5.31 (t, *J* = 4.3 Hz, 1H), 4.65 (dd, *J* = 4.3, 1.0 Hz, 2H), 3.96–3.83 (m, 4H), 3.57 (s, 3H), 3.39 (s, 3H). ^{13}C-NMR (100 MHz, $CDCl_3$): δ = 155.3 (C_q), 151.3 (C_q), 146. 7 (C_q), 139.1 (q, $^{2}J_{C\text{–}F}$ = 40.0 Hz, C_q), 118.2 (q, $^{1}J_{C\text{–}F}$ = 271.6 Hz, C_q), 109.5 (C_q), 100.9 (CH), 65.3 (CH_2), 48.6 (q, $^{4}J_{C\text{–}F}$ = 1.5 Hz, CH_2), 29.9 (CH_3), 28.3 (CH_3). ^{19}F-NMR (376 MHz, $CDCl_3$): δ = −62.4 (s). IR (ATR): 2957, 2896, 1710, 1661, 1612, 1545, 1455, 1346, 1267, 1128, 1038 cm^{-1}. MS (ESI) *m/z* (relative intensity): 357

(100) [M+Na]$^+$, 335 (15) [M+H]$^+$. HR-MS (ESI) *m/z* calc. for $C_{12}H_{14}F_3N_4O_4$ [M+H]$^+$: 335.0962, found: 335.0970.

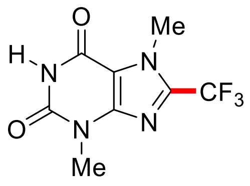

9

3,7-Dimethyl-8-(trifluoromethyl)-3,7-dihydro-1*H*-purine-2,6-dione (9)

The general procedure A was followed using Theobromine (45 mg, 0.25 mmol) at 23 °C for 16 h. Purification by column chromatography on silica gel (*n*-hexane/EtOAc = 1:1) yielded **9** (28 mg, 45%) as a white solid. M. p.: 206–208 °C (dark, decomposed). ^{1}H-NMR (500 MHz, $CDCl_3$): δ = 8.45 (s, 1H), 4.12 (q, J = 1.2 Hz, 3H), 3.53 (s, 3H). ^{13}C-NMR (125 MHz, $CDCl_3$): δ = 154.6 (C_q), 150.5 (C_q), 148.4 (C_q), 139.5 (q, $^2J_{C-F}$ = 40.3 Hz, C_q), 118.0 (q, $^1J_{C-F}$ = 271.4 Hz, C_q), 109.9 (C_q), 33.3 (q, $^4J_{C-F}$ = 1.9 Hz, CH_3), 29.2 (CH_3). ^{19}F-NMR (470 MHz, $CDCl_3$): δ = −62.5 (s). IR (ATR): 3169, 2958, 2924, 1701, 1548, 1351, 1247, 1194, 1141, 1102 cm^{-1}. MS (ESI) *m/z* (relative intensity): 271 (30) [M+Na]$^+$, 249 (100) [M+H]$^+$. HR-MS (ESI) *m/z* calc. for $C_8H_8F_3N_4O_2$ [M+H]$^+$: 249.0594, found: 249.0602. The analytical data correspond with those reported in the literature.[4]

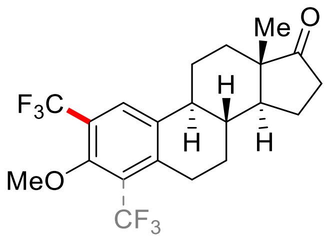

10 (ratio: 3/1)

(*8R,9S,13S,14S*)-3-Methoxy-13-methyl-2-(trifluoromethyl)-6,7,8,9,11,12,13,14,15,16-decahydro-17*H*-cyclopenta[*a*]phenanthren-17-one (10)

The ratio of two mono-substituted products was determined to be 3:1 by ^{1}H-NMR analysis of the crude reaction mixture. The general procedure B was followed using Methyl Estrone (71 mg, 0.25 mmol) at 23 °C for 16 h. Purification by column chromatography on silica gel (*n*-hexane) yielded **10** (48 mg, 55%) as a yellow oil. The ratio was determined to be 10:1 by ^{1}H-NMR analysis after column. ^{1}H-NMR (400 MHz, $CDCl_3$): δ = 7.44 (s, 1H), 6.69 (s, 1H), 3.84 (s, 3H), 2.96–2.87 (m, 2H), 2.54–2.45 (m, 1H), 2.43–2.37 (m, 1H), 2.29–2.22 (m, 1H), 2.13 (dt, J = 18.8, 8.7 Hz, 1H), 2.08–2.00 (m, 2H), 1.98–1.93 (m, 1H), 1.67–1.55 (m, 2H),

1.54–1.41 (m, 4H), 0.89 (s, 3H). ^{13}C-NMR (100 MHz, $CDCl_3$): δ = 220.5 (C_q), 155.2 (q, $^3J_{C\text{–}F}$ = 1.4 Hz, C_q), 142.1 (C_q), 131.5 (C_q), 124.1 (q, $^3J_{C\text{–}F}$ = 5.2 Hz, CH), 124.0 (q, $^1J_{C\text{–}F}$ = 272.0 Hz, C_q), 116.2 (q, $^2J_{C\text{–}F}$ = 30.5 Hz, C_q), 112.3 (CH), 55.9 (CH_3), 50.3 (CH), 47.9 (C_q), 43.7 (CH), 38.1 (CH), 35.8 (CH_2), 31.4 (CH_2), 29.8 (CH_2), 26.2 (CH_2), 25.8 (CH_2), 21.5 (CH_2), 13.8 (CH_3). ^{19}F-NMR (376 MHz, $CDCl_3$): δ = −61.8 (s). IR (ATR): 2933, 1737, 1621, 1507, 1465, 1416, 1255, 1117, 1051 cm^{-1}. MS (ESI) *m/z* (relative intensity): 375 (100) $[M+Na]^+$, 353 (30) $[M+H]^+$. HR-MS (ESI) *m/z* calc. for $C_{20}H_{24}F_3O_2$ $[M+H]^+$: 353.1723, found: 353.1717. The analytical data correspond with those reported in the literature.[2]

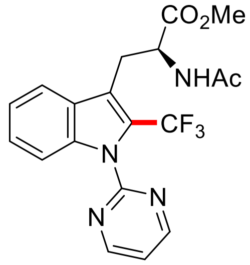

11

Methyl (*S*)-2-acetamido-3-(1-(pyrimidin-2-yl)-2-(trifluoromethyl)-1*H*-indol-3-yl)propanoate (11)

The general procedure B was followed using Tryptophan derivative (85 mg, 0.25 mmol) at 23 °C for 16 h. Purification by column chromatography on silica gel (*n*-hexane/EtOAc = 1:1) yielded **11** (65 mg, 64%) as a colorless oil. ^{1}H-NMR (300 MHz, $CDCl_3$): δ = 8.88 (d, *J* = 4.8 Hz, 2H), 8.01 (dt, *J* = 8.5, 1.0 Hz, 1H), 7.84 (d, *J* = 7.6 Hz, 1H), 7.42 (ddd, *J* = 8.5, 7.1, 1.2 Hz, 1H), 7.36–7.27 (m, 2H), 6.22 (d, *J* = 7.9 Hz, 1H), 5.02 (dt, *J* = 7.9, 6.4 Hz, 1H), 3.68 (s, 3H), 3.59–3.50 (m, 2H), 2.01 (s, 3H). ^{13}C-NMR (100 MHz, $CDCl_3$): δ = 172.0 (C_q), 169.8 (C_q), 158.5 (CH), 157.2 (C_q), 137.0 (C_q), 127.7 (C_q), 126.6 (CH), 124.2 (q, $^2J_{C\text{–}F}$ = 36.4 Hz, C_q), 122.7 (CH), 121.8 (q, $^1J_{C\text{–}F}$ = 268.0 Hz, C_q), 120.3 (CH), 118.9 (q, $^3J_{C\text{–}F}$ = 2.7 Hz, C_q), 118.8 (CH), 113.2 (CH), 52.43 (CH), 52.37 (CH_3), 27.6 (q, $^4J_{C\text{–}F}$ = 1.8 Hz, CH_2), 23.1 (CH_3). ^{19}F-NMR (282 MHz, $CDCl_3$): δ = −52.9 (s). IR (ATR): 3290, 3056, 2955, 1744, 1657, 1567, 1423, 1287, 1087 cm^{-1}. MS (ESI) *m/z* (relative intensity): 429 (100) $[M+Na]^+$, 407 (15) $[M+H]^+$. HR-MS (ESI) *m/z* calc. for $C_{19}H_{18}F_3N_4O_3$ $[M+H]^+$: 407.1326, found: 407.1321.

Some inert examples

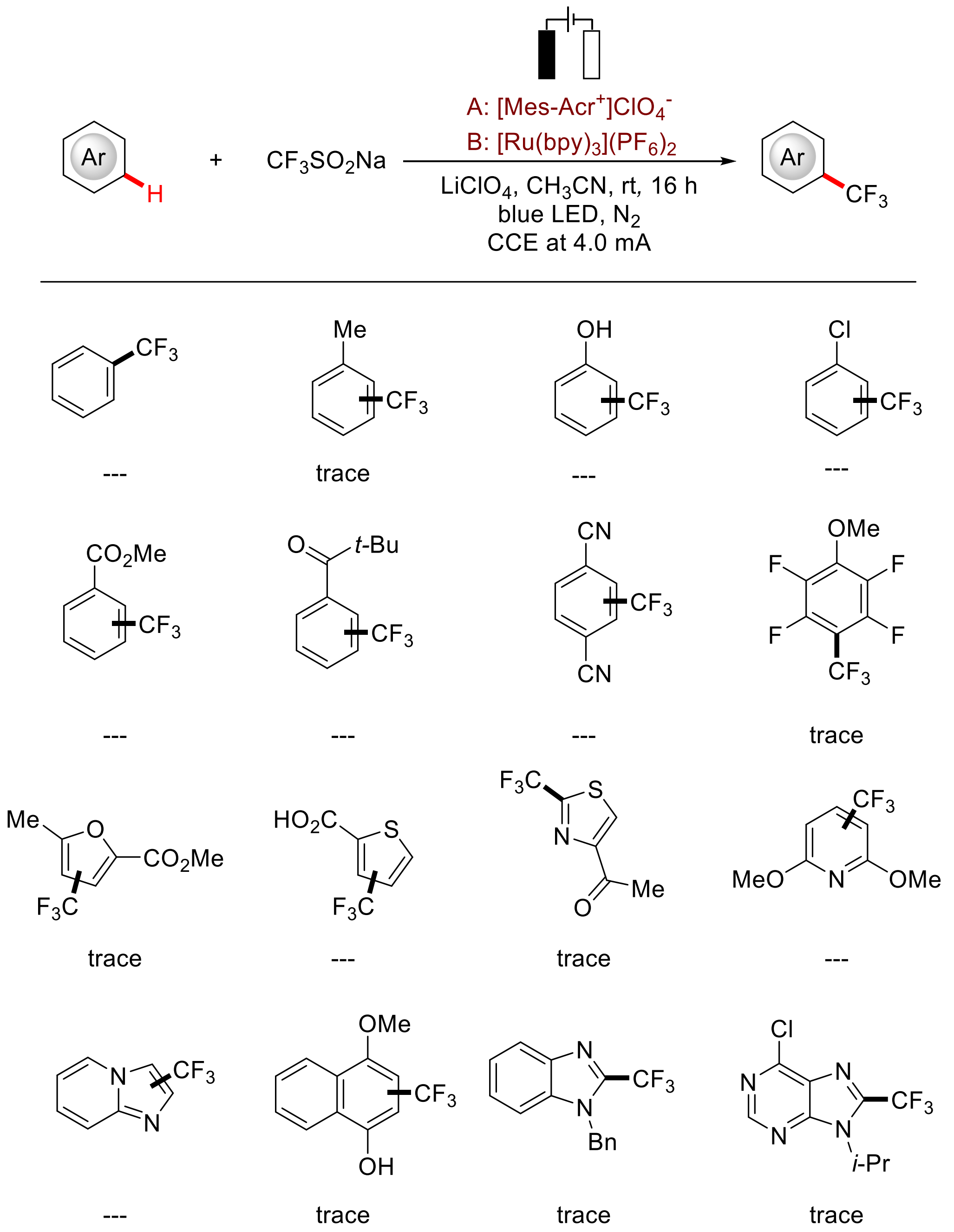

General Procedure for the Electrophotochemical C–H Trifluoromethylation in Flow

A 15 mL-Schlenk tube was charged with Substrate **1a** (60 mg, 0.5 mmol, 1.0 equiv), CF_3SO_2Na **2** (156 mg, 1.0 mmol, 2.0 equiv), $LiClO_4$ (85 mg, 0.80 mmol) and [Mes-Acr$^+$]ClO_4^- (5.1 mg, 5.0 mol %) and CH_3CN (10.0 mL) under N_2. The tube was sealed with a septum and connected to a balloon filled with N_2. The solution was passed through the electroflow reactor and a following transparent FEP tube (ID 0.5 mm, OD 1/16'') by a peristaltic pump with a flow speed of 1.0 mL/min. Ca. 20 cm of the FEP tube were irradiated. The electrophotocatalysis was performed at 23 °C with a constant current of 4.0 mA maintained for 12 h under visible light irradiation (2 × Kessil A360N lamp). Graphite felt was washed by pumping through additional 10 mL of methanol. After dismantling the reactor, the graphite felt anode was washed with CH_2Cl_2 (3 × 20 mL) in an ultrasonic bath. Evaporation of the solvents and subsequent column chromatography on silica gel (pentane) afforded the corresponding products **3a** (71 mg, 76%). With 2 mol % of $[Ru(bipy)_3](PF_6)_2$ (B) as the catalyst, 3a was obtained in 15% yield.

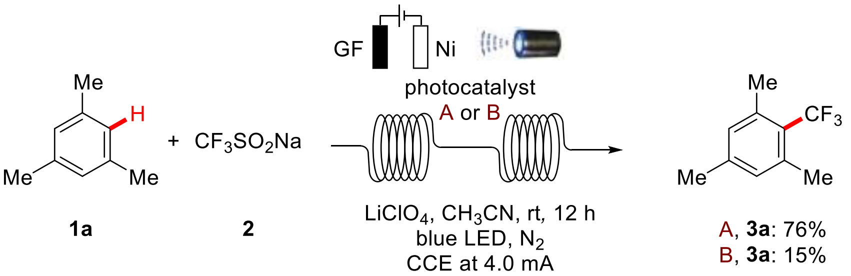

Description of the employed electro-flow-reactor:

Electrocatalysis in flow was designed based on a commercial IKA ElectraSyn flow. The nickel cathode **A** and gasket **D** were used directly. Turbulence promoter **B** and anode slot **E** for the graphite felt anode **C** were made from polytetrafluoroethylene (PTFE).

Flow Reactor Compartments: **A**: nickel cathode (Teflon base); **B**: turbulence promoter; **C**: graphite felt (1.9 cm × 5.9 cm × 0.6 cm); **D**: gasket; **E**: slot for graphite felt (Teflon base).

3D-explosion drawing of the flow cell setup:

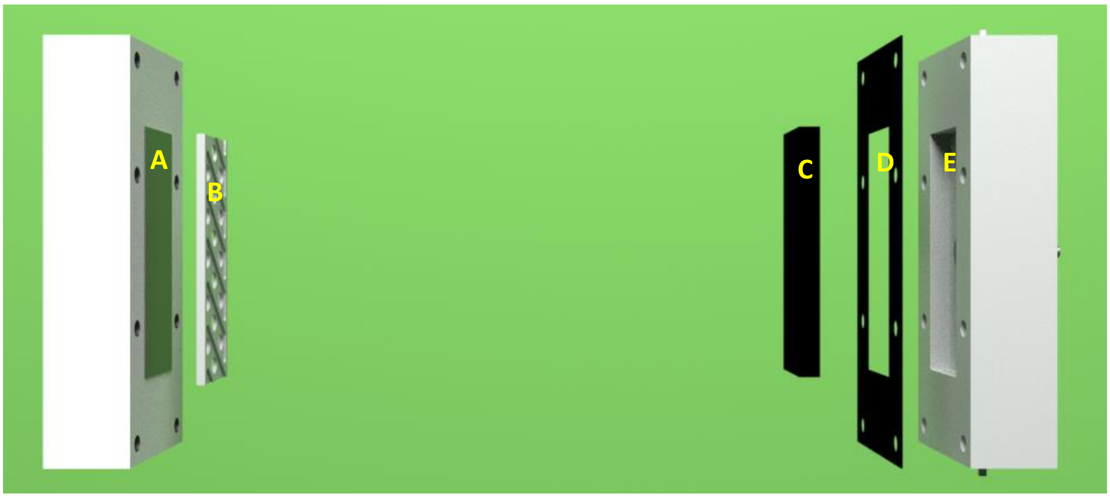

Dimensions of B: length: 5.7 cm. width: 1.9 cm; thickness: 0.2 cm (0.1 cm).

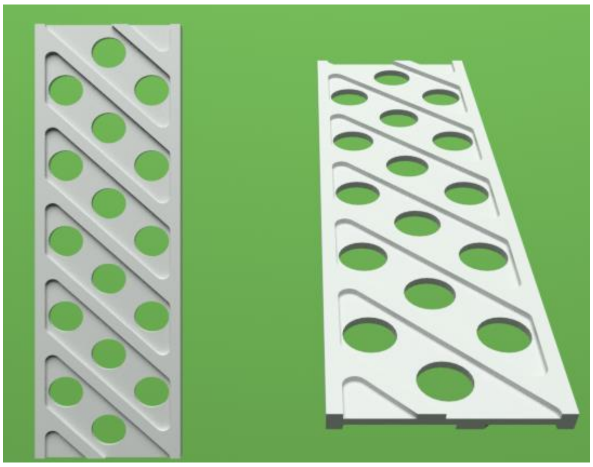

For further details of the electro-flow-reactor components, please see our recently published work.[5]

On-Line NMR Monitoring in Flow

The ^{19}F and ^{1}H NMR spectroscopy experiments in flow were performed on a Magritek Spinsolve 60^{ULTRA} (from Magritek GmbH, Germany) with the reaction monitoring kit supplied by the manufacturer. For pumping the solution to the spectrometer, an Ismatec REGLO Digital MS-2/12 (ISM 596) peristaltic pump was employed. The flow rate was 0.4 mL/min.

Reaction A: A 15 mL-Schlenk tube was charged with Substrate **1a** (60 mg, 0.375 mmol, 1.0 equiv), CF_3SO_2Na **2** (156 mg, 0.75 mmol, 2.0 equiv), $LiClO_4$ (63 mg, 0.59 mmol) and [Mes-Acr$^+$]ClO_4^- (5.1 mg, 5.0 mol %) and CH_3CN (8.0 mL) under N_2. The tube was sealed with a septum and connected to a balloon filled with N_2. The solution was pumped to the NMR spectrometer by a peristaltic pump with a flow speed of 0.4 mL/min. The electrophotocatalysis was performed at 23 °C with a constant current of 4.0 mA maintained for 12 h under visible light irradiation (2 × Kessil A360N lamp). Subsequently the reaction yield was determined by ^{1}H NMR with CH_2Br_2 as internal standard and the obtained value employed for calibration of the NMR spectra recorded in flow.

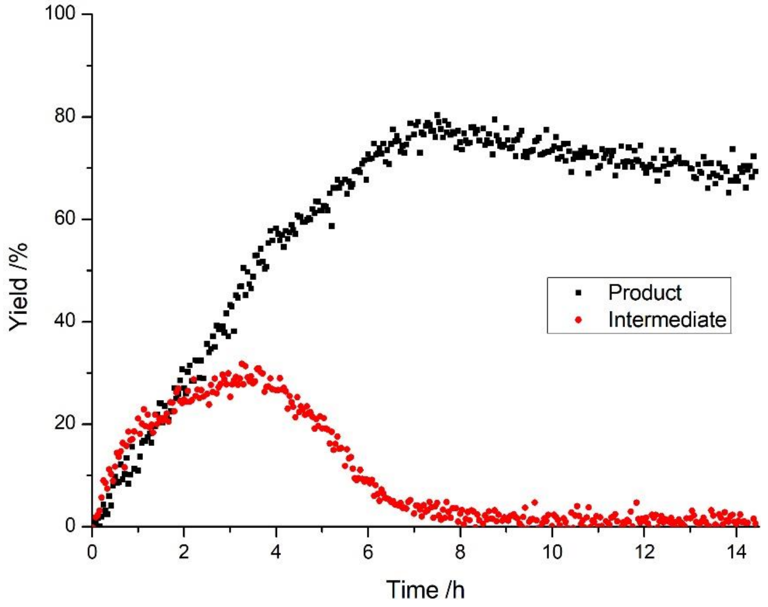

Figure S-2 Reaction profile of reaction A determined by ^{19}F NMR spectroscopic monitoring in flow.

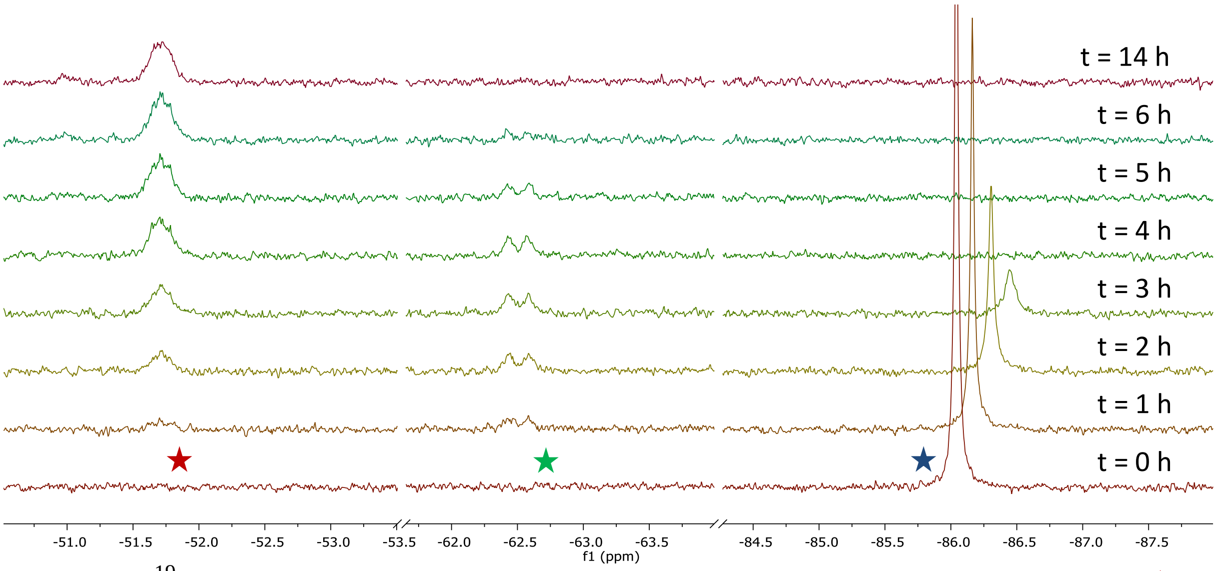

Figure S-3 ^{19}F NMR spectra recorded in flow from reaction mixture A at selected times (★: Product, ★: Intermediate, ★: $NaSO_2CF_3$).

Reaction B: In a second experiment a 15 mL-Schlenk tube was charged with Substrate **1a** (60 mg, 0.5 mmol, 1.0 equiv), CF_3SO_2Na **2** (156 mg, 1.0 mmol, 2.0 equiv), $LiClO_4$ (84.4 mg, 0.80 mmol) and [Mes-Acr$^+$]ClO_4^- (5.1 mg, 5.0 mol %) and CH_3CN (8.0 mL) under N_2. The tube was sealed with a septum and connected to a balloon filled with N_2. The solution was pumped to the NMR spectrometer by a peristaltic pump with a flow speed of 0.4 mL/min. The electrophotocatalysis was performed at 23 °C with a constant current of 4.0 mA maintained for 12 h under visible light irradiation (2 × Kessil A360N lamp). Subsequently the reaction yield was determined by ^{1}H NMR with CH_2Br_2 as internal standard and the obtained value employed for calibration of the NMR spectra recorded in flow.

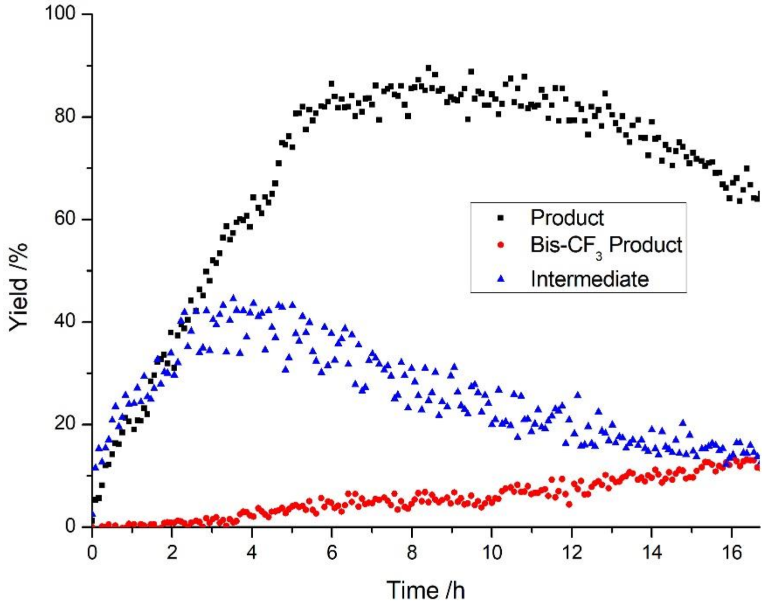

Figure S-4 Reaction profile of reaction B determined by ^{19}F NMR spectroscopic monitoring in flow.

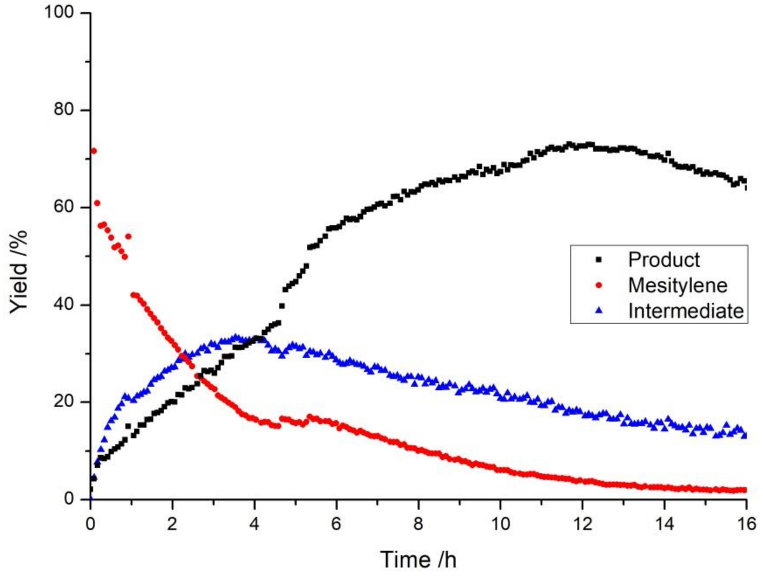

Figure S-5 Reaction profile of reaction B determined by ^{1}H NMR spectroscopic monitoring in flow.

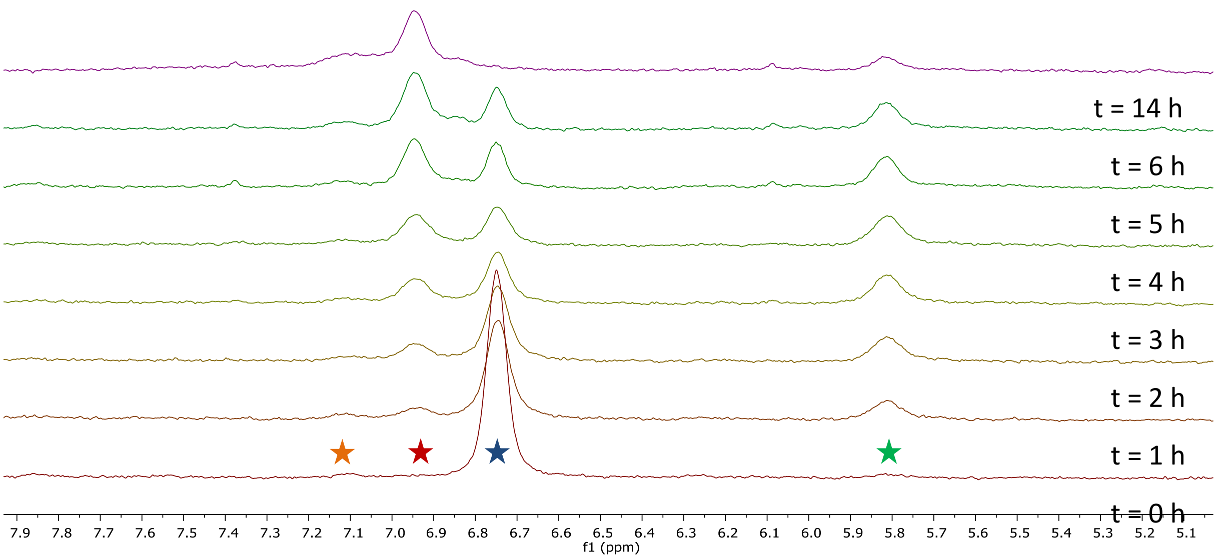

Figure S-6 ^{1}H NMR spectra recorded in flow from reaction mixture B at selected times (★: Bis-CF_3-Product, ★: Product, ★: Mesitylene, ★: Intermediate).

Radical trap experiments

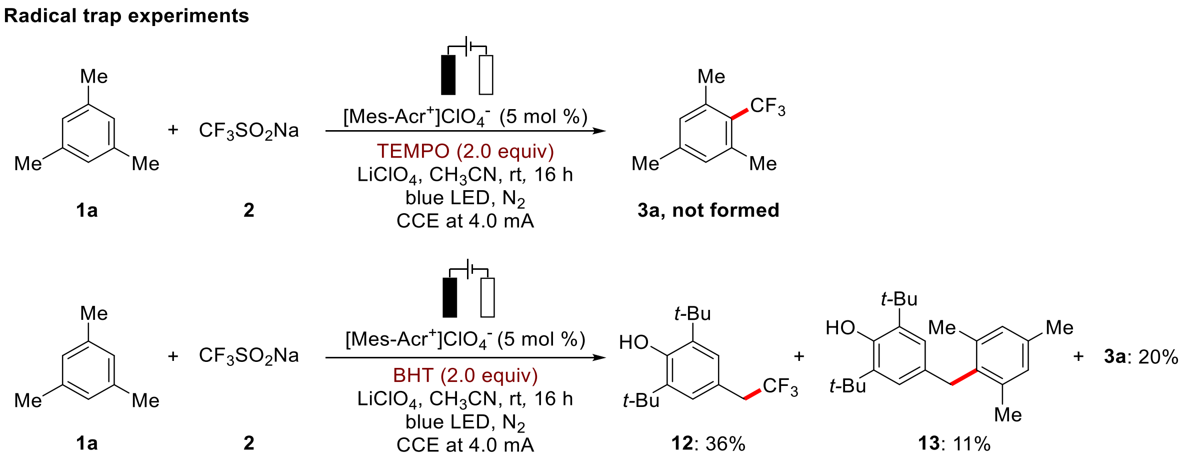

The electrophotocatalysis was carried out in an undivided cell, with a GF anode (10 mm × 15 mm × 6 mm) and a Pt cathode (10 mm × 15 mm × 0.25 mm). **1a** (30 mg, 0.25 mmol), CF_3SO_2Na (**2**, 78 mg, 0.50 mmol), $LiClO_4$ (42 mg, 0.40 mmol), $[Mes\text{-}Acr^+]ClO_4^-$ (5.1 mg, 5.0 mol %) and BHT (110 mg, 0.50 mmol) were dissolved in CH_3CN (4.0 mL) under N_2. The electrophotocatalysis was performed at 23 °C with a constant current of 4.0 mA maintained for 16 h under visible light irradiation (2 × Kessil A360N). The GF anode was washed with CH_2Cl_2 (3 × 10 mL) in an ultrasonic cleaner. Evaporation of the solvent and subsequent column chromatography on silica gel afforded the corresponding products **12** (52 mg, 36% yield based on **2**) as a colorless oil, **13** (9.3 mg, 11%) as a white solid. M. p.: 133–135 °C, and **3a** (9.4 mg, 20%) (eluent: *n*-hexane → *n*-hexane/EtOAc = 25:1).

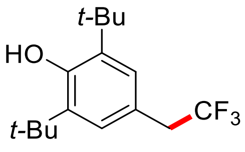

12

2,6-Di-*tert*-butyl-4-(2,2,2-trifluoroethyl)phenol (12)

^{1}H-NMR (400 MHz, $CDCl_3$): δ = 7.04 (s, 1H), 5.20 (s, 1H), 3.25 (q, J = 11.0 Hz, 2H), 1.42 (s, 18H). ^{13}C-NMR (100 MHz, $CDCl_3$): δ = 153.6 (C_q), 136.1 (CH), 126.0 (q, $^1J_{C\text{–}F}$ = 276.9 Hz, C_q), 126.8 (C_q), 120.9 (q, $^3J_{C\text{–}F}$ = 2.9 Hz, C_q), 40.0 (q, $^2J_{C\text{–}F}$ = 29.4 Hz, CH_2), 34.3 (C_q), 30.2 (CH_3). ^{19}F-NMR (376 MHz, $CDCl_3$): δ = −66.2 (s). IR (ATR): 3644, 2955, 2924, 1460, 1436, 1359, 1258, 1134, 1086 cm^{-1}. MS (EI) *m/z* (relative intensity): 288 (30) $[M]^+$, 273 (100) [M-

$CH_3]^+$. HR-MS (EI) *m/z* calc. for $C_{16}H_{23}F_3O$ $[M]^+$: 288.1696, found: 288.1700. The analytical data correspond with those reported in the literature.[6]

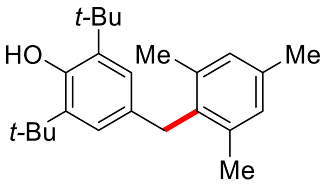

13

2,6-Di-*tert*-butyl-4-(2,4,6-trimethylbenzyl)phenol (13)

^{1}H-NMR (400 MHz, $CDCl_3$): δ = 6.86 (s, 2H), 6.83 (s, 2H), 4.97 (s, 1H), 3.90 (s, 2H), 2.27 (s, 3H), 2.24 (s, 6H), 1.36 (s, 18H). ^{13}C-NMR (100 MHz, $CDCl_3$): δ = 151.6 (C_q), 136.8 (C_q), 135.6 (C_q), 135.2 (C_q), 134.6, (C_q) 130.5 (C_q), 128.8 (CH), 124.4 (CH), 34.5 (CH_2), 34.2 (C_q), 30.3 (CH_3), 20.9 (CH_3), 20.2 (CH_3). IR (ATR): 3645, 2955, 2915, 1614, 1434, 1361, 1232, 1153, 1120, 1026 cm^{-1}. MS (ESI) *m/z* (relative intensity): 337 (100) $[M-H]^+$. HR-MS (ESI) *m/z* calc. for $C_{24}H_{33}O$ $[M-H]^+$: 337.2526, found: 337.2525. The analytical data correspond with those reported in the literature.[7]

Light on/off experiments

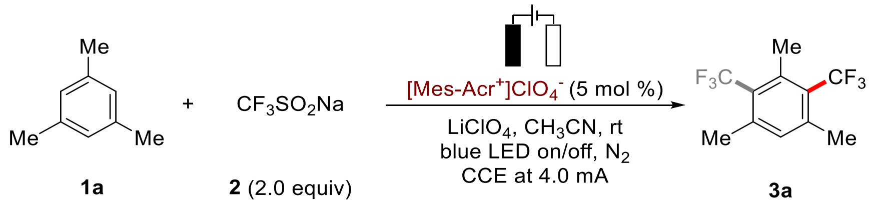

Time (h)	0	1	2	3	4	5	6	7	8	9	10
Light on/off	0	on	off	on	off	on	off	on	off	on	off
Yield (%)	0	23	23	26	26	28	28	29	29	30	30

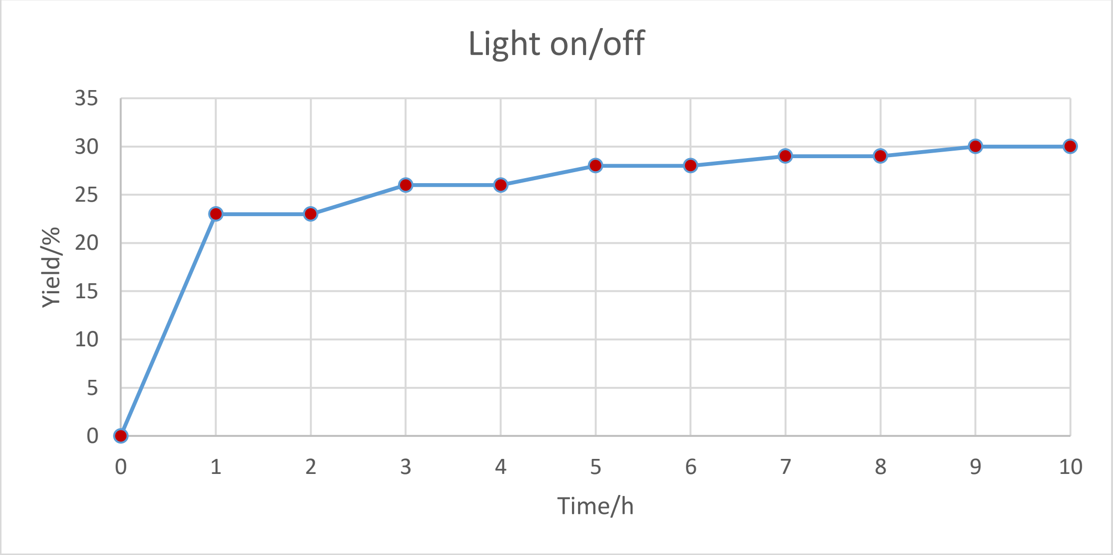

Figure S-7. Light on/off experiments

The electrophotocatalysis was carried out in an undivided cell, with a GF anode (10 mm × 15 mm × 6 mm) and a Pt cathode (10 mm × 15 mm × 0.25 mm). **1a** (30 mg, 0.25 mmol), CF_3SO_2Na (**2**, 78 mg, 0.50 mmol), $LiClO_4$ (42 mg, 0.40 mmol), and [Mes-Acr$^+$]ClO_4^- (5.1 mg, 5.0 mol %) were dissolved in CH_3CN (4.0 mL) under N_2. The electrophotocatalysis was performed at 23 °C with a constant current of 4.0 mA under visible light irradiation (2 × Kessil A360N) at given time intervals. The yield of **3a** was determined by GC/MS analysis of the crude mixture with *n*-dodecane as the internal standard.

Electricity on/off experiments

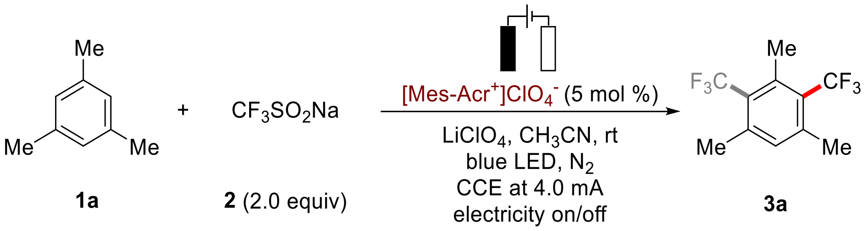

Time (h)	0	1	2	3	4	5	6	7	8	9	10
Electricity on/off	0	on	off	on	off	on	off	on	off	on	off
Yield (%)	0	23	25	36	38	45	46	54	54	64	64

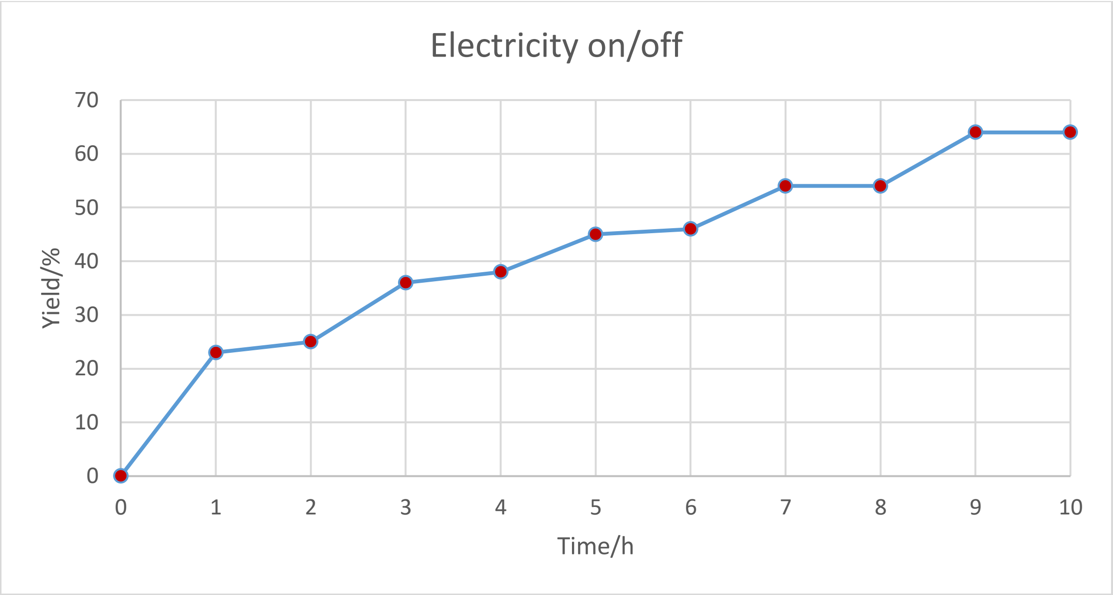

Figure S-8. Electricity on/off experiments

The electrophotocatalysis was carried out in an undivided cell, with a GF anode (10 mm × 15 mm × 6 mm) and a Pt cathode (10 mm × 15 mm × 0.25 mm). **1a** (30 mg, 0.25 mmol), CF_3SO_2Na (**2**, 78 mg, 0.50 mmol), $LiClO_4$ (42 mg, 0.40 mmol), and $[Mes\text{-}Acr^+]ClO_4^-$ (5.1 mg, 5.0 mol %) were dissolved in CH_3CN (4.0 mL) under N_2. The electrophotocatalysis was performed at 23 °C with a constant current of 4.0 mA under visible light irradiation (2 × Kessil A360N) at given time intervals. The yield of **3a** was determined by GC/MS analysis of the crude mixture with *n*-dodecane as the internal standard.

Estimation of quantum yield

The quantum yield *Φ* is defined as the ratio between the number or rate of desired photochemical transformations and the number or rate of absorbed photons. In our reaction, the reaction velocity v_r is driven by electric current, which for a 2-electron process at ideally 100% faradaic yield corresponds to a maximum value of:

$$v_r = \frac{4\,mA}{2\,F} = \frac{0.004\,C\,s^{-1}}{2 \times\ 96485\,C\,mol^{-1}} \leq 2.07 \times 10^{-8}\,mol\,s^{-1}$$

The photon flux was determined using the well-established Hatchard-Parker actinometer.[7] Two solutions were prepared for the quantification of Fe(II) produced by photochemical decomposition of potassium ferrioxalate:

*Ferrioxalate solution **F***: 124 mg (0.25 mmol) of commercially obtained $K_3[Fe(C_2O_4)_3]$ were dissolved in approx. 20 mL of distilled water. Subsequently, 250 mg conc. H_2SO_4 (2.5 mmol) were added and the solution was topped with distilled water to a total volume of 25 mL.

*Phenanthroline buffer **P***: 150 mg (0.83 mmol) phenanthroline and 2.050 g (25 mmol) NaOAc were dissolved in approx. 40 mL of distilled water. Subsequently, 0.50 mLH_2SO_4 (9 mmol) were added and the solution was topped with distilled water to a total volume of 50 mL.

The measurement was performed in the typical reaction setup: A Schlenk-tube, equipped with a magnetic stirring bar and a rubber septum with electrode inlets was charged with 4 mL of solution ***F***. The sample was irradiated with blue light for 20 s (2 × Kessil A360N). Subsequently, a 1/40 aliquot (100 µL) was taken and dissolved in 10 mL of solution ***P*** to obtain *complex solution* $\boldsymbol{C_1}$. This procedure was repeated with a non-irradiated sample to obtain *complex solution* $\boldsymbol{C_0}$.

The quantification of Fe(II) amount relies on the phenanthroline complex, which possesses an absorptivity *ε* of 11000 $L \cdot mol^{-1} \cdot cm^{-1}$ at 510 nm. UV absorbance *A* of $\boldsymbol{C_0}$ was recorded as a blank. The absorbance of $\boldsymbol{C_1}$ at 510 nm was 0.58. According to the Lambert-Beer law, this corresponds at an optical path length of *l* = 1 cm to a concentration of:

$$c = \frac{A}{\varepsilon\,l} = \frac{0.58}{11000\,L\,mol^{-1}\,cm^{-1}\ \times 1\,cm} = 5.3 \times 10^{-5}\,mol\,L^{-1}$$

Given a dilution factor of 100 and a quantum yield of 0.86 of potassium ferrioxalate at 436 nm, the total amount of photons absorbed per second v_p in the sample is:

$$v_p = \frac{5.3 \times 10^{-5} mol\, L^{-1} \times 100 \times 0.004\, L}{0.86 \times 20\, s} = 1.23 \times 10^{-6}\, mol\, s^{-1}$$

The ratio between v_r and v_p is the estimated quantum yield:

$$\Phi = \frac{v_r}{v_p} = \frac{2.07 \times 10^{-8}\, mol\, s^{-1}}{1.23 \times 10^{-6}\, mol\, s^{-1}} \leq 1.7 \times 10^{-2}\ (1.7\%)$$

Fluorescence Quenching Experiments

Sample solutions were prepared in in a MeCN/H_2O 9:1 mixture with concentrations of 10^{-4} M for [Mes-Acr$^+$]ClO_4^-, 10^{-5} M for $[Ru(bpy)_3](PF6)_2$ and varying concentrations of quencher (**1a** or **2**). The sample solutions were degassed prior to measurement by N_2-bubbling. Stern-Volmer experiments were conducted with fixed excitation wavelengths of 450 nm for $[Ru(bpy)_3](PF_6)_2$ and 400 nm for [Mes-Acr$^+$]ClO_4^- and detection at the emission maximum of the respective analyte. Plotting of the I_0/I value against the concentration of the potential quencher yielded the following graphs.

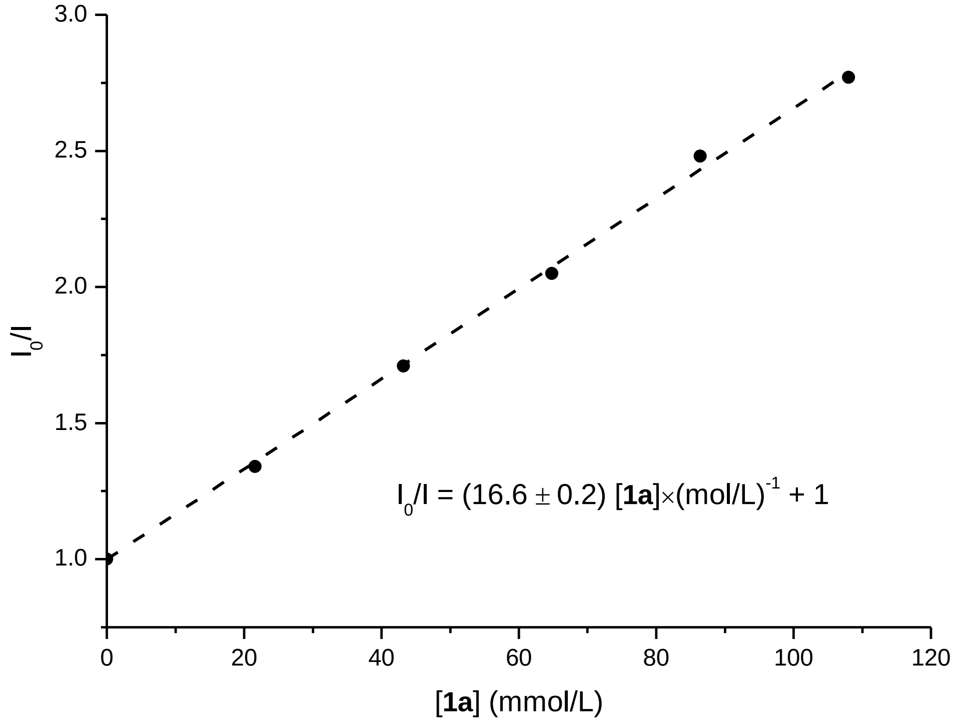

Figure S-9. Quenching of [Mes-Acr$^+$]ClO_4^- with **1a**:

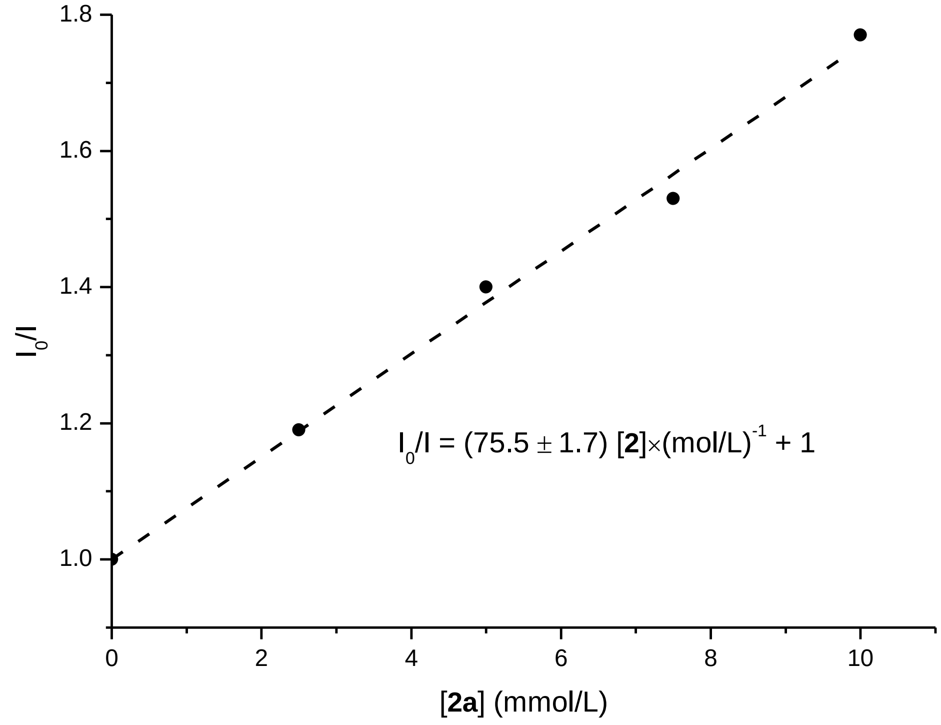

Figure S-10. Quenching of [Mes-Acr$^+$]ClO_4^- with **2**:

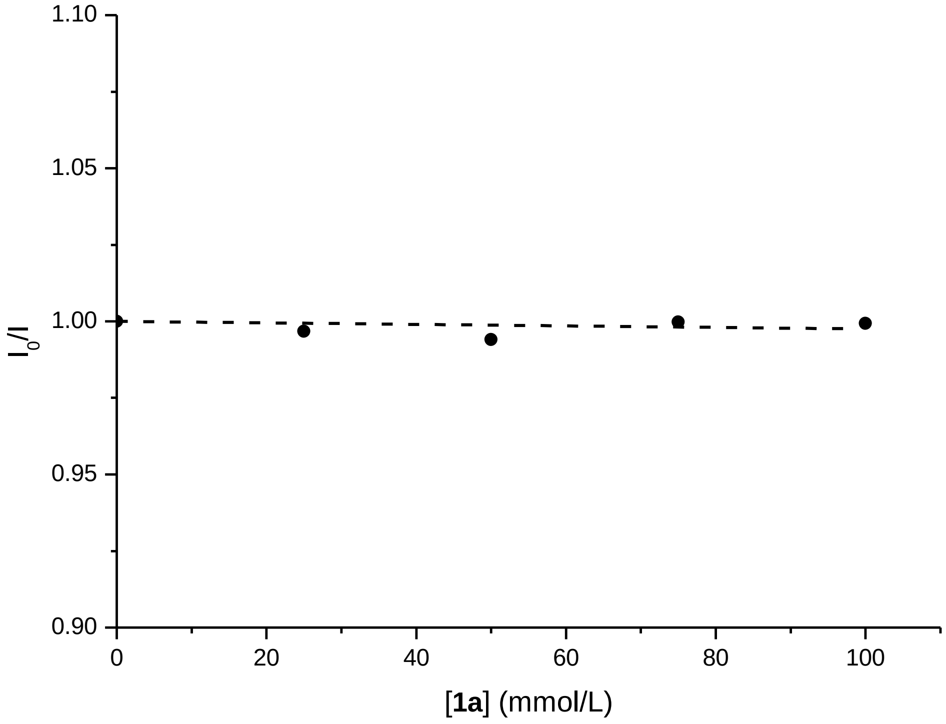

Figure S-11. Quenching of $[Ru(bpy)_3](PF_6)_2$ with **1a**:

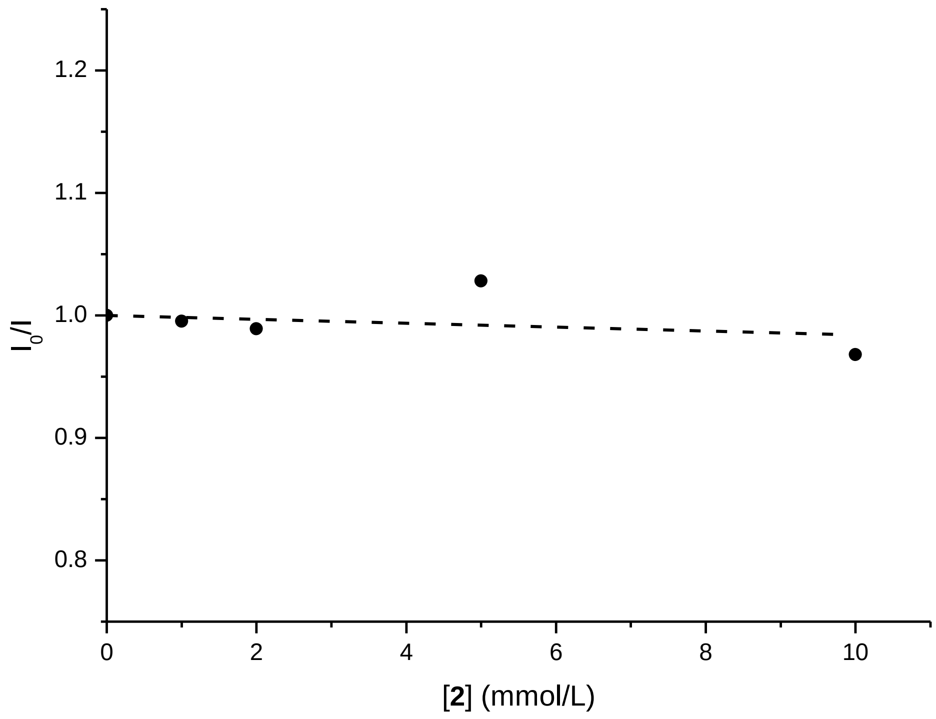

Figure S-12. Quenching of $[Ru(bpy)_3](PF_6)_2$ with **2**:

GC-Headspace Detection of H_2

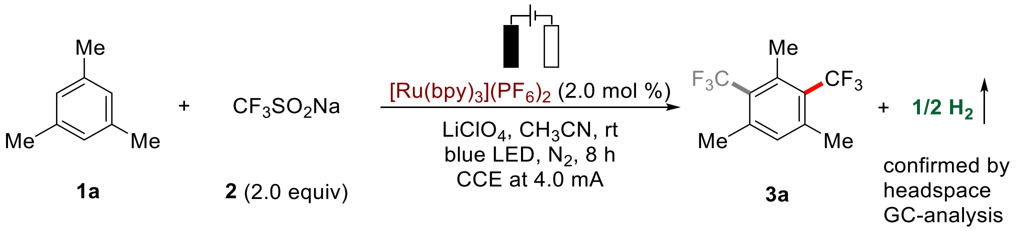

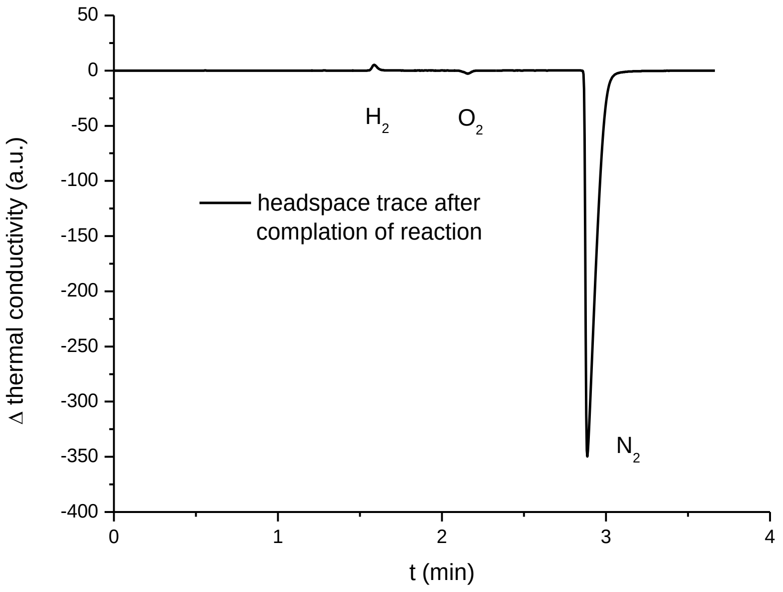

In a Schlenk tube equipped with GF anode (10 mm × 10 mm × 6 mm) and a platinum cathode (20 mm × 10 mm × 0.25 mm), **1a** (30 mg, 0.25 mmol), CF_3SO_2Na **2** (78 mg, 0.50 mmol), $LiClO_4$ (42 mg, 0.40 mmol) and $[Ru(bpy)_3](PF_6)_2$ (4.3 mg, 2.0 mol %) were dissolved in CH_3CN (4.0 mL). The atmosphere was exchanged to N_2 and the stopcock has been closed. The electrophotocatalysis was performed at 23 °C with a constant current of 4.0 mA under visible light irradiation (2 × Kessil A360N). After 8 h, 1.0 mL of the headspace volume was taken for GC analysis.

Cyclic Voltammetry

The cyclic voltammetry was carried out with a Metrohm Autolab PGSTAT204 potentiostat and Nova 2.1 software. For all experiments, a glassy carbon working electrode (disk, diameter: 3 mm), a platinum wire counter electrode and a saturated calomel reference electrode (SCE) were employed. The voltammograms were recorded in MeCN at a substrate concentration of 5.0 mmol/L and with 0.1 mol/L $LiClO_4$ as supporting electrolyte. All solutions were saturated with nitrogen gas prior to measurement. The scan rate was 100 mV/s. Blue light irradiation was accomplished by one Kessil A360N lamp, mounted in 10 cm distance from the electrochemical cell, turned up to highest intensity and lowest wave length.

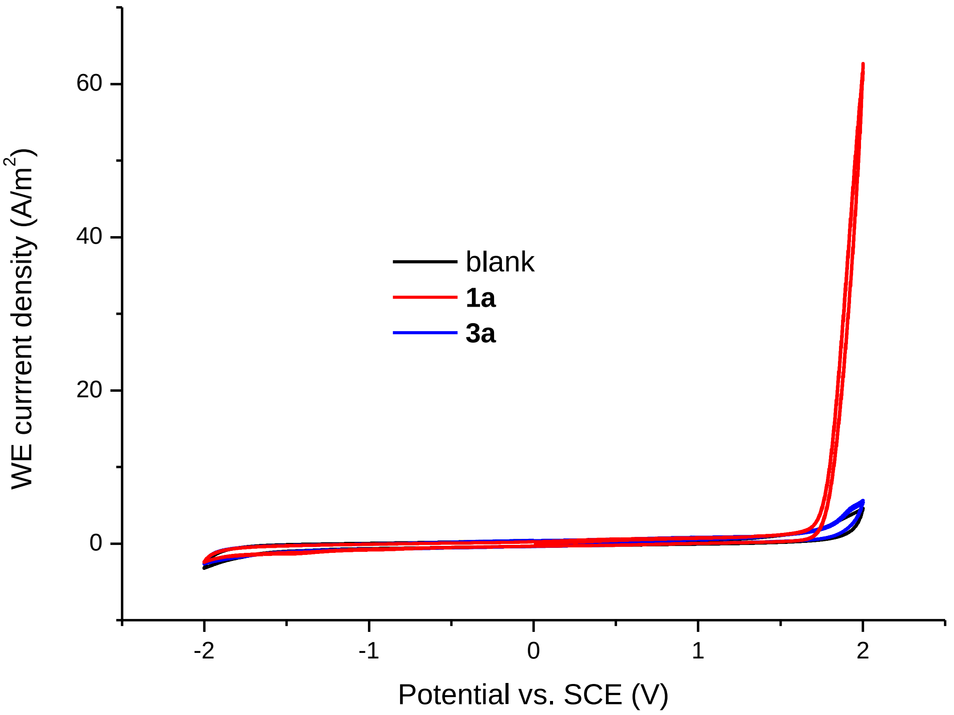

Figure S-13. Cyclic voltammograms at 100 mVs^{-1}, substrates (5 mmol/L) and $LiClO_4$ (100 mmol/L) in MeCN: blank (black), **1a** (red), **3a** (blue).

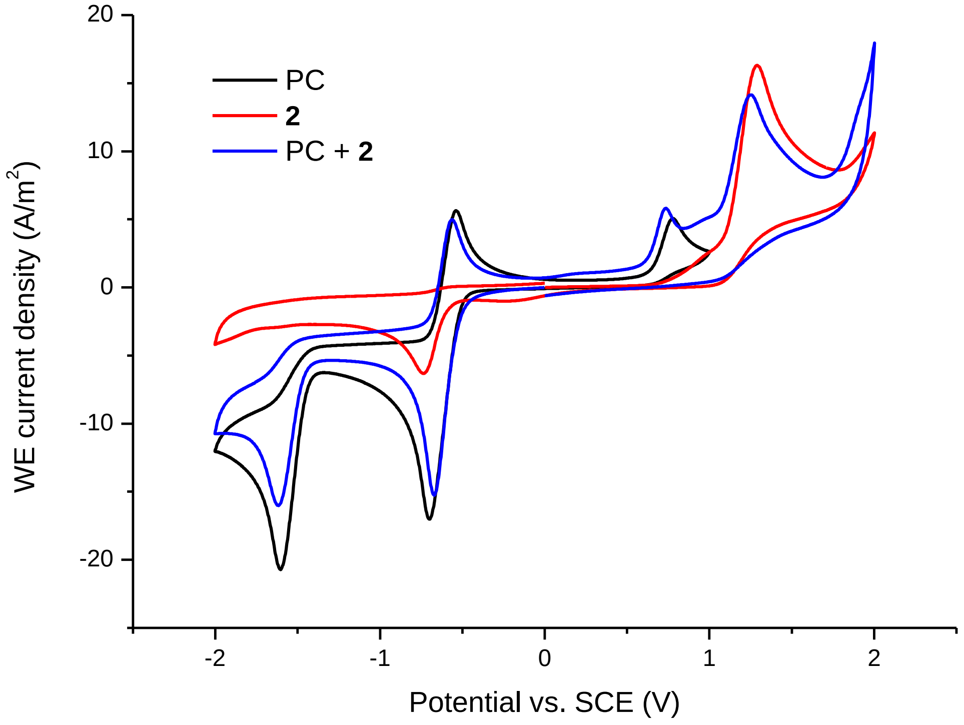

Figure S-14. Cyclic voltammograms at 100 mVs^{-1}, substrates (5 mmol/L) and $LiClO_4$ (100 mmol/L) in MeCN: PC (black), **2** (red), PC + **2** (blue).

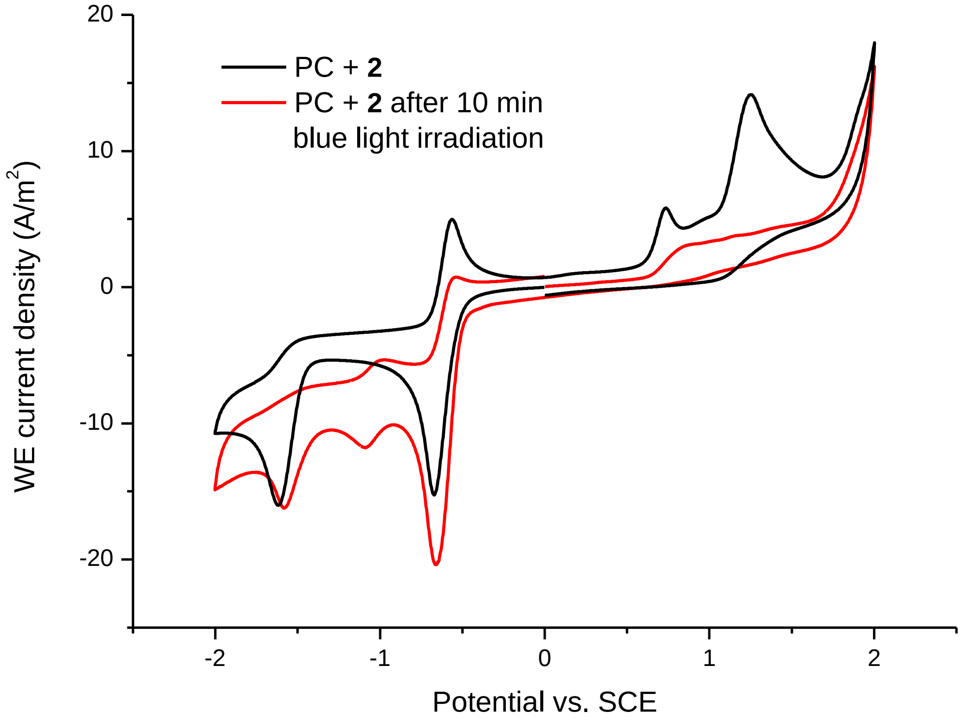

Figure S-15. Cyclic voltammograms at 100 mVs^{-1}, substrates (5.0 mmol/L) and $LiClO_4$ (100 mmol/L) in MeCN: PC + **2** (black), PC + **2** after being irradiated for 10 minutes with blue light (red).

Plausible mechanism with $[Ru(bpy)_3](PF_6)_2$ as catalyst

Scheme S-1. Plausible mechanism

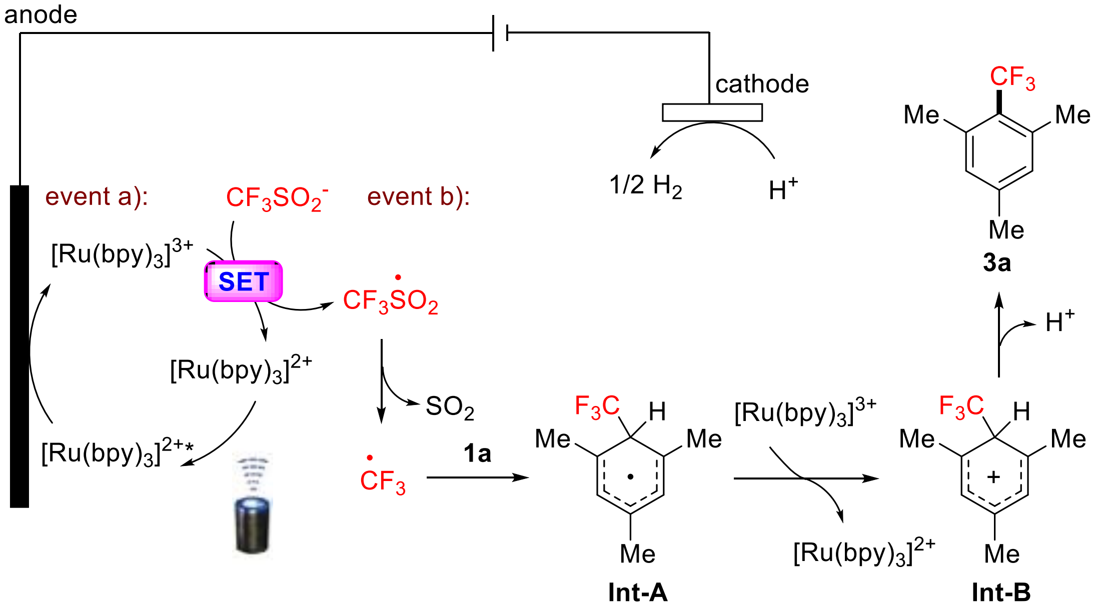

References

[1] Y. Ouyang, X.-H. Xu, F.-L. Qing, *Angew. Chem. Int. Ed.* **2018**, *57*, 6926–6929.

[2] K. Natte, R. V. Jagadeesh, L. He, J. Rabeah, J. Chen, C. Taeschler, S. Ellinger, F. Zaragoza, H. Neumann, A. Brückner, M. Beller, *Angew. Chem. Int. Ed.* **2016**, *55*, 2782–2786.

[3] Y. Deng, F. Lu, S. You, T. Xia, Y. Zheng, C. Lu, G. Yang, Z. Chen, M. Gao, A. Lei, *Chin. J. Chem.* **2019**, *37*, 817–820.

[4] J. Lin, Z. Li, J. Kan, S. Huang, W. Su, Y. Li, *Nat. Commun.* **2017**, *8*, 14353.

[5] W.-J. Kong, L. H. Finger, A. M. Messinis, R. Kuniyil, J. C. A. Oliveira, L. Ackermann, *J. Am. Chem. Soc.* **2019**, *141*, 17198–17206.

[5] J. Wang, K. Sun, X. Chen, T. Chen, Y. Liu, L. Qu, Y. Zhao, B. Yu, *Org. Lett.* **2019**, *21*, 1863–1867.

[6] I. G. Arzamanova, I. P. Romm, Y. K. Tovbin, E. N. Gur'yanova, E. A. Gurvich, A. I. Rybak, *Zh. Org. Khim.* **1979**, *49*, 672–675.

[7] a) C. A. Parker, *Proc. R. Soc. Lond. A* **1953**, *220*, 104–116; b) C. G. Hatchard, C. A. Parker, *Proc. R. Soc. Lond. A* **1956**, *235*, 518–536.

^{1}H- , ^{13}C- and ^{19}F-NMR Spectra

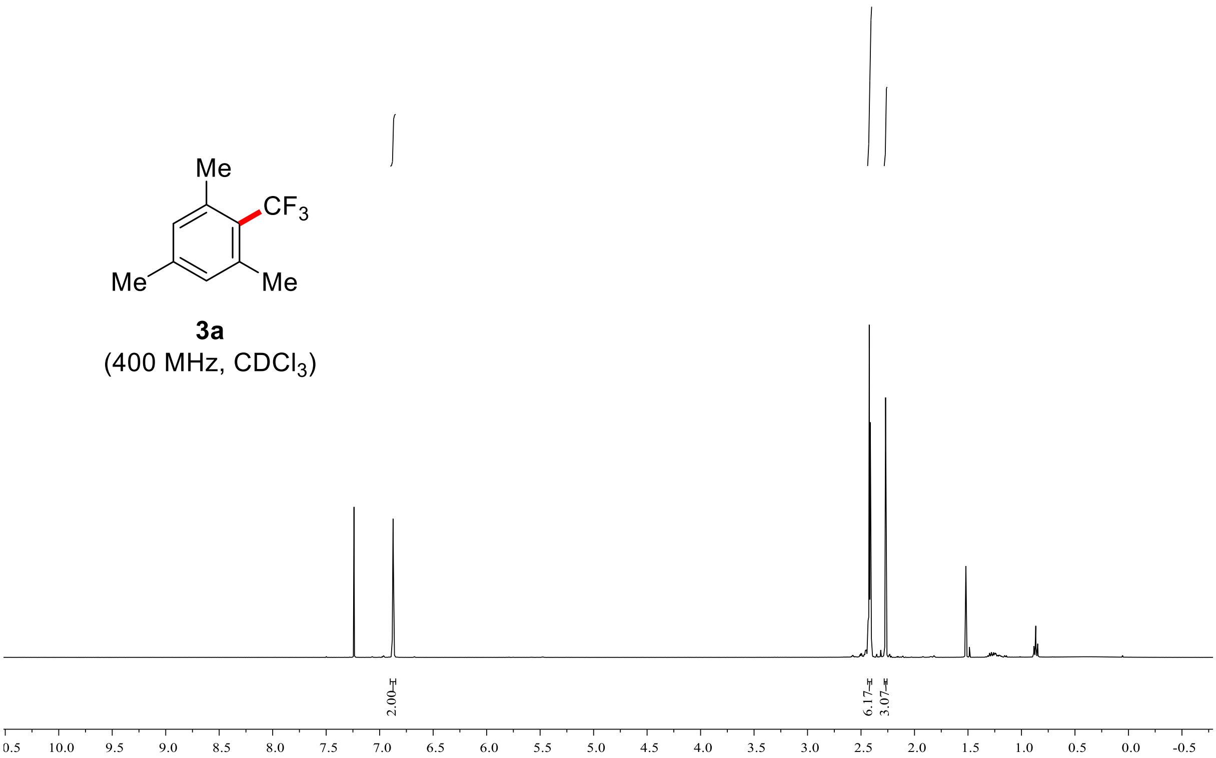

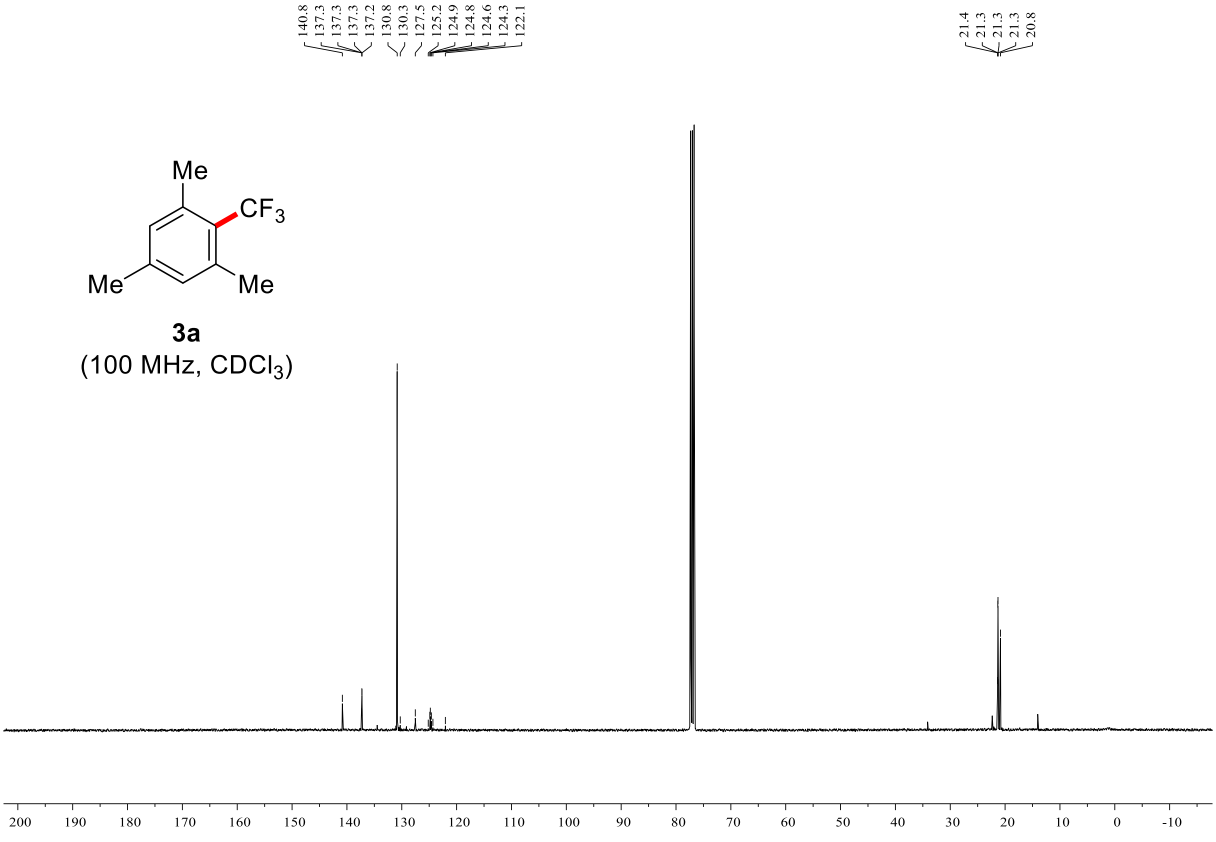

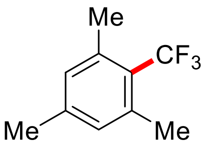

3a
(376 MHz, $CDCl_3$)

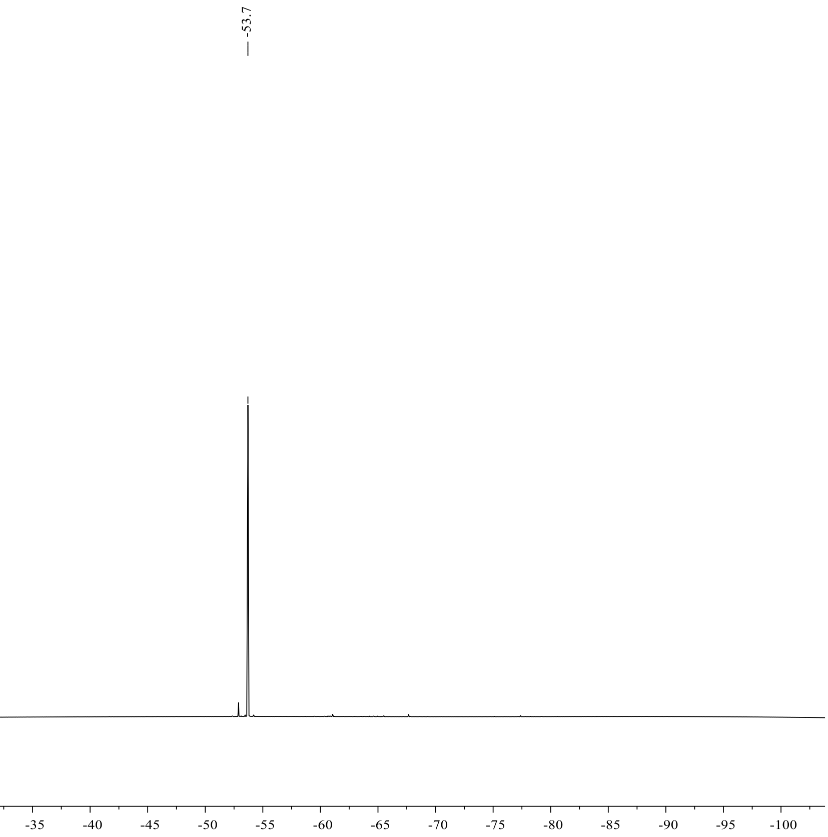

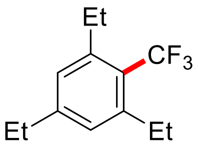

3b
(300 MHz, $CDCl_3$)

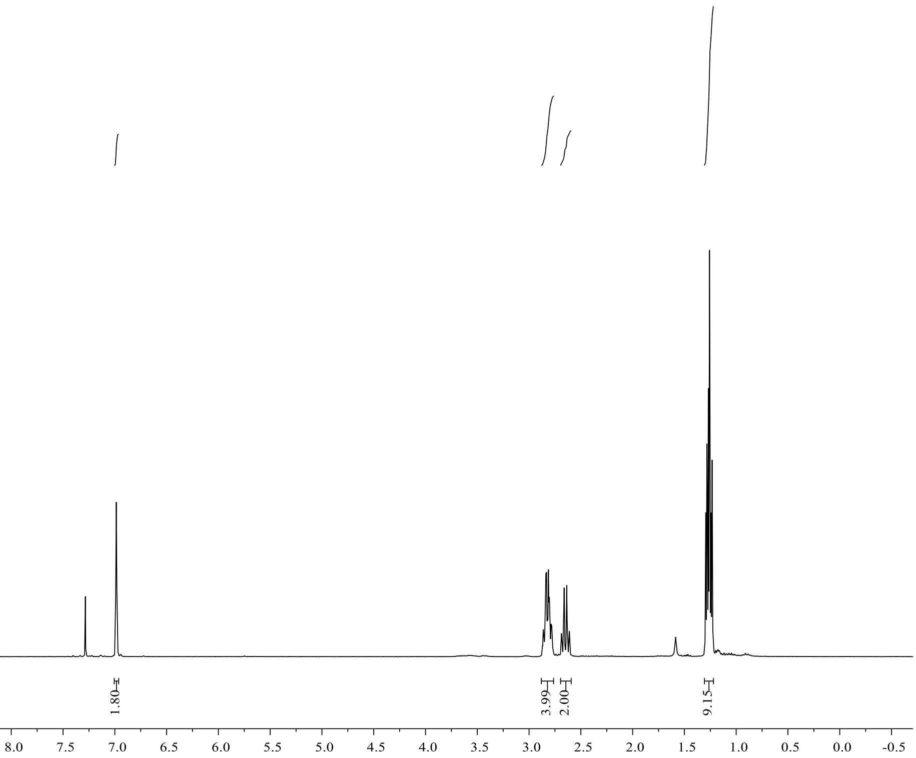

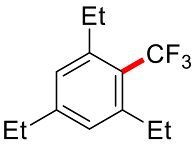

3b
(100 MHz, $CDCl_3$)

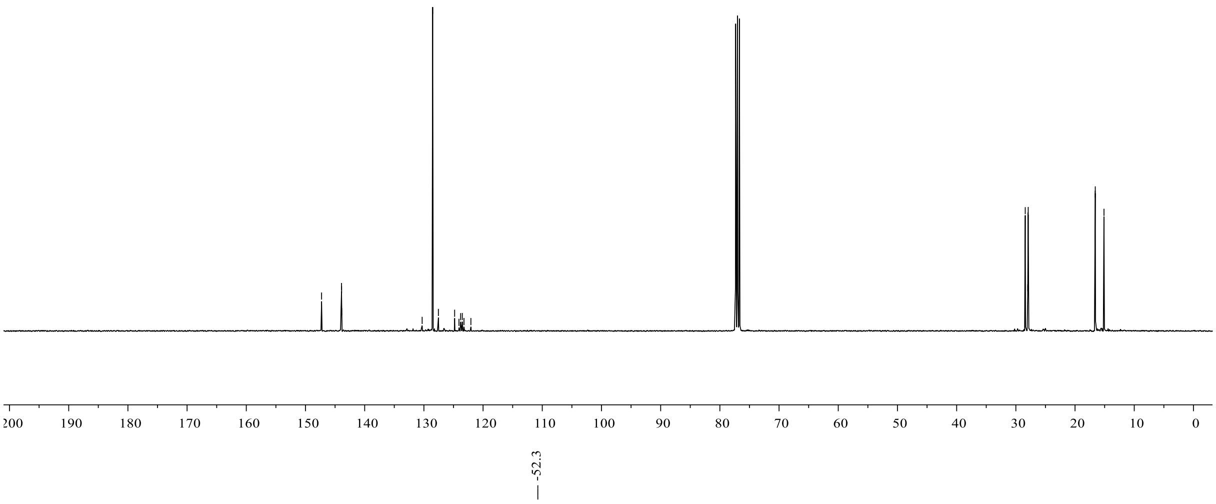

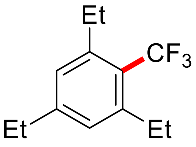

3b
(282 MHz, $CDCl_3$)

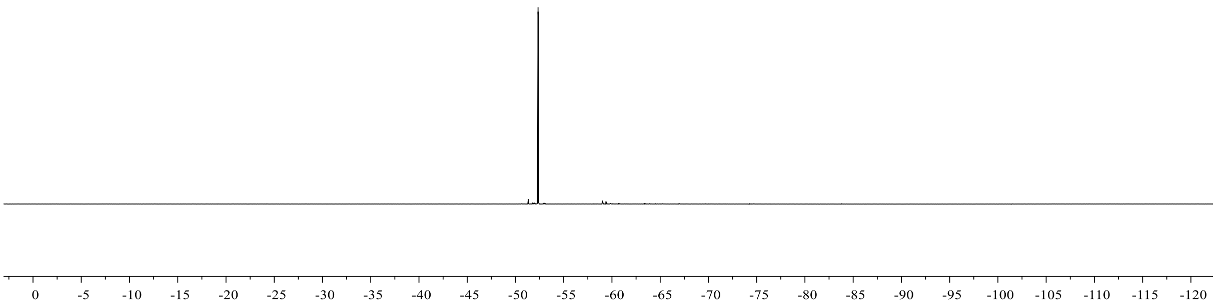

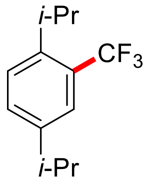

3c
(400 MHz, $CDCl_3$)

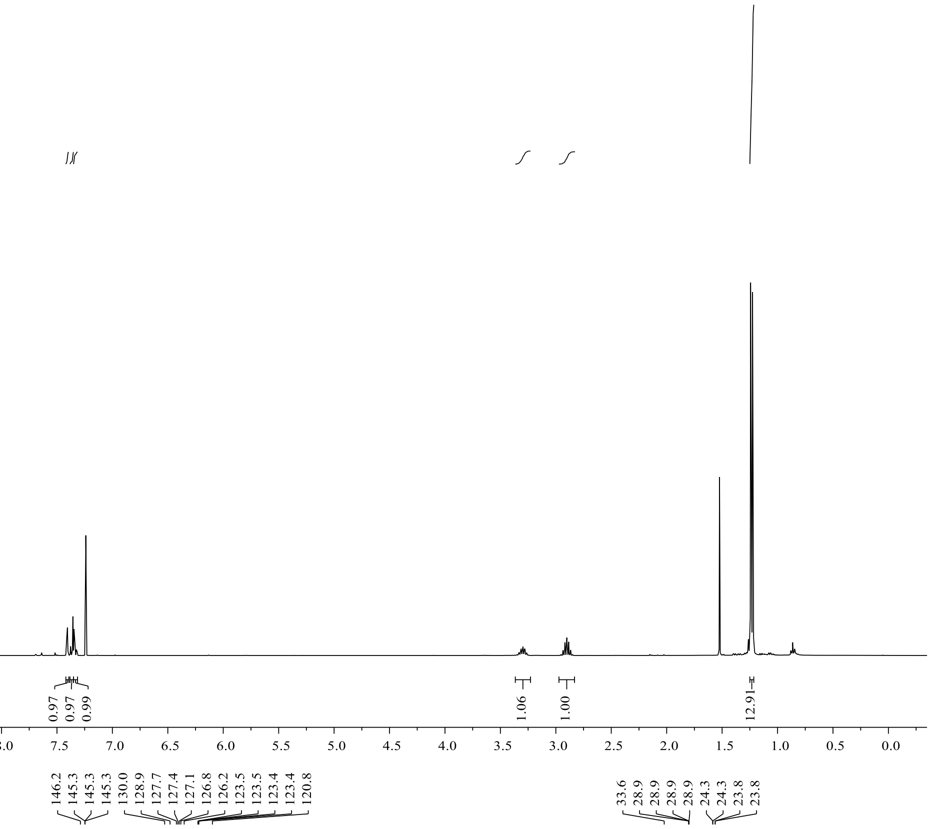

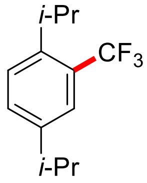

3c
(100 MHz, $CDCl_3$)

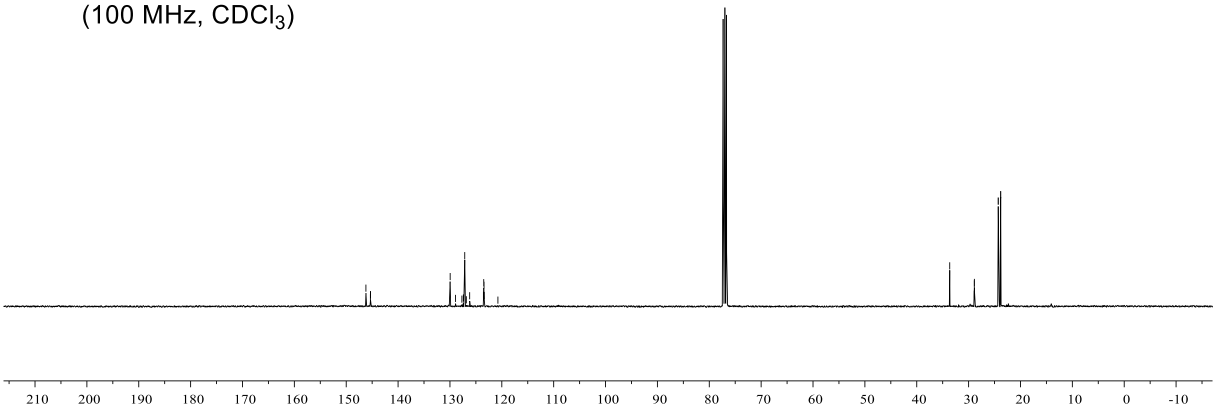

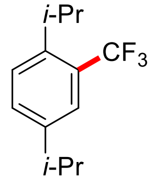

3c
(376 MHz, $CDCl_3$)

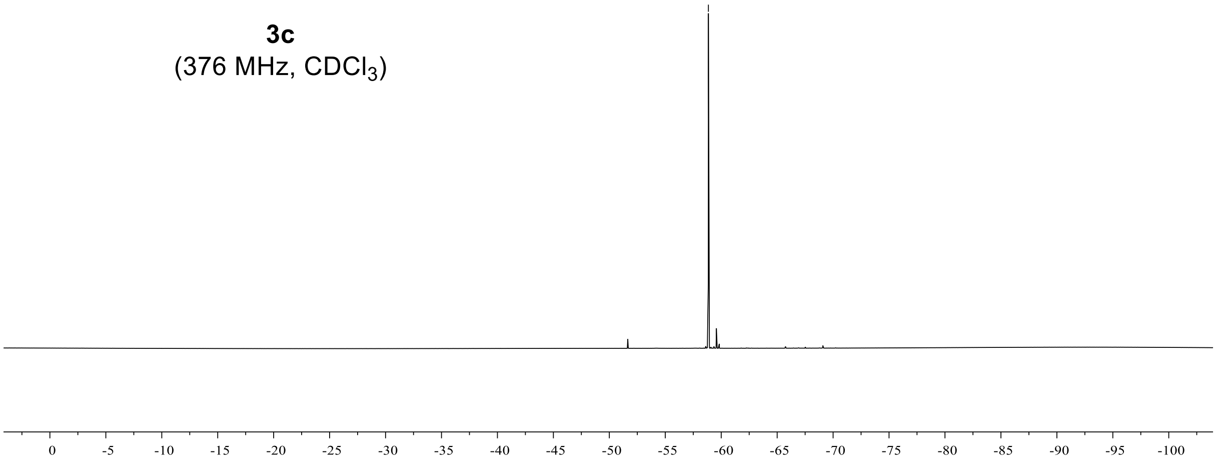

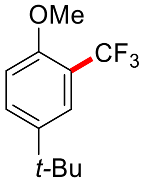

3d
(500 MHz, $CDCl_3$)

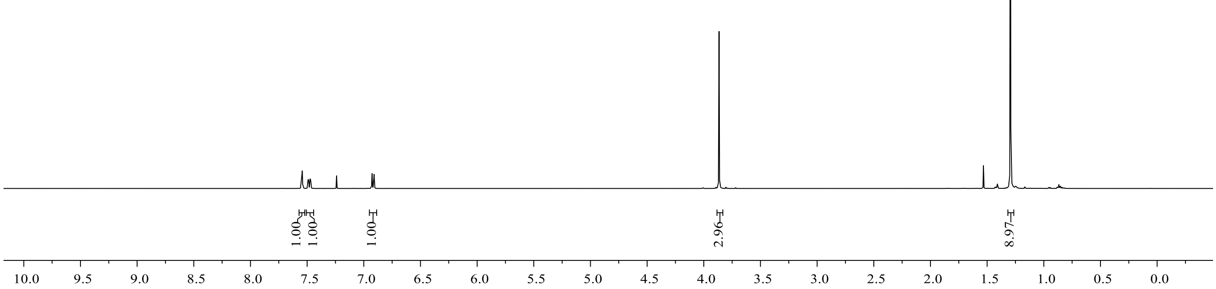

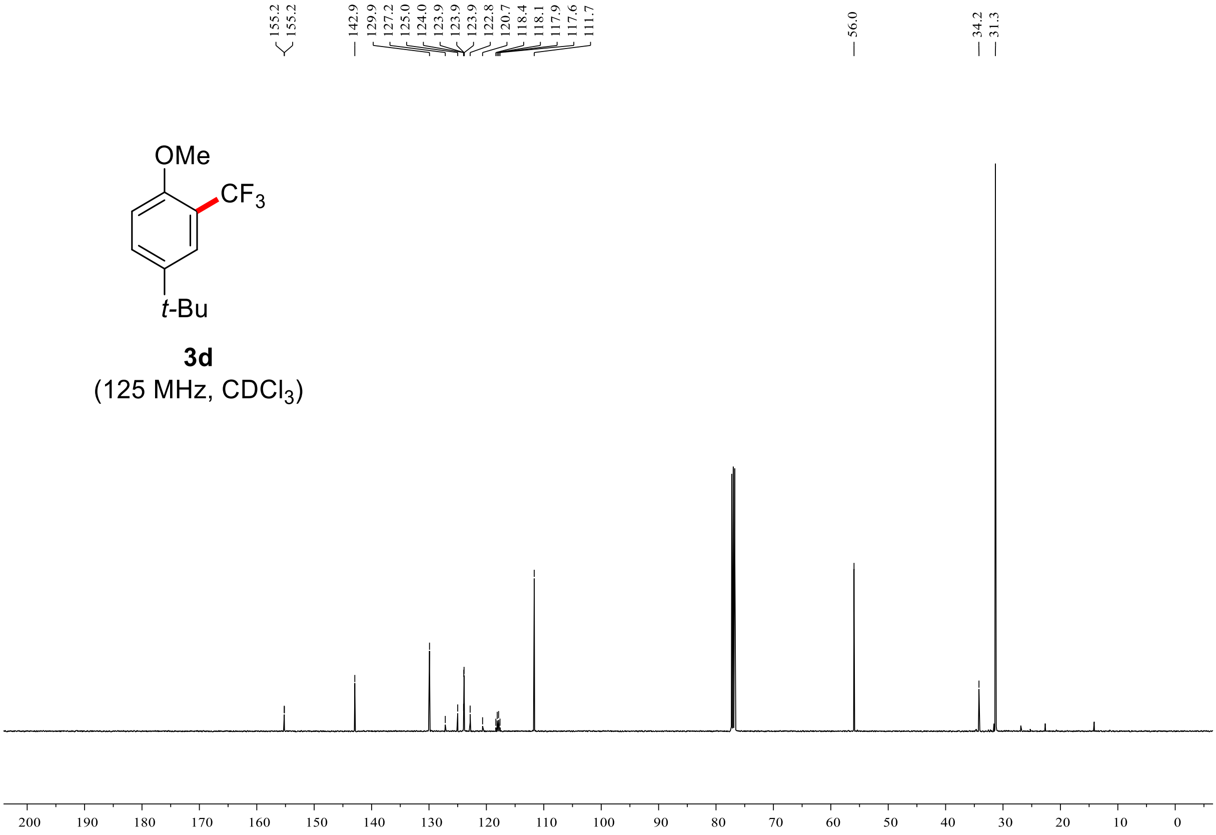

3d
(125 MHz, $CDCl_3$)

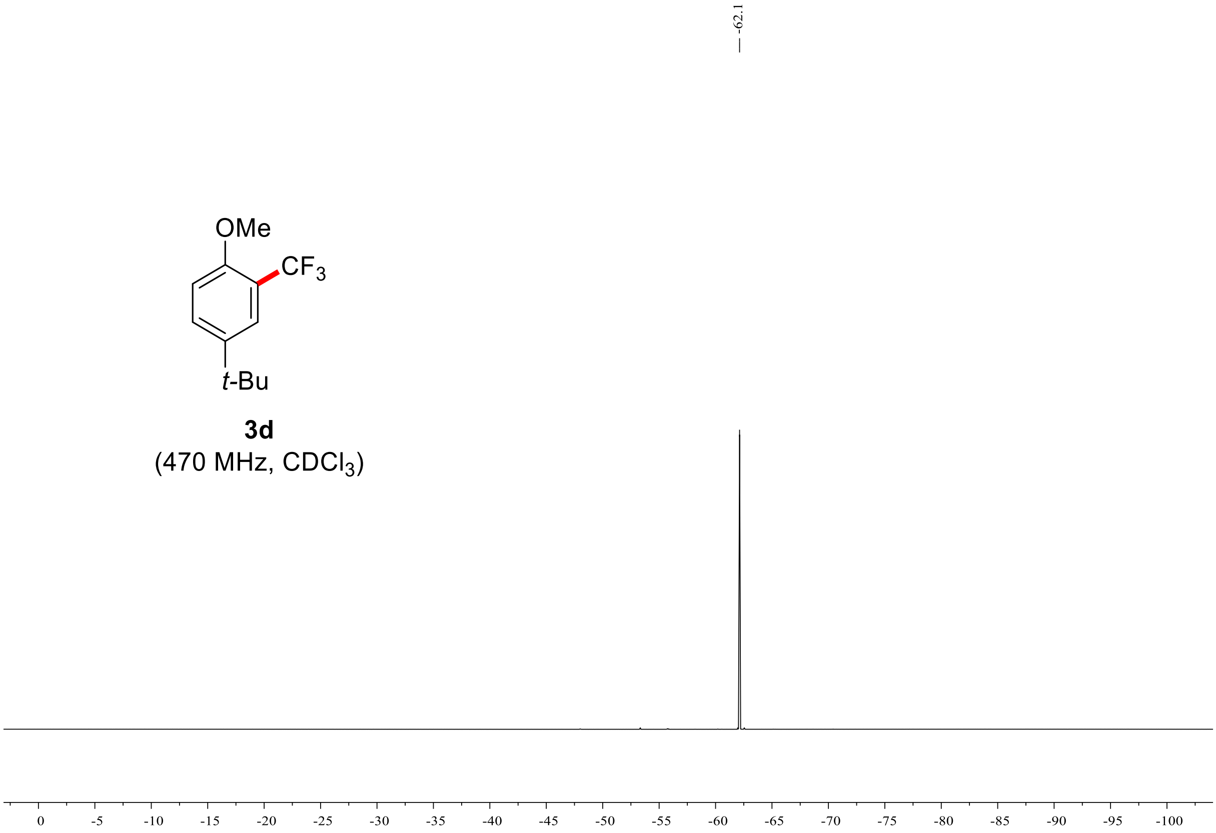

3d
(470 MHz, $CDCl_3$)

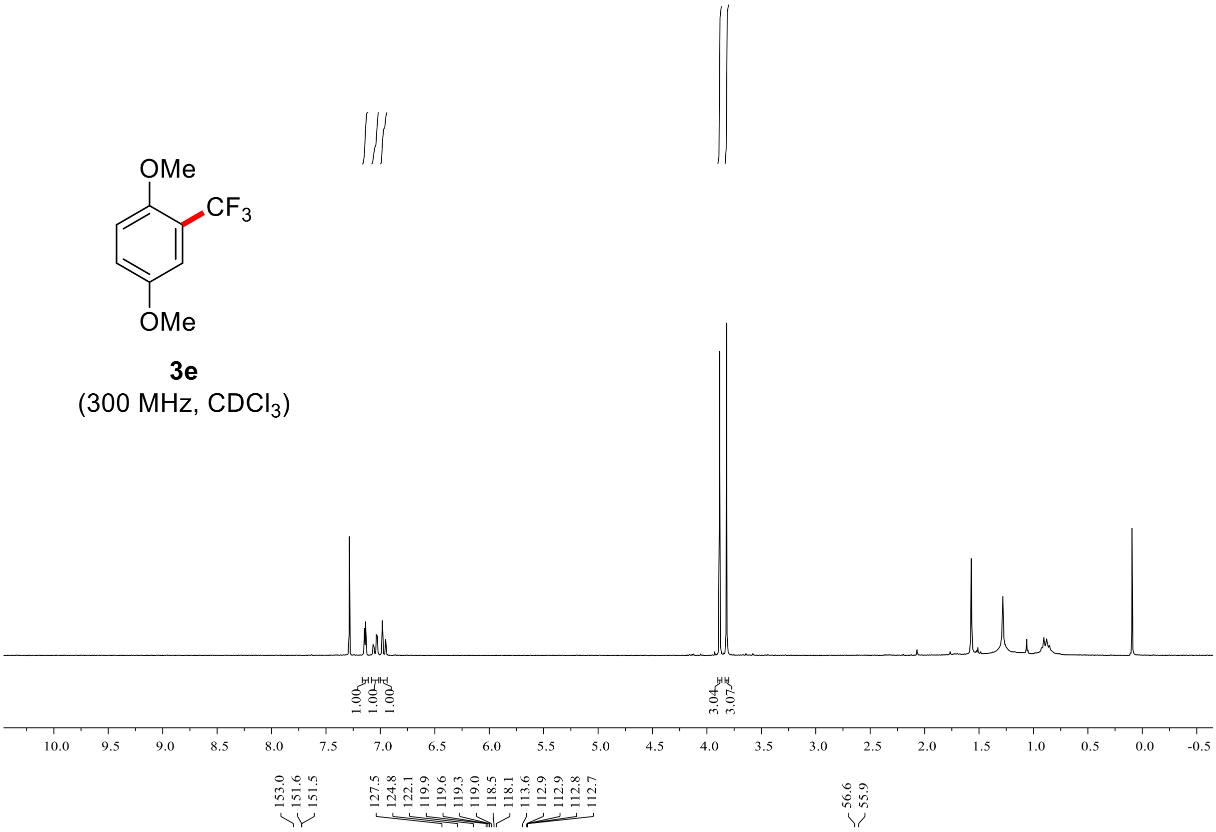
OMe
CF3
OMe
3e
(300 MHz, CDCl3)
1.00
1.00
1.00
3.04
3.07
10.0 9.5 9.0 8.5 8.0 7.5 7.0 6.5 6.0 5.5 5.0 4.5 4.0 3.5 3.0 2.5 2.0 1.5 1.0 0.5 0.0 -0.5
153.0
151.6
151.5
127.5
124.8
122.1
119.9
119.6
119.3
119.0
118.5
118.1
113.6
112.9
112.9
112.8
112.7
56.6
55.9

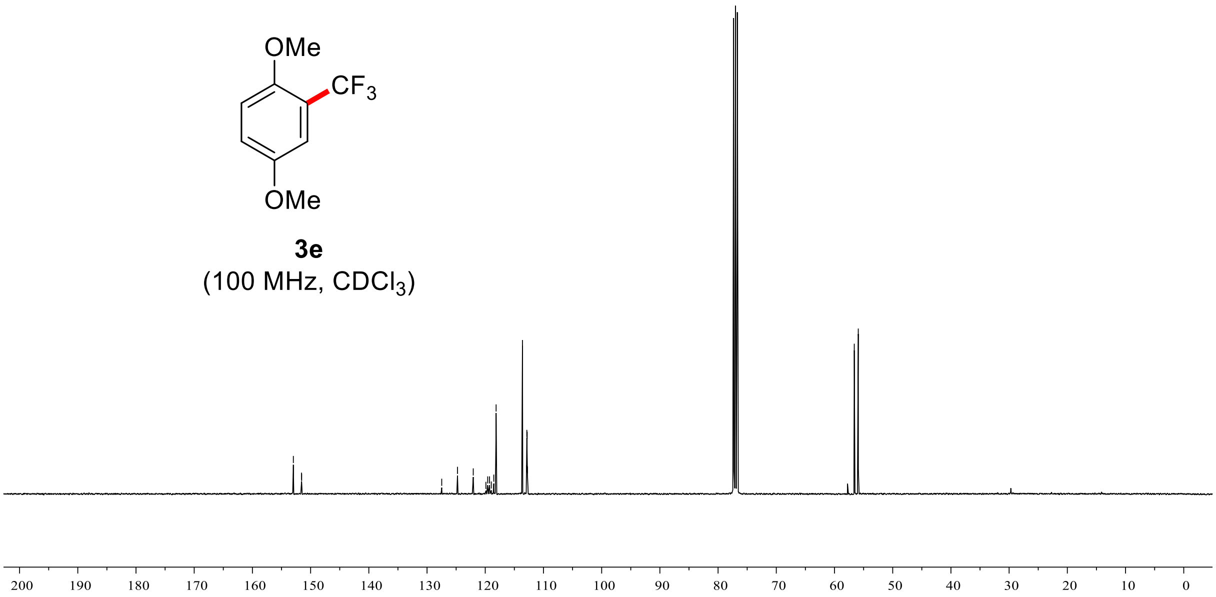
OMe
CF3
OMe
3e
(100 MHz, CDCl3)
200 190 180 170 160 150 140 130 120 110 100 90 80 70 60 50 40 30 20 10 0

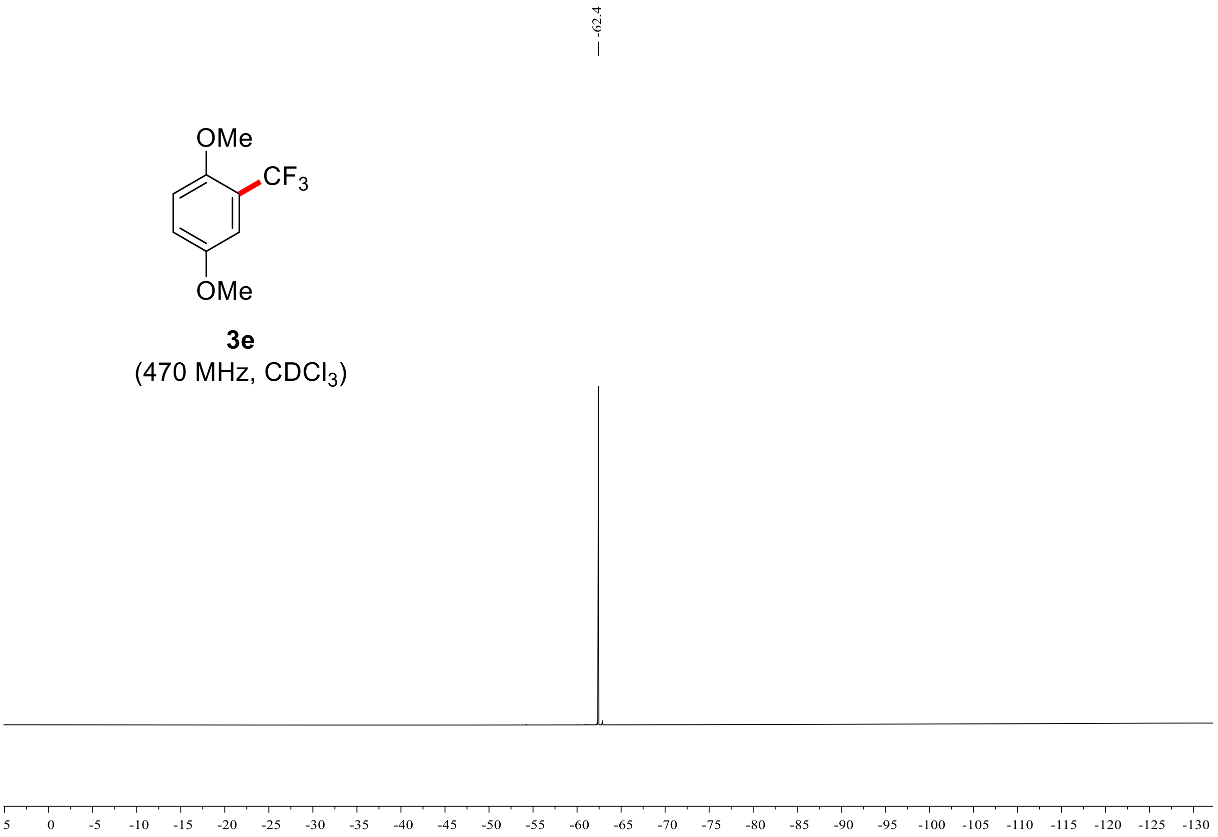
-62.4
OMe
CF3
OMe
3e
(470 MHz, CDCl3)
5 0 -5 -10 -15 -20 -25 -30 -35 -40 -45 -50 -55 -60 -65 -70 -75 -80 -85 -90 -95 -100 -105 -110 -115 -120 -125 -130

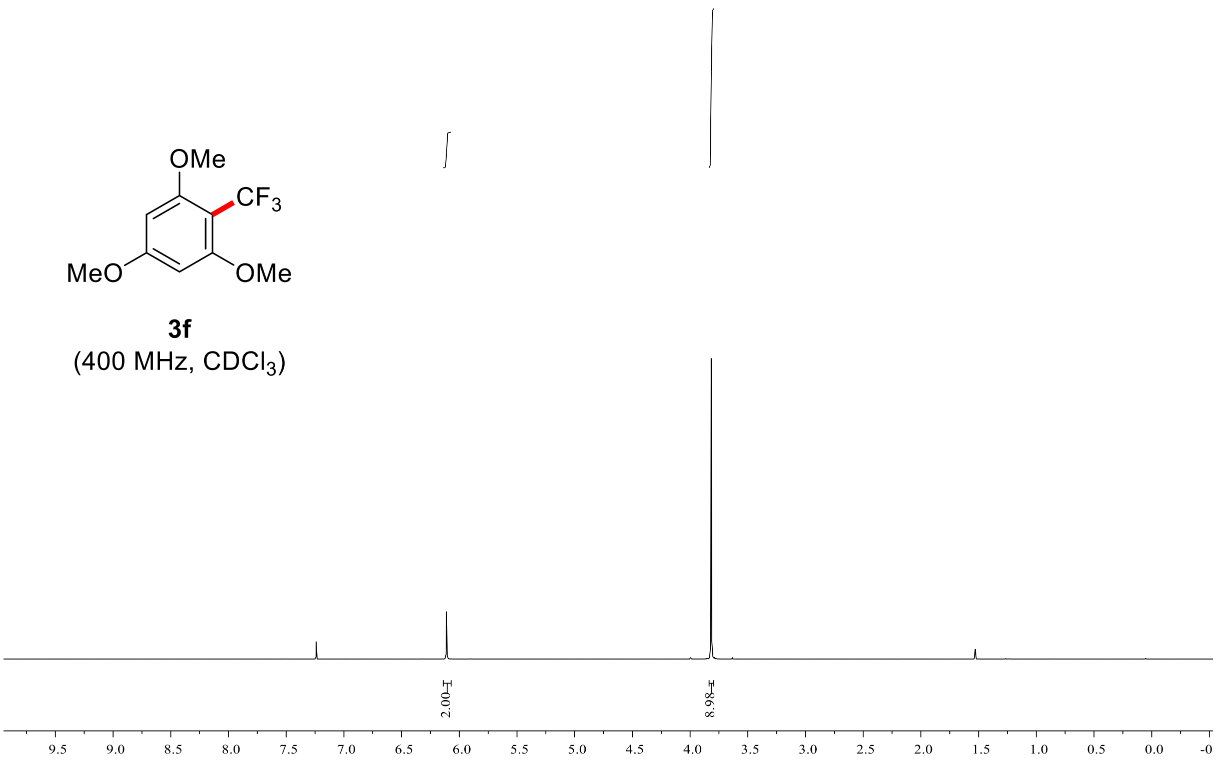
OMe
CF3
MeO
OMe
3f
(400 MHz, CDCl3)
2.00
8.98
9.5 9.0 8.5 8.0 7.5 7.0 6.5 6.0 5.5 5.0 4.5 4.0 3.5 3.0 2.5 2.0 1.5 1.0 0.5 0.0 -0

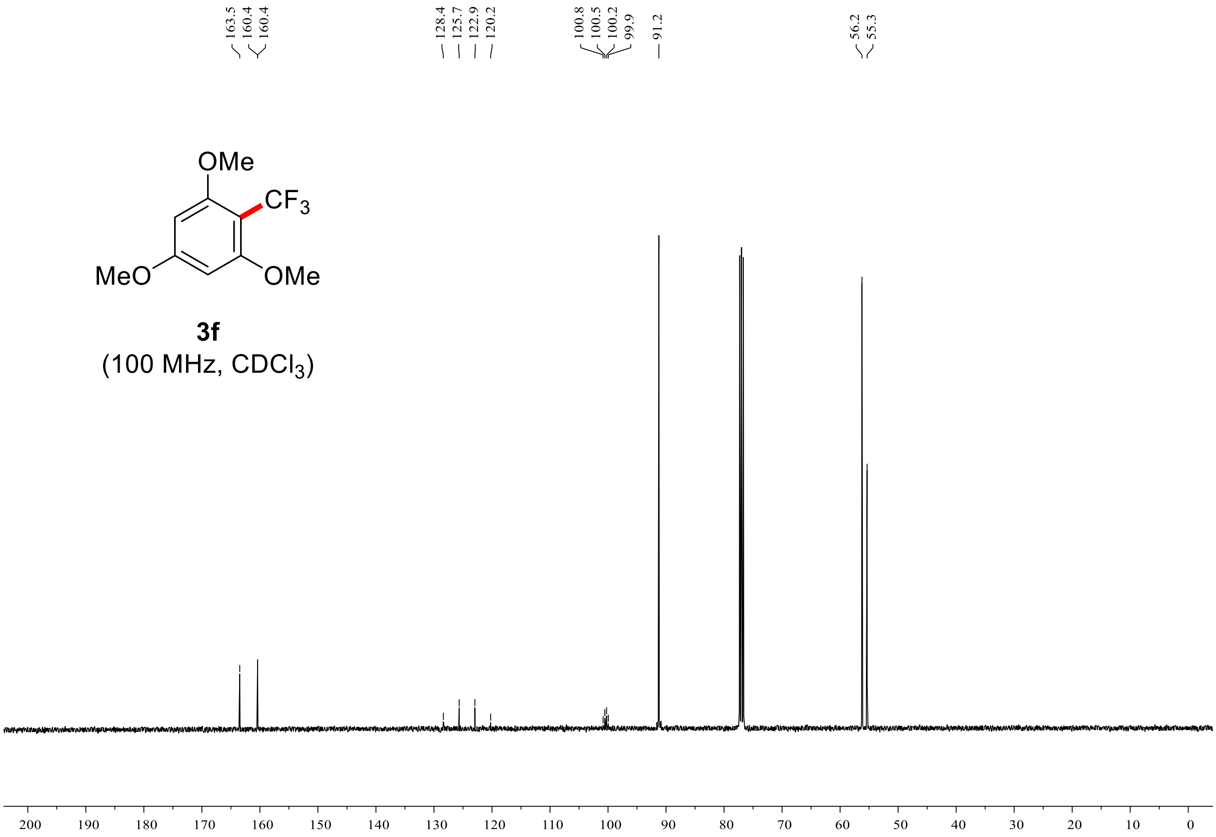
163.5
160.4
160.4
128.4
125.7
122.9
120.2
100.8
100.5
100.2
99.9
91.2
56.2
55.3
OMe
CF3
MeO
OMe
3f
(100 MHz, CDCl3)
200 190 180 170 160 150 140 130 120 110 100 90 80 70 60 50 40 30 20 10 0

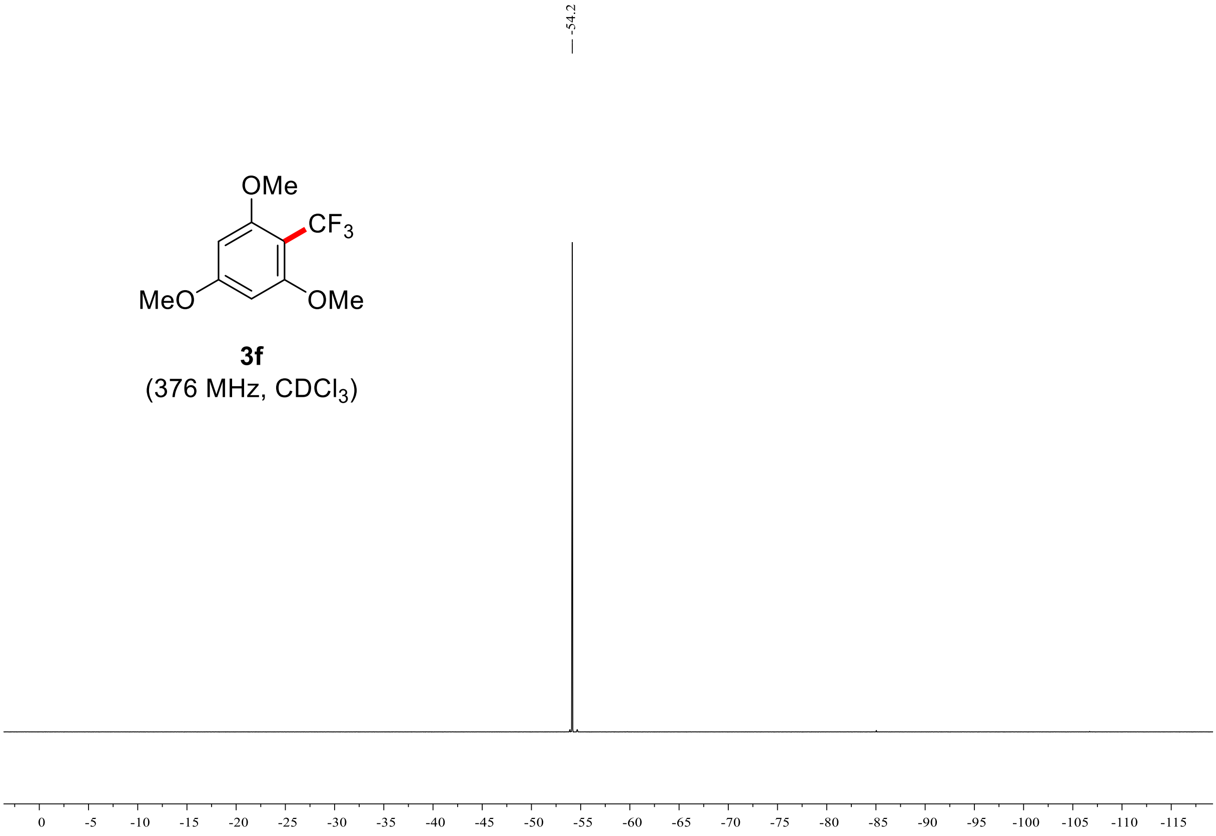
-54.2
OMe
CF3
MeO
OMe
3f
(376 MHz, CDCl3)
0 -5 -10 -15 -20 -25 -30 -35 -40 -45 -50 -55 -60 -65 -70 -75 -80 -85 -90 -95 -100 -105 -110 -115

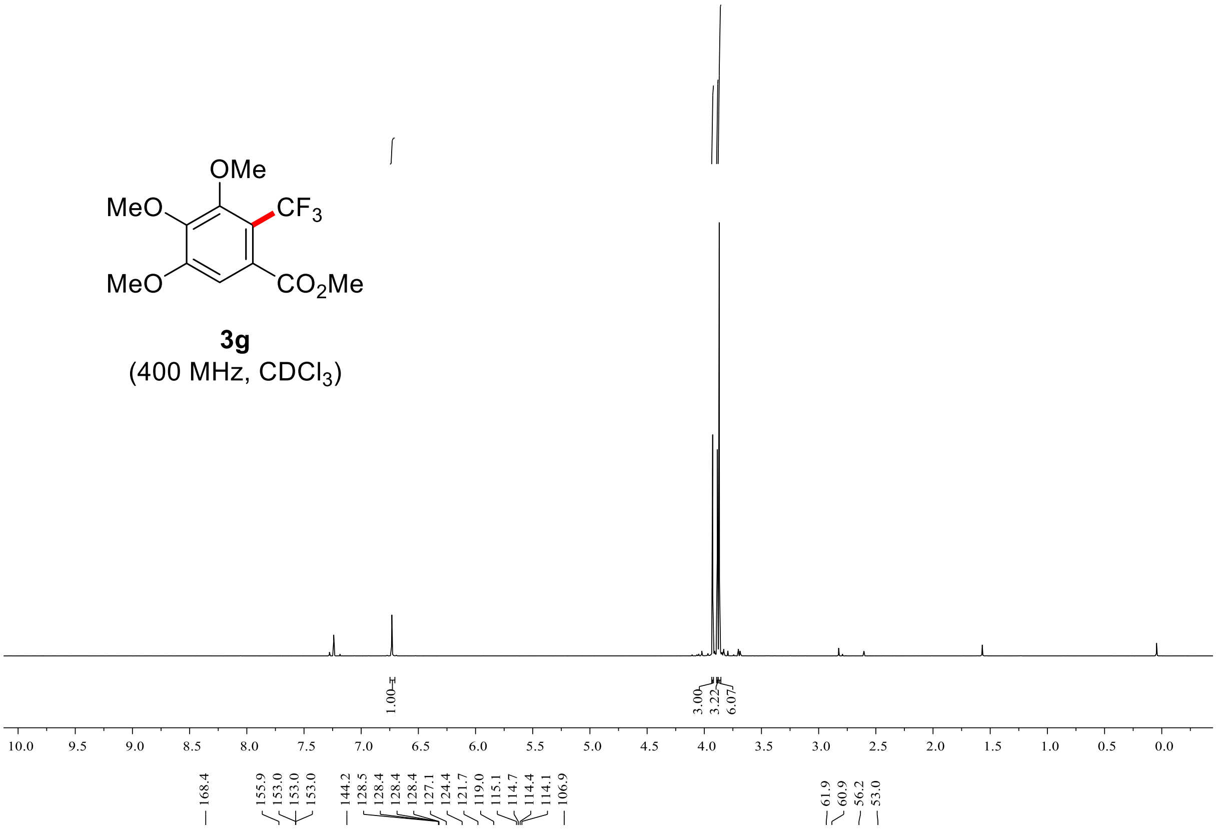

3g
(400 MHz, $CDCl_3$)

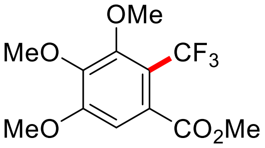

3g
(100 MHz, $CDCl_3$)

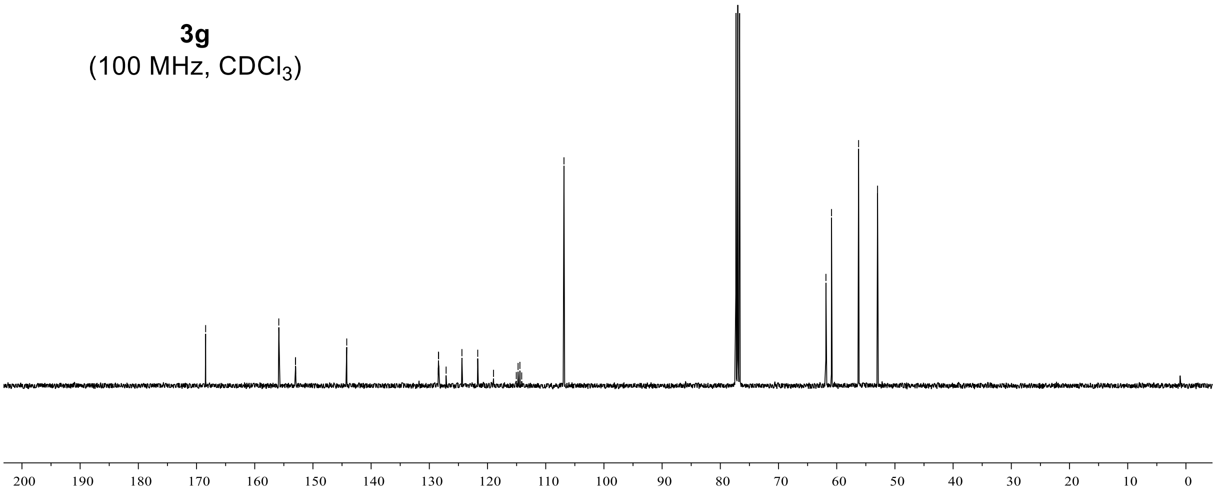

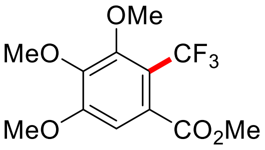

3g
(376 MHz, $CDCl_3$)

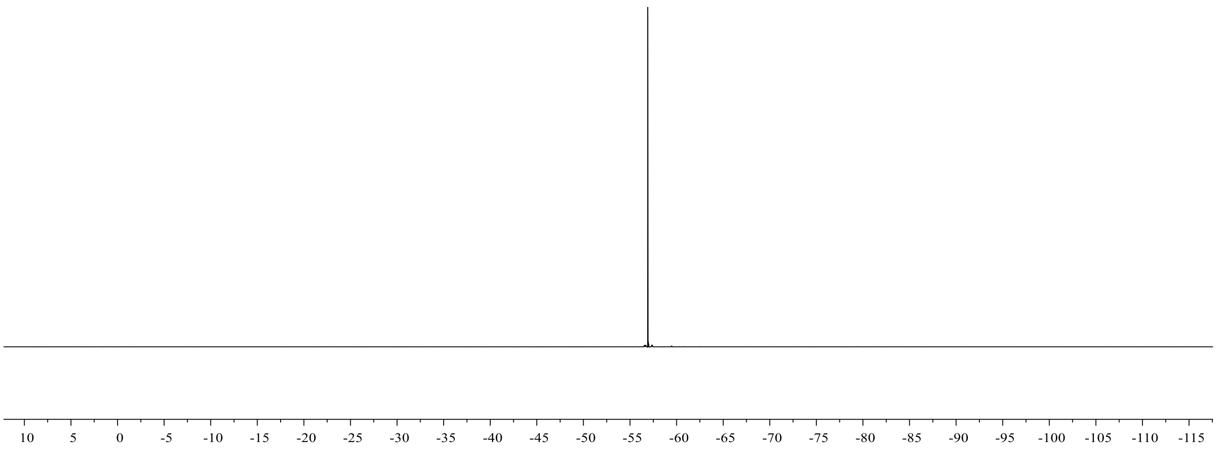

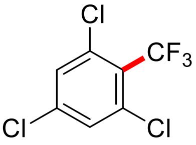

3h
(400 MHz, $CDCl_3$)

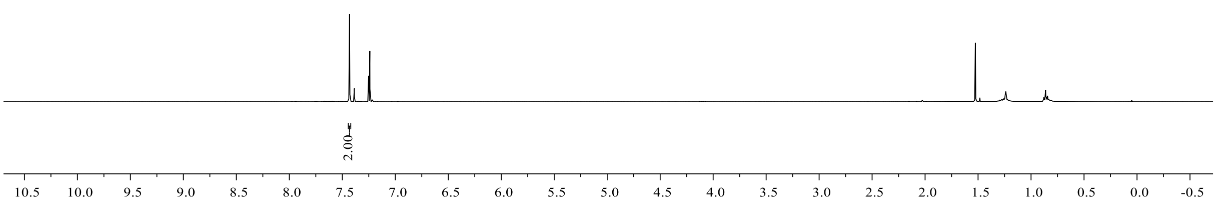

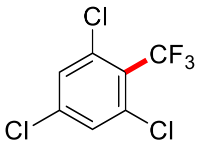

3h
(100 MHz, $CDCl_3$)

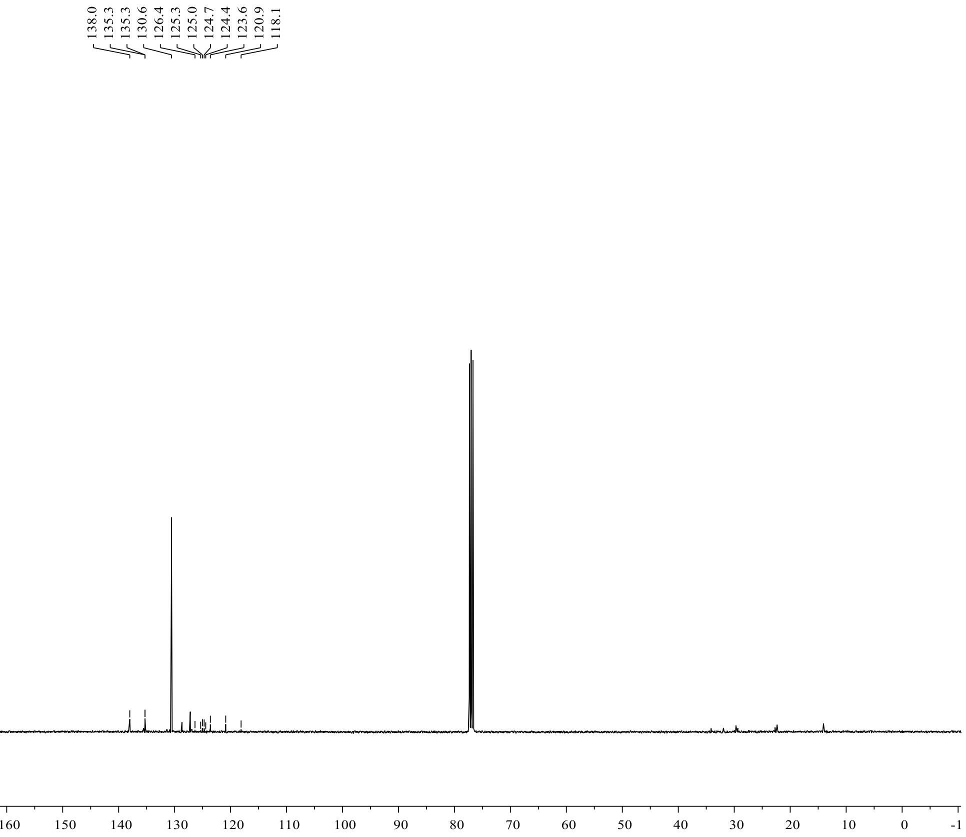

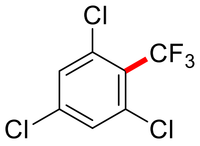

3h
(376 MHz, $CDCl_3$)

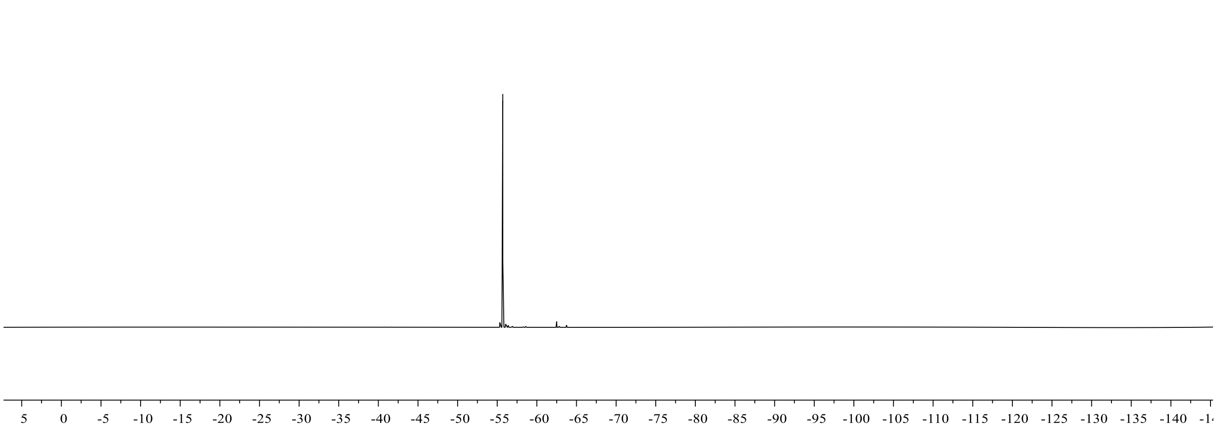

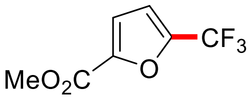

5a
(400 MHz, $CDCl_3$)

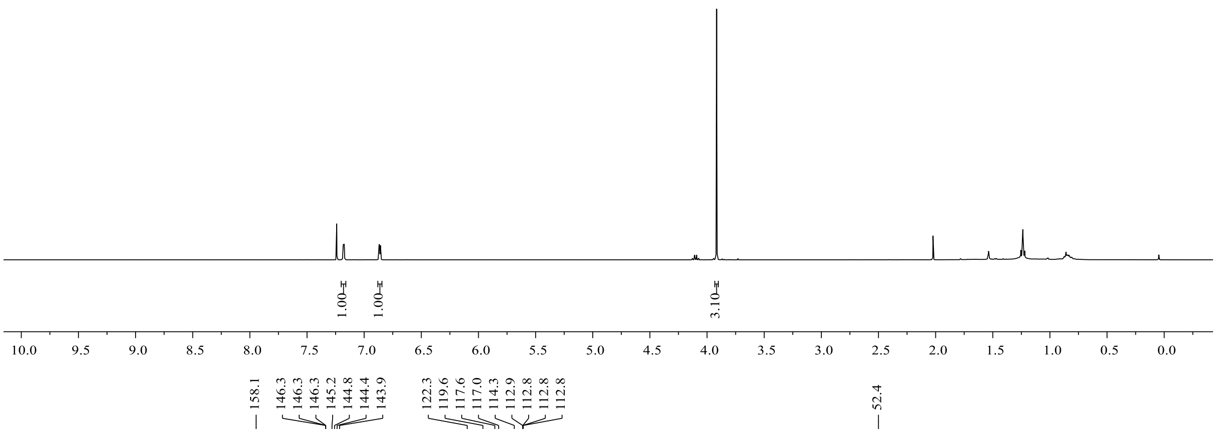

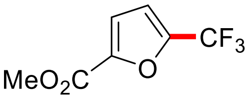

5a
(100 MHz, $CDCl_3$)

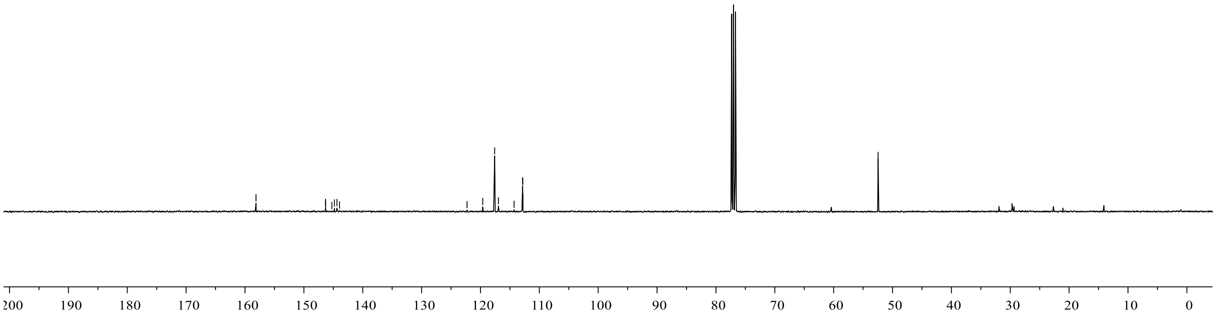

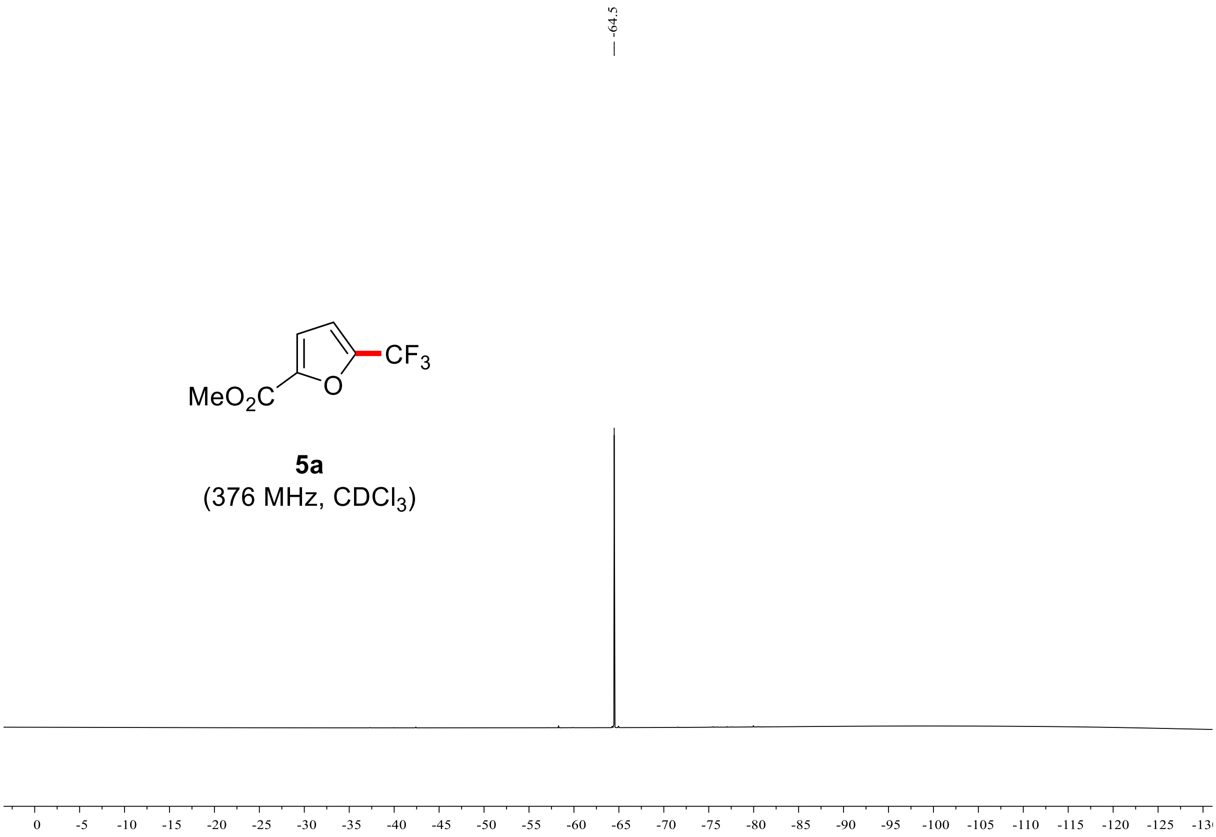

5a
(376 MHz, $CDCl_3$)

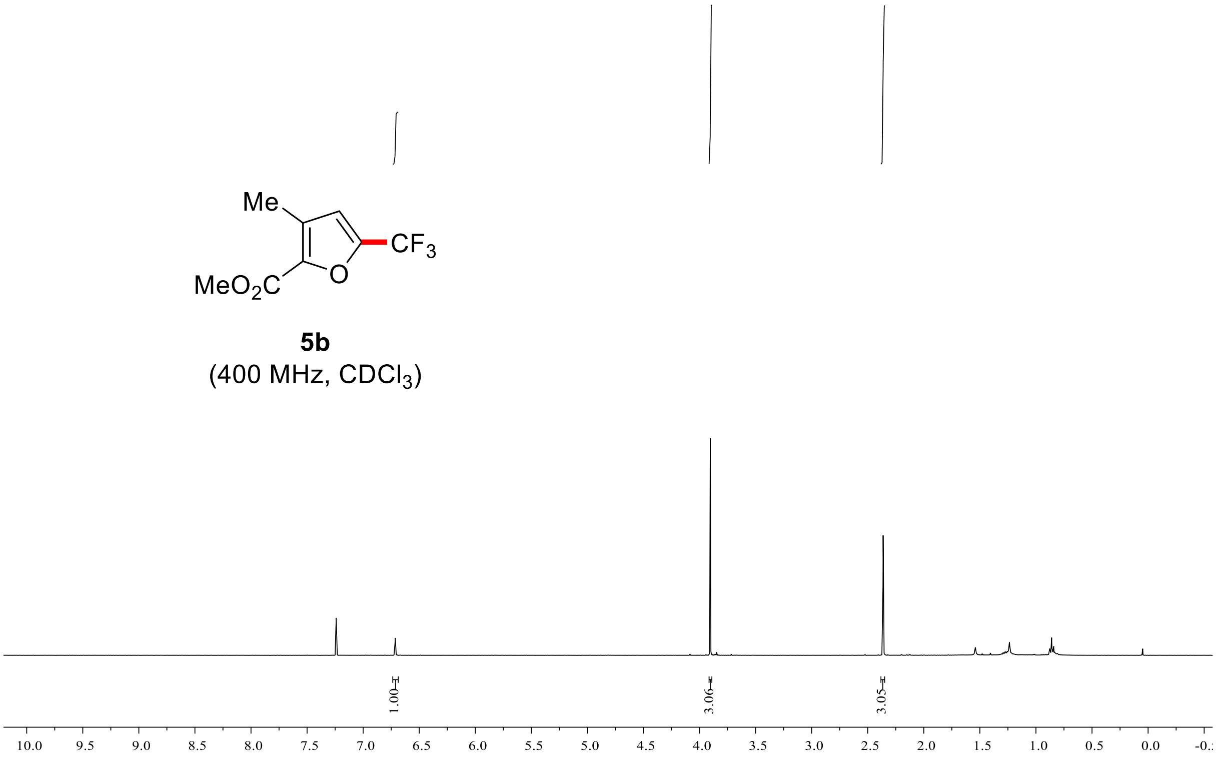

5b
(400 MHz, $CDCl_3$)

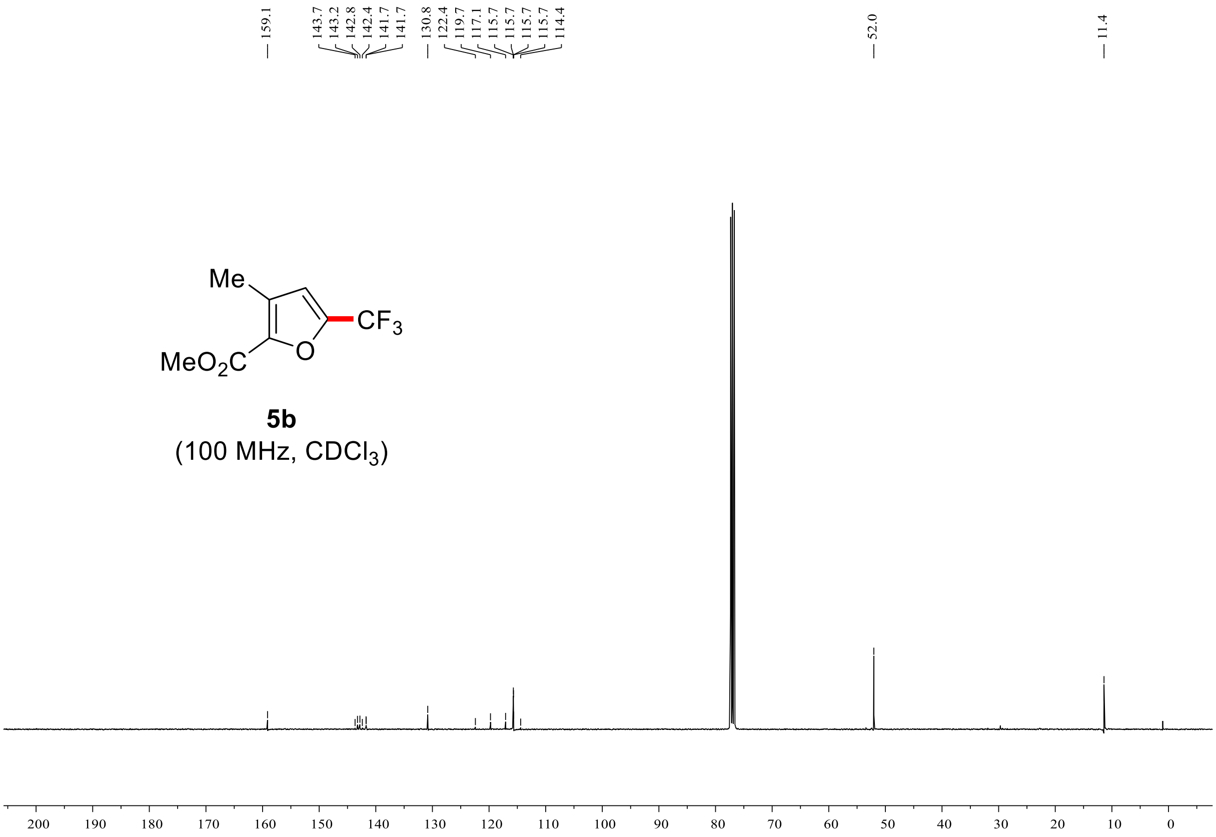

5b
(100 MHz, $CDCl_3$)

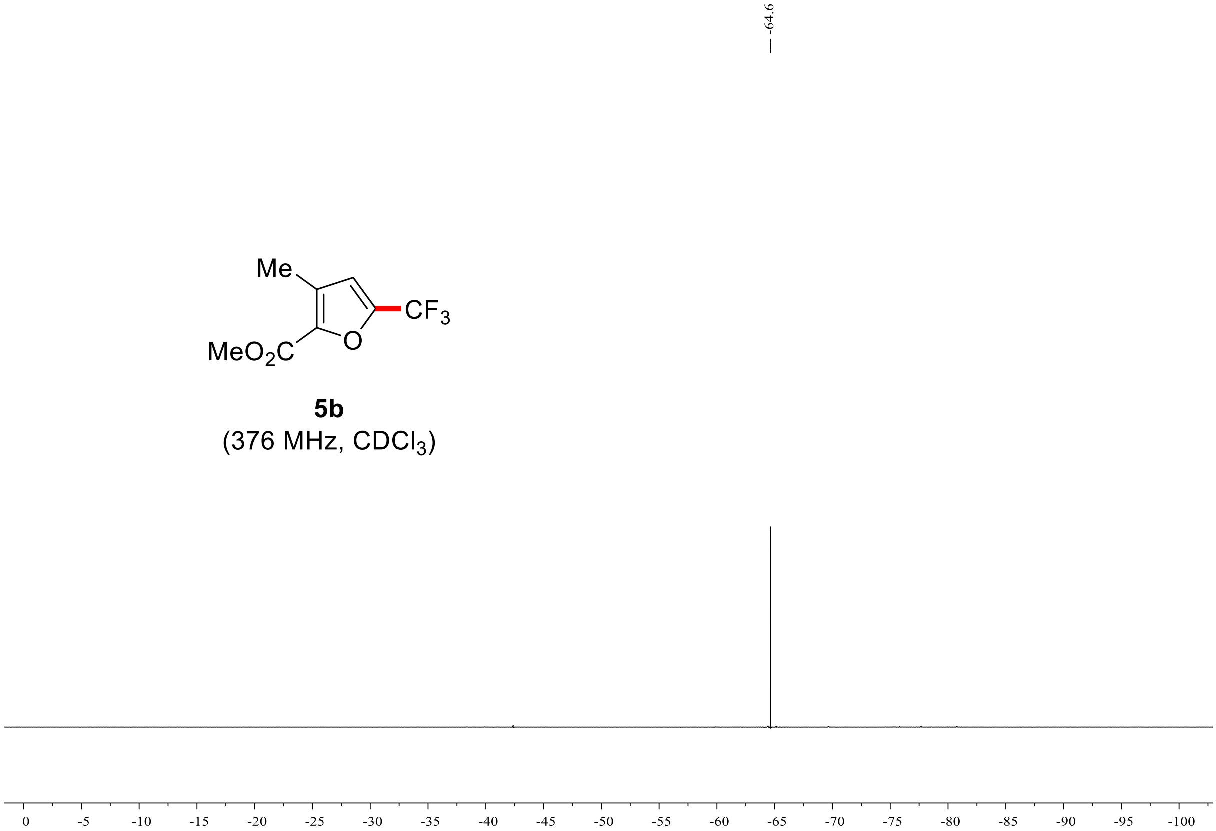

5b
(376 MHz, $CDCl_3$)

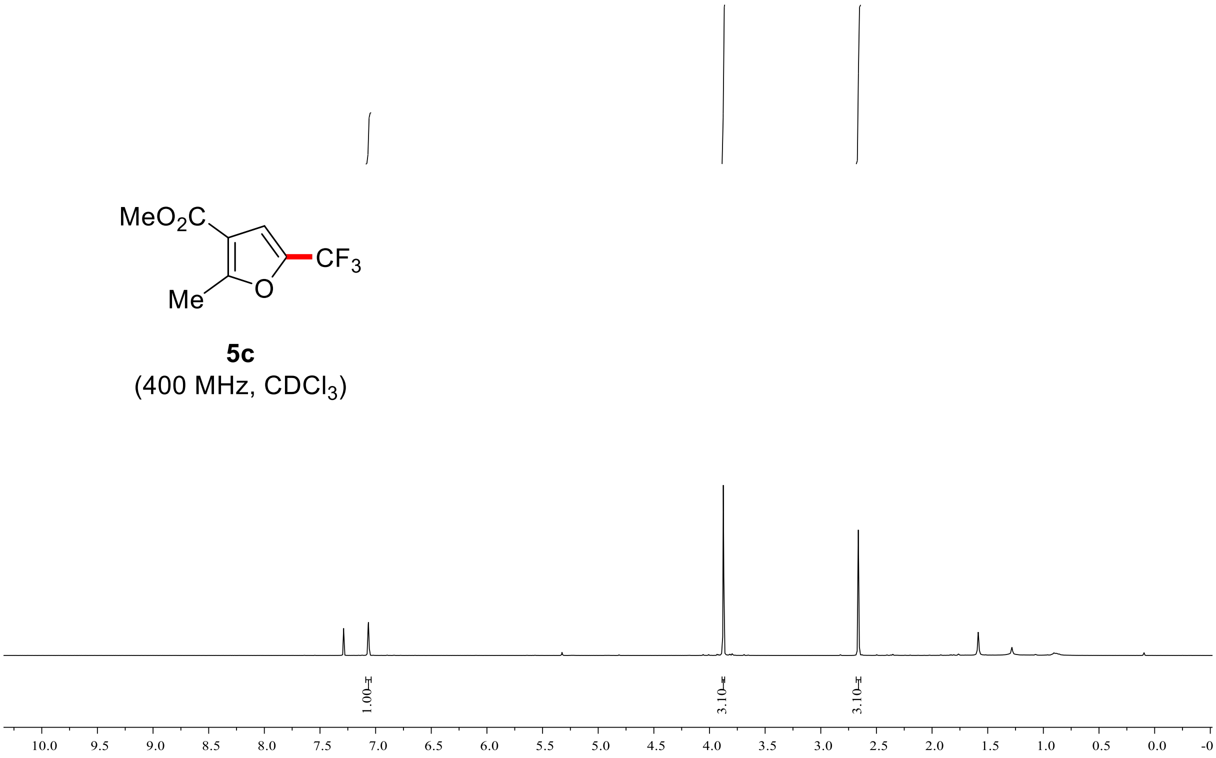
MeO2C
CF3
Me
O
5c
(400 MHz, CDCl3)
1.00
3.10
3.10
10.0 9.5 9.0 8.5 8.0 7.5 7.0 6.5 6.0 5.5 5.0 4.5 4.0 3.5 3.0 2.5 2.0 1.5 1.0 0.5 0.0 -0

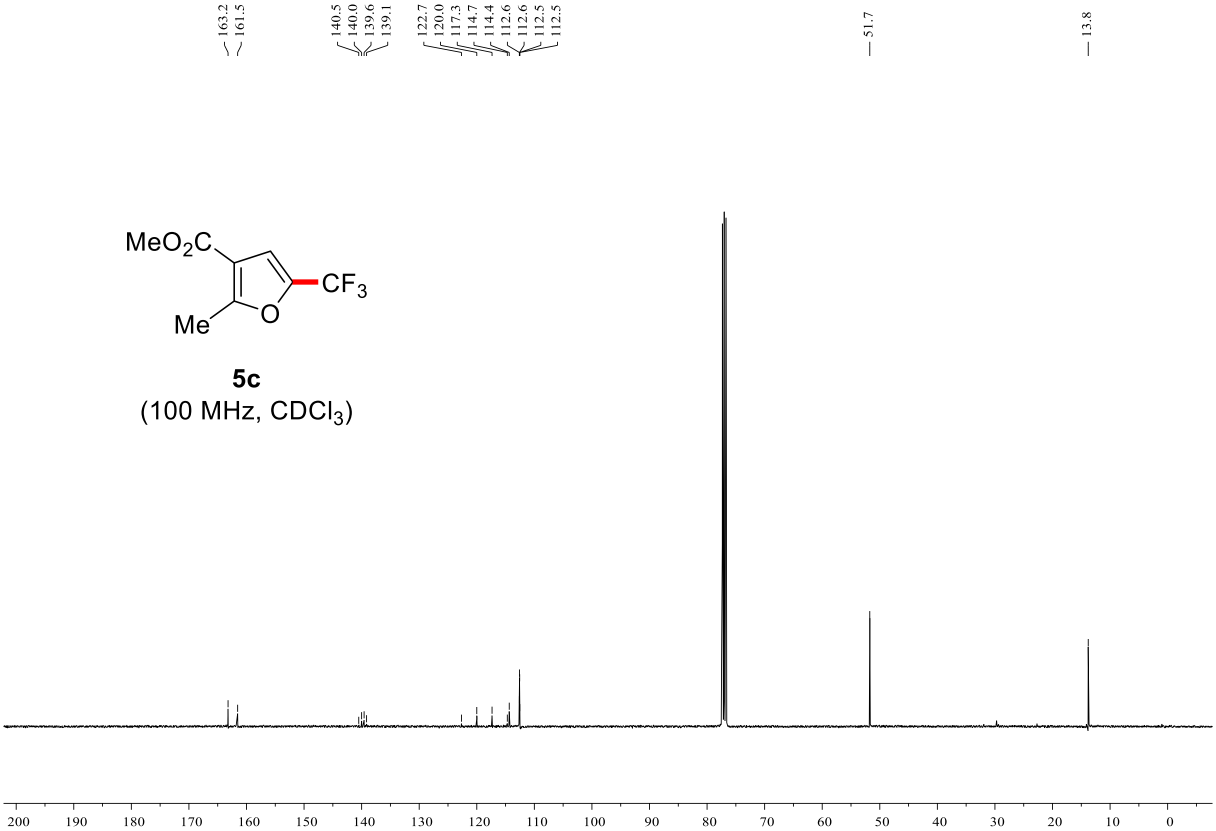
163.2
161.5
140.5
140.0
139.6
139.1
122.7
120.0
117.3
114.7
114.4
112.6
112.6
112.5
112.5
51.7
13.8
MeO2C
CF3
Me
O
5c
(100 MHz, CDCl3)
200 190 180 170 160 150 140 130 120 110 100 90 80 70 60 50 40 30 20 10 0

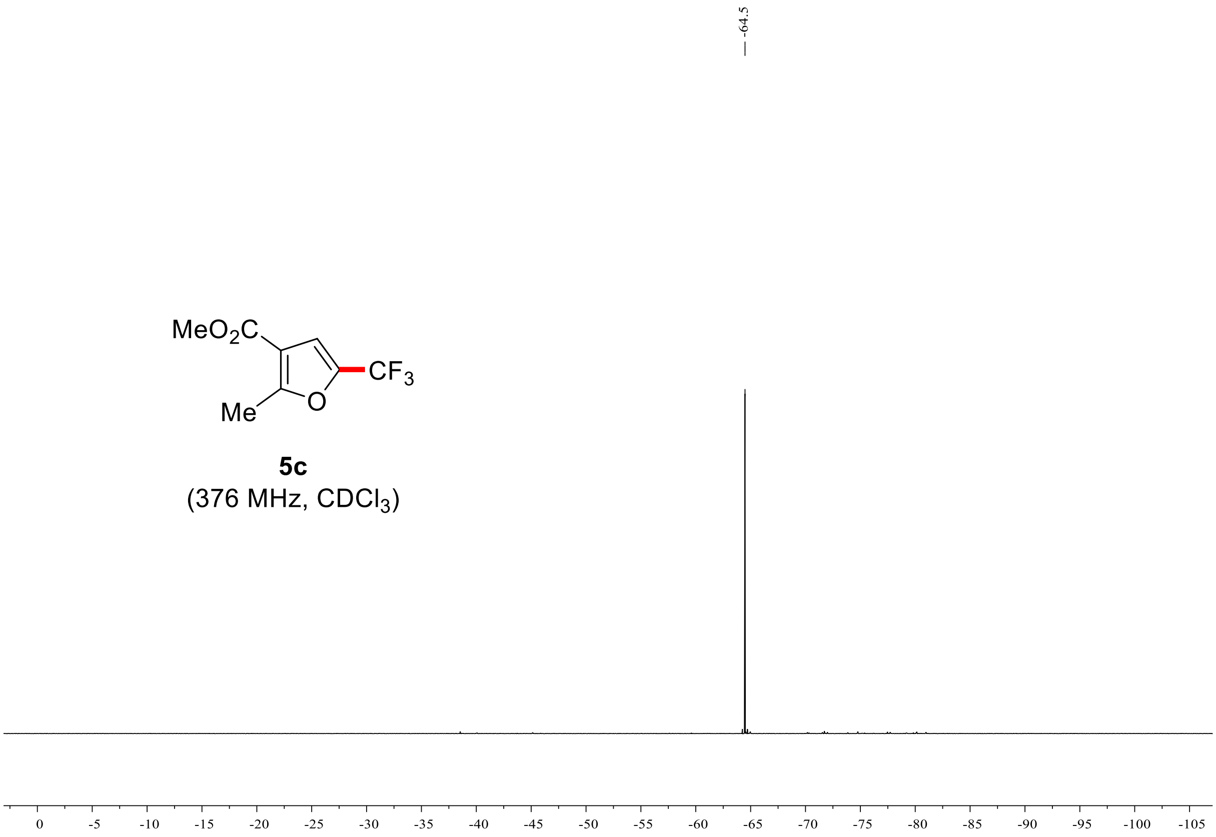

5c
(376 MHz, $CDCl_3$)

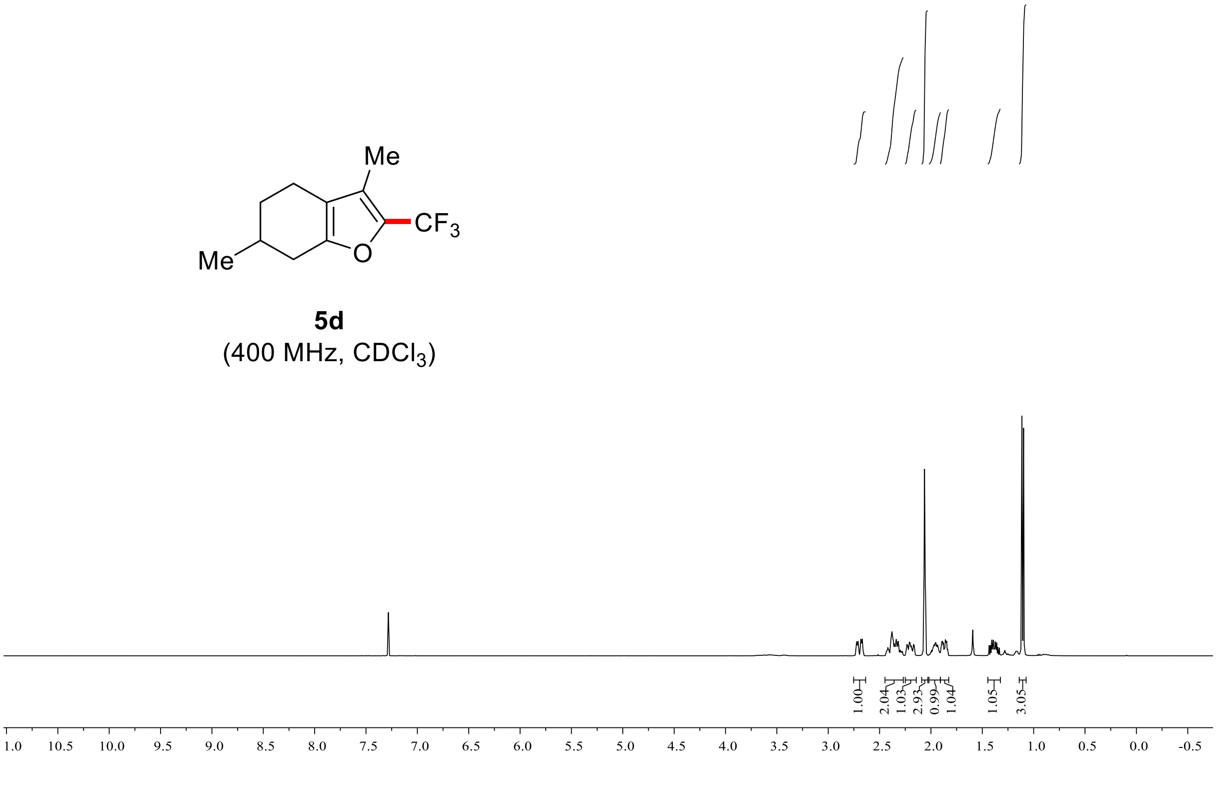

5d
(400 MHz, $CDCl_3$)

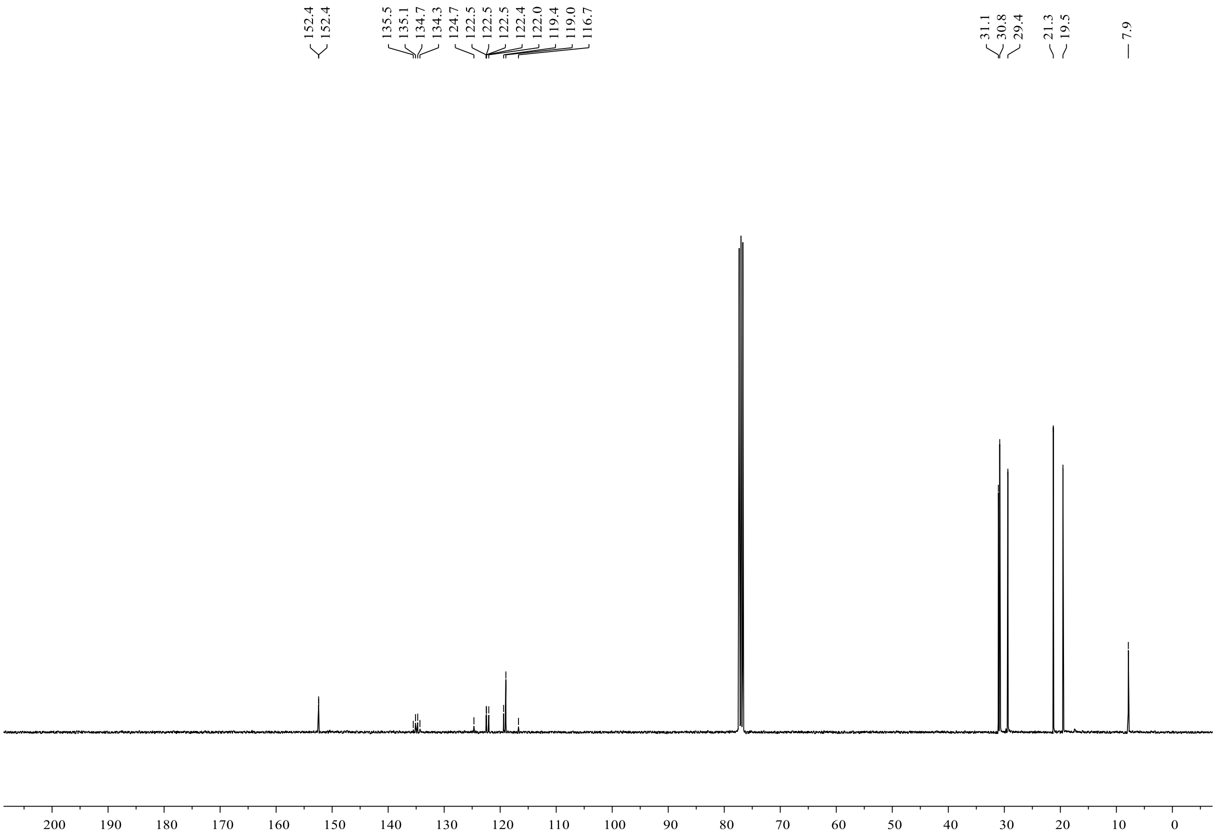

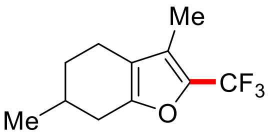

5d
(376 MHz, $CDCl_3$)

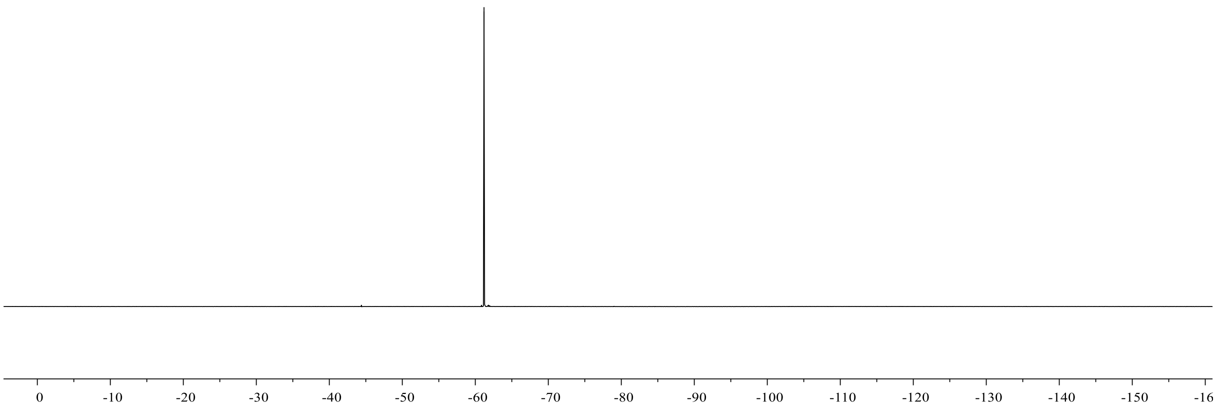

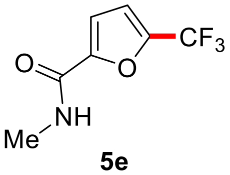

5e

(500 MHz, $CDCl_3$)

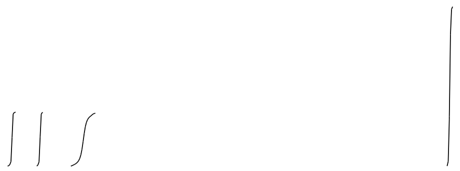

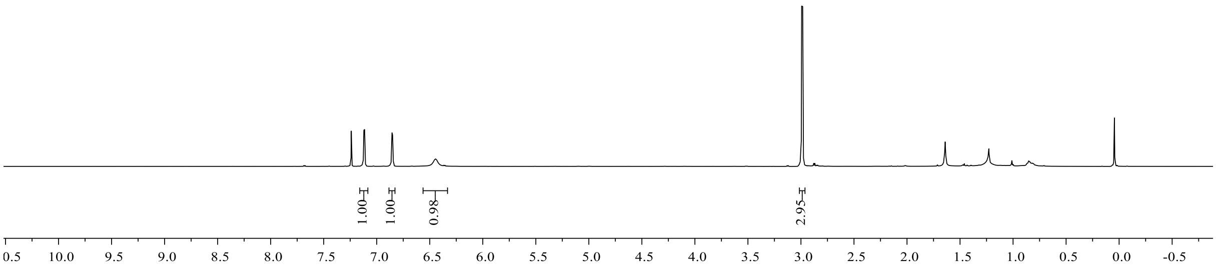

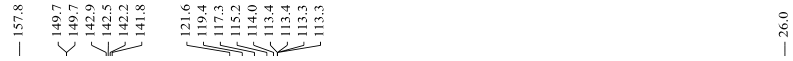

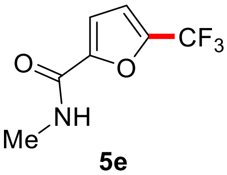

5e

(125 MHz, $CDCl_3$)

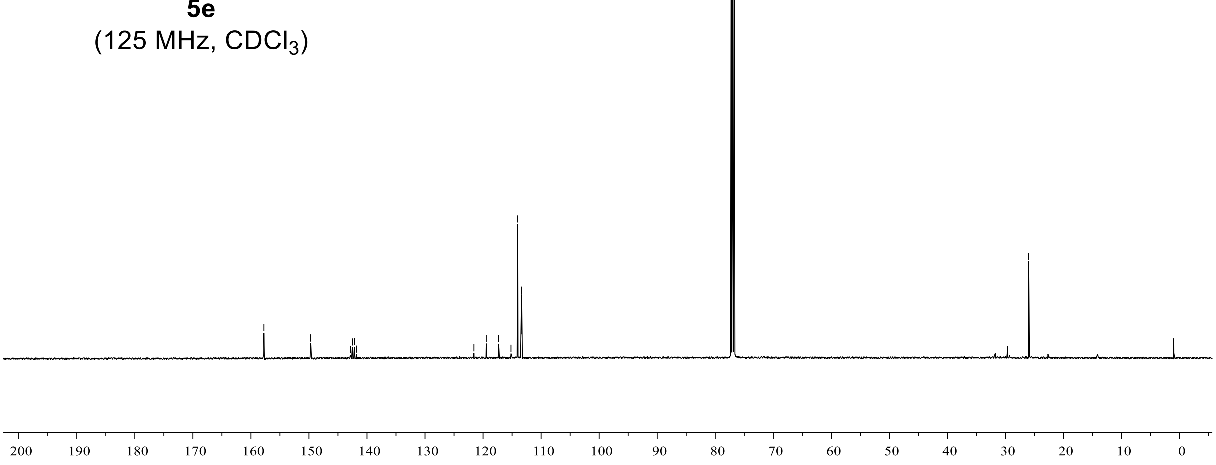

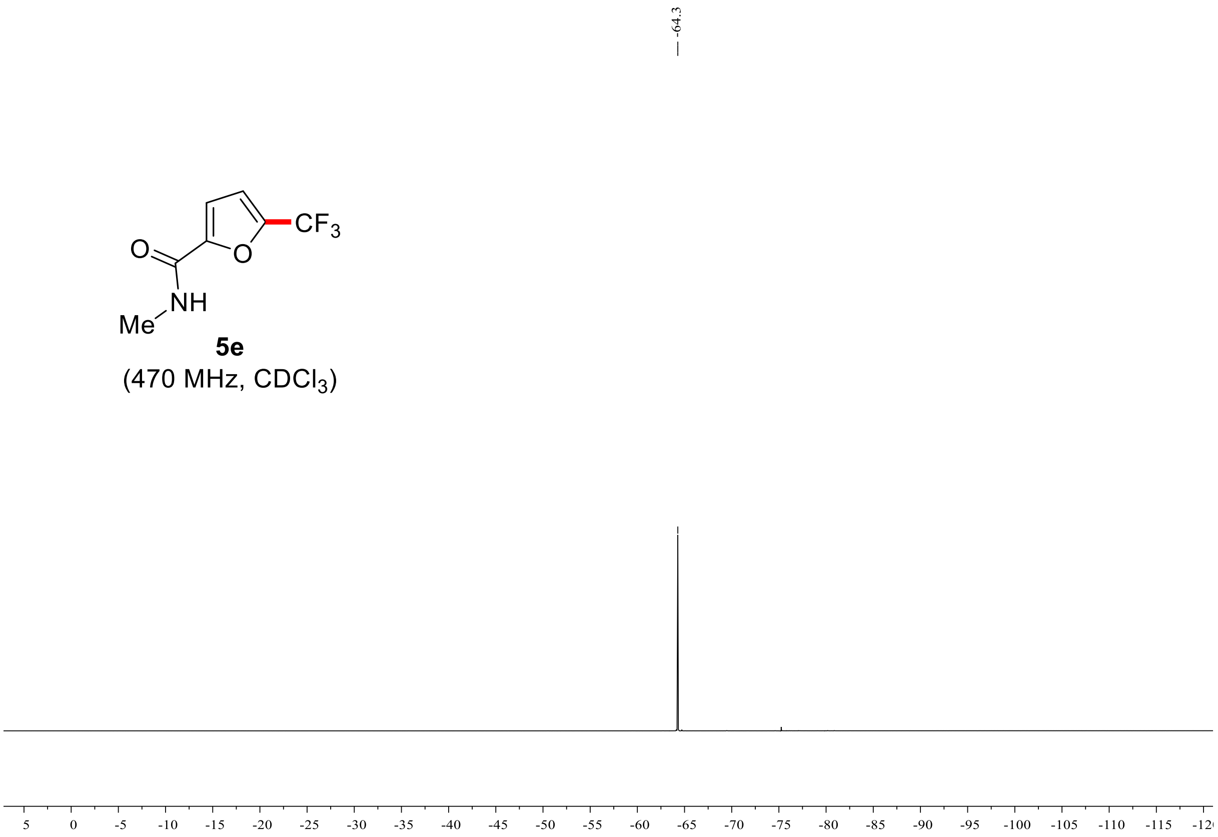
-64.3
CF3
O
NH
Me
5e
(470 MHz, CDCl3)
5 0 -5 -10 -15 -20 -25 -30 -35 -40 -45 -50 -55 -60 -65 -70 -75 -80 -85 -90 -95 -100 -105 -110 -115

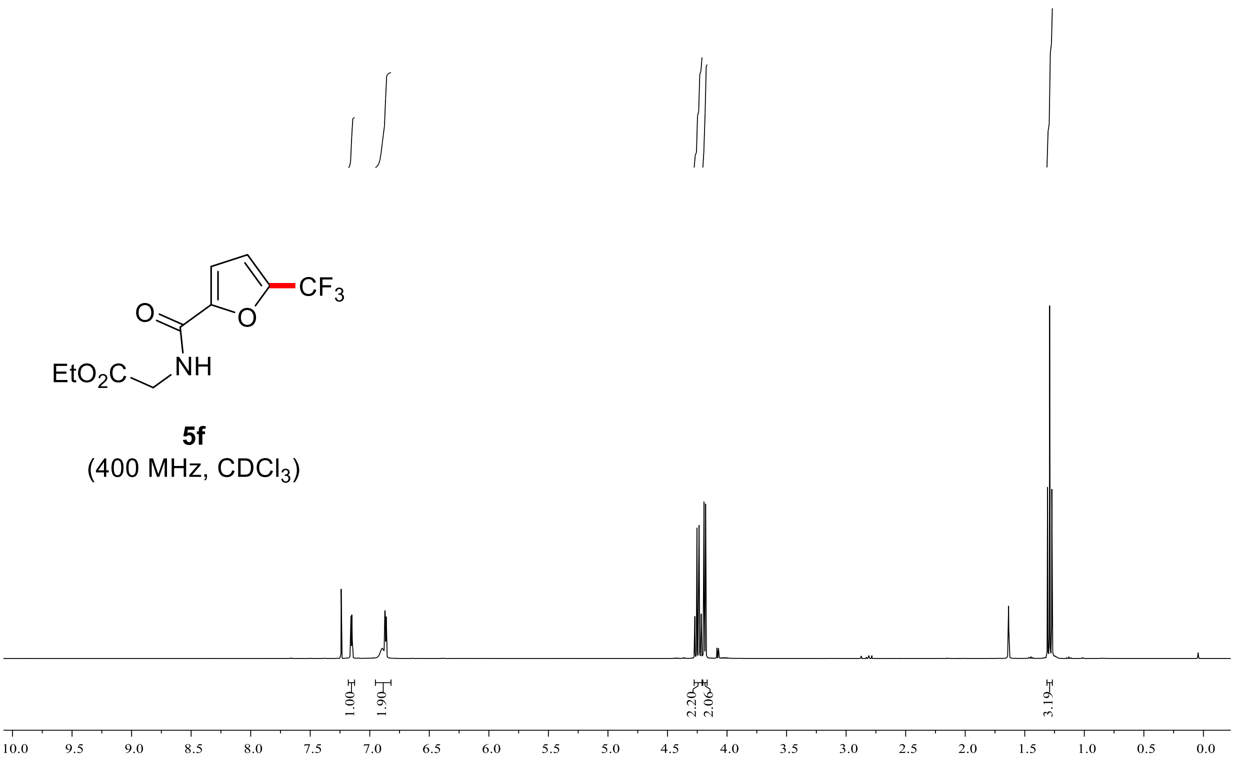
CF3
O
EtO2C
NH
5f
(400 MHz, CDCl3)
1.00
1.90
2.20
2.06
3.19
10.0 9.5 9.0 8.5 8.0 7.5 7.0 6.5 6.0 5.5 5.0 4.5 4.0 3.5 3.0 2.5 2.0 1.5 1.0 0.5 0.0

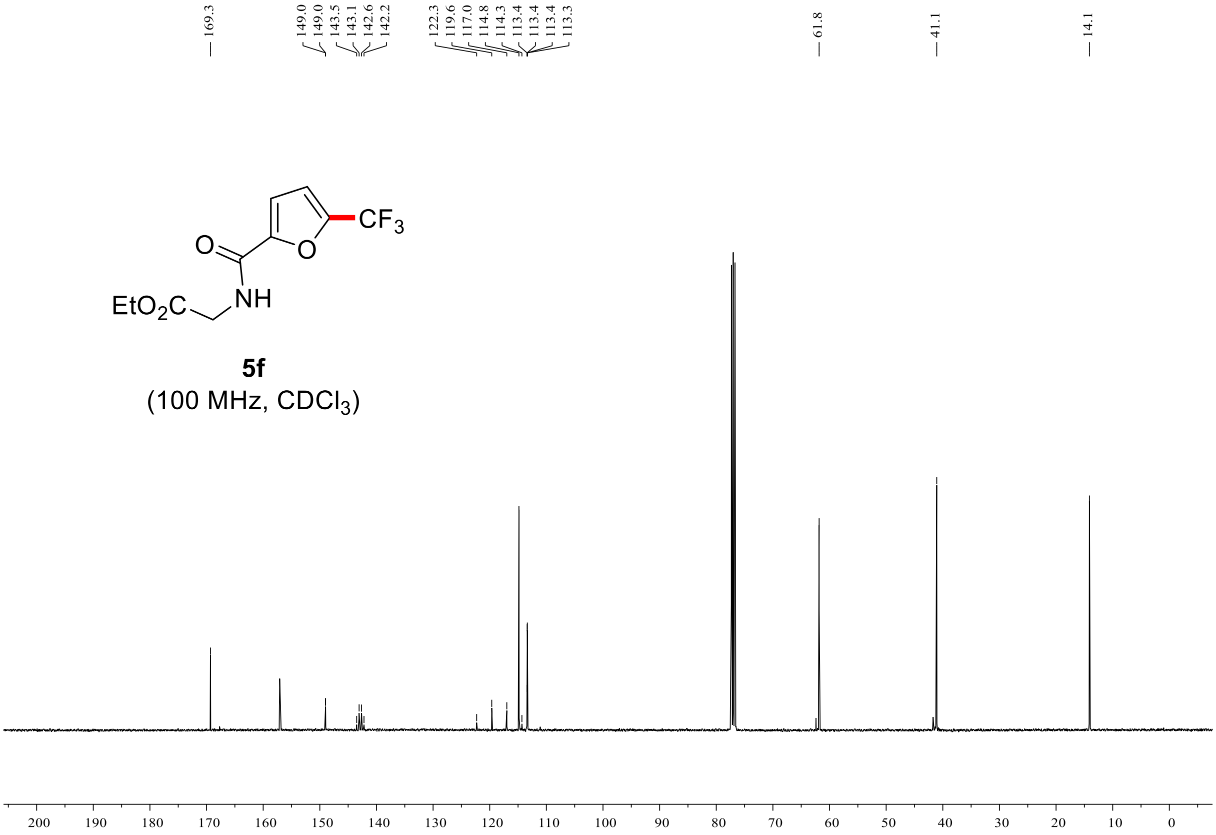
169.3
149.0
149.0
143.5
143.1
142.6
142.2
122.3
119.6
117.0
114.8
114.3
113.4
113.4
113.4
113.3
61.8
41.1
14.1
CF3
O
O
NH
EtO2C
5f
(100 MHz, CDCl3)
200 190 180 170 160 150 140 130 120 110 100 90 80 70 60 50 40 30 20 10 0

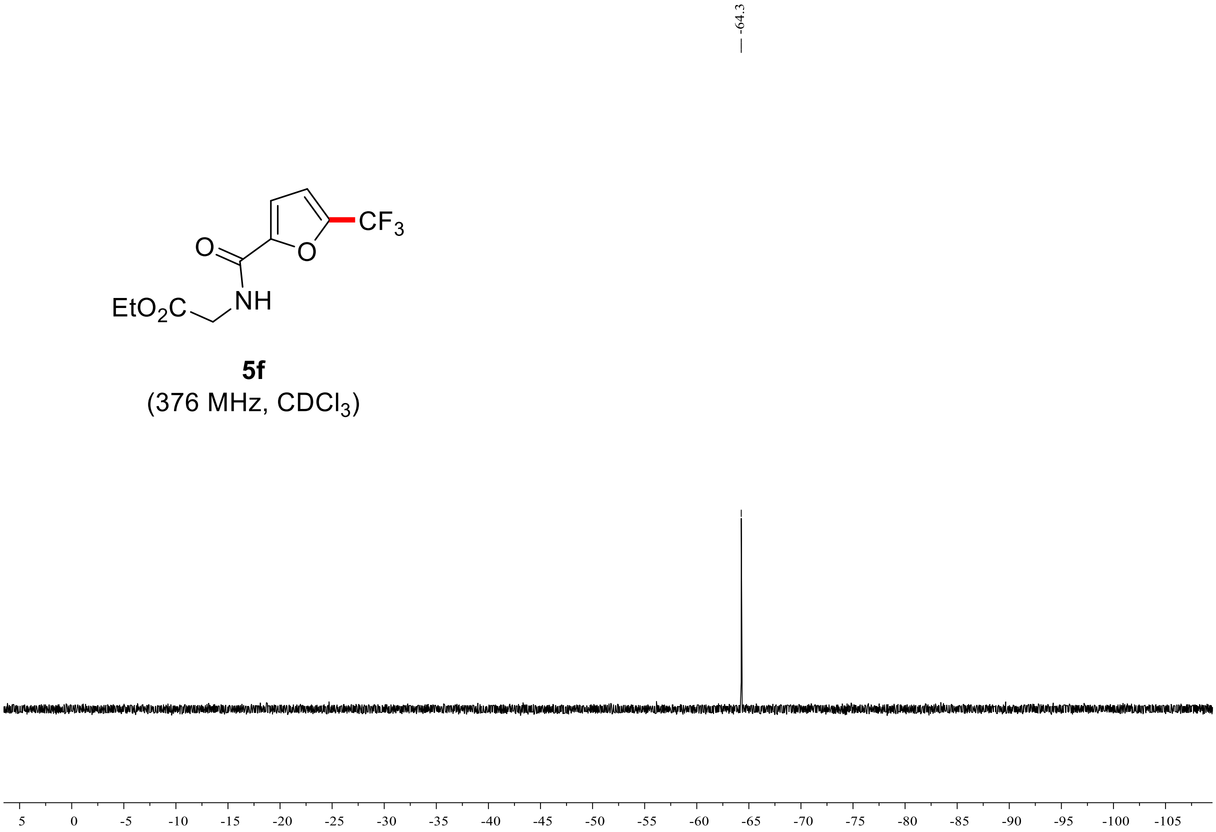
-64.3
CF3
O
O
NH
EtO2C
5f
(376 MHz, CDCl3)
5 0 -5 -10 -15 -20 -25 -30 -35 -40 -45 -50 -55 -60 -65 -70 -75 -80 -85 -90 -95 -100 -105

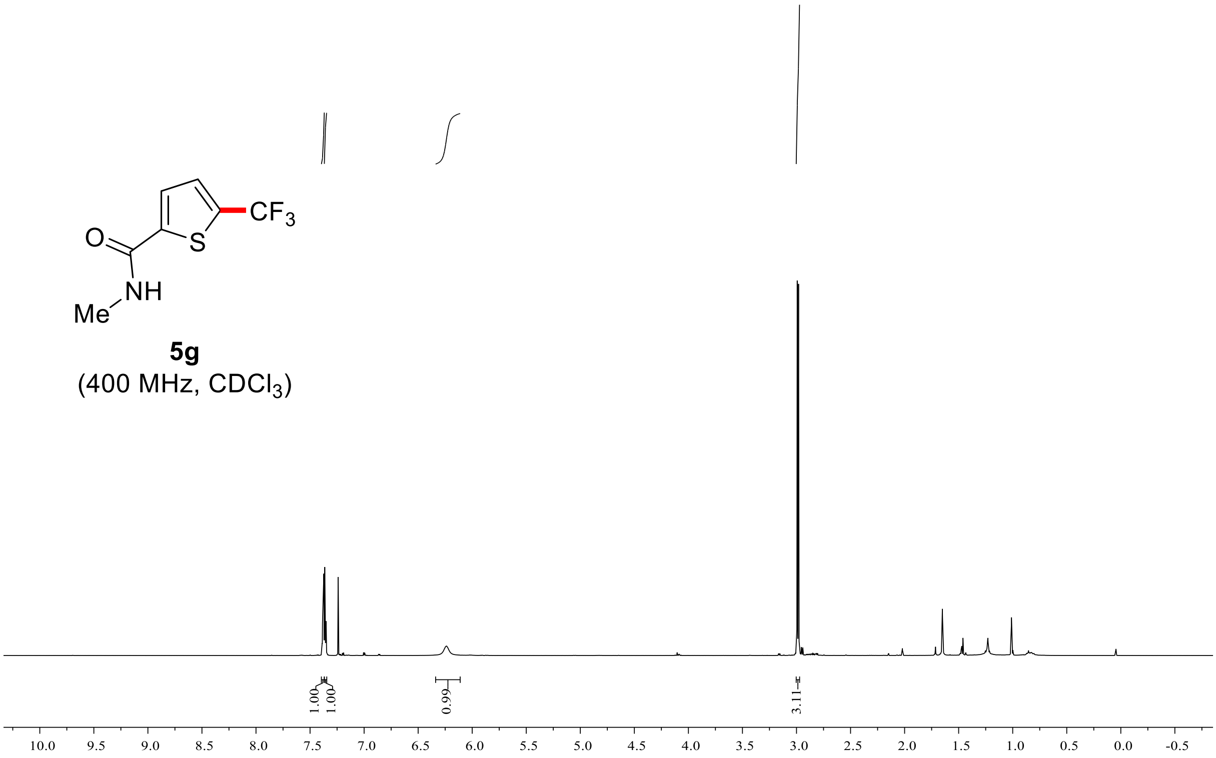
CF3
O
S
NH
Me
5g
(400 MHz, CDCl3)
1.00
1.00
0.99
3.11
10.0 9.5 9.0 8.5 8.0 7.5 7.0 6.5 6.0 5.5 5.0 4.5 4.0 3.5 3.0 2.5 2.0 1.5 1.0 0.5 0.0 -0.5

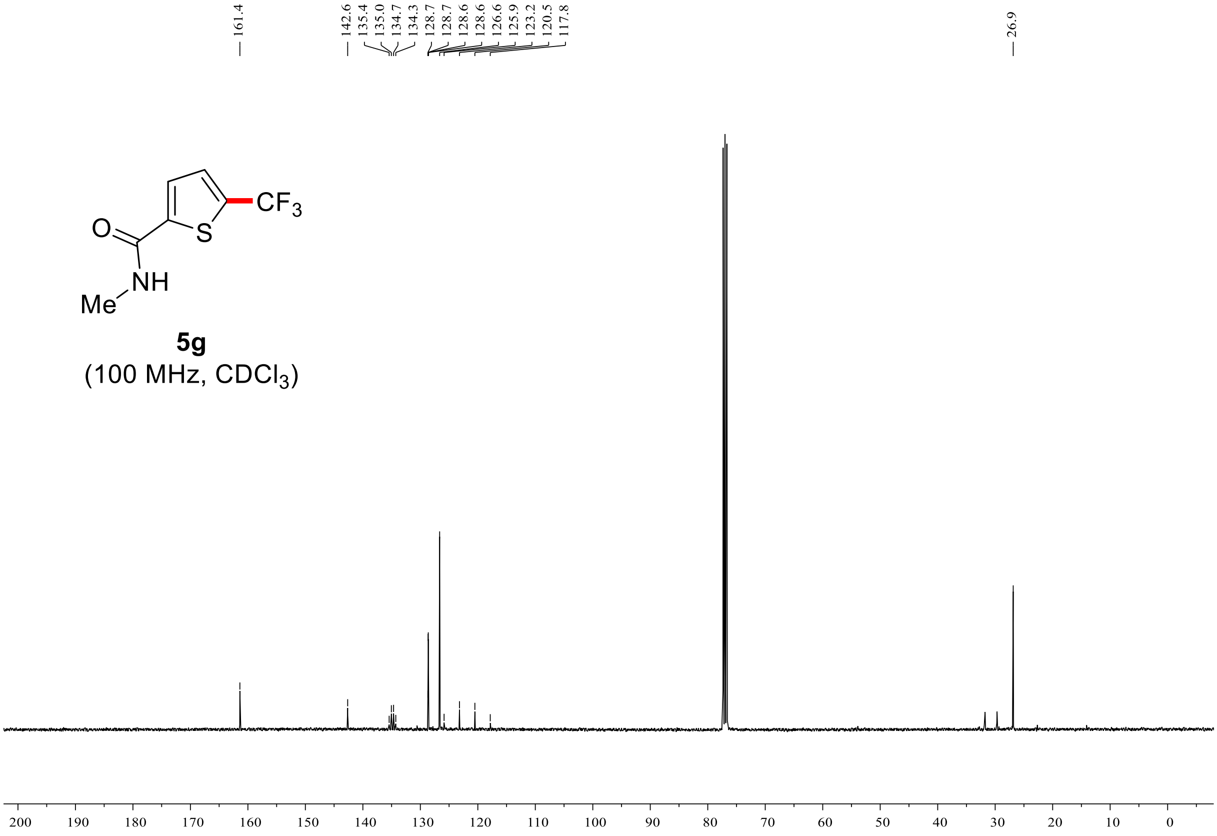
161.4
142.6
135.4
135.0
134.7
134.3
128.7
128.7
128.6
128.6
126.6
125.9
123.2
120.5
117.8
26.9
CF3
O
S
NH
Me
5g
(100 MHz, CDCl3)
200 190 180 170 160 150 140 130 120 110 100 90 80 70 60 50 40 30 20 10 0

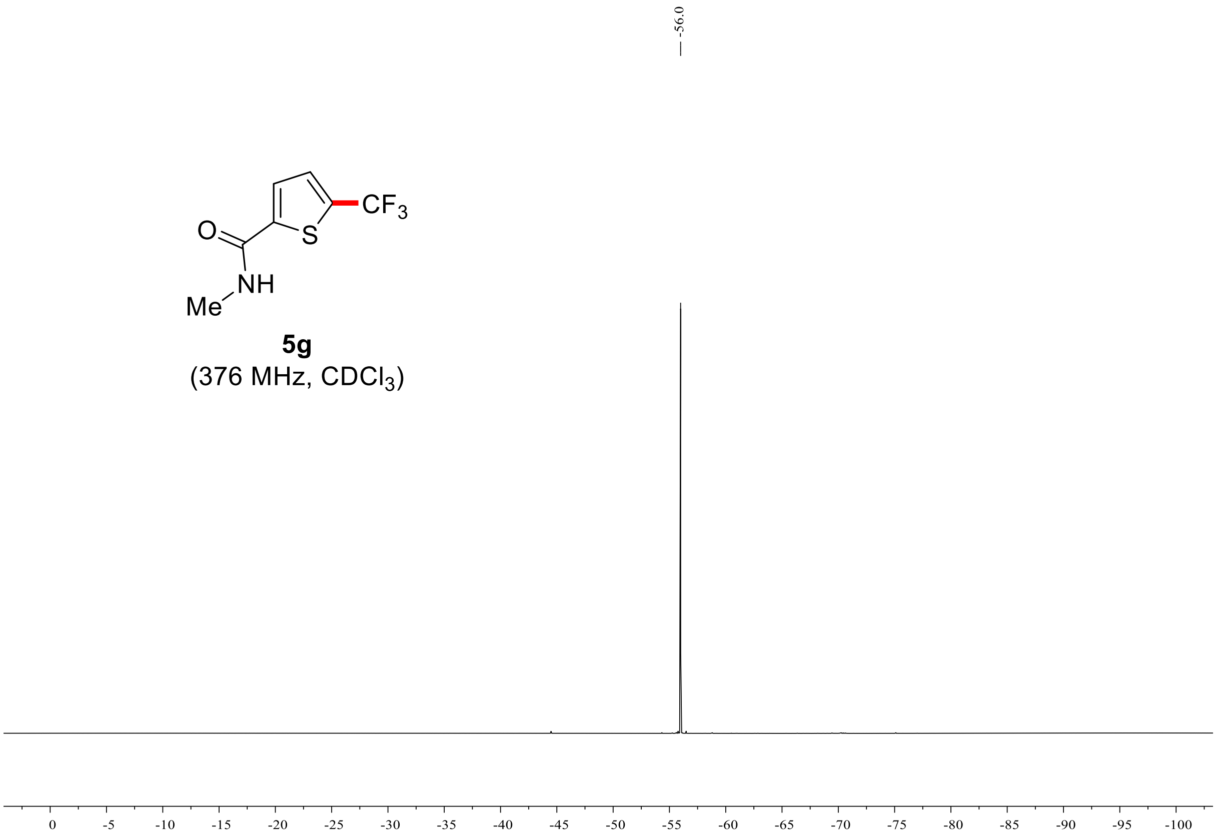

5g
(376 MHz, $CDCl_3$)

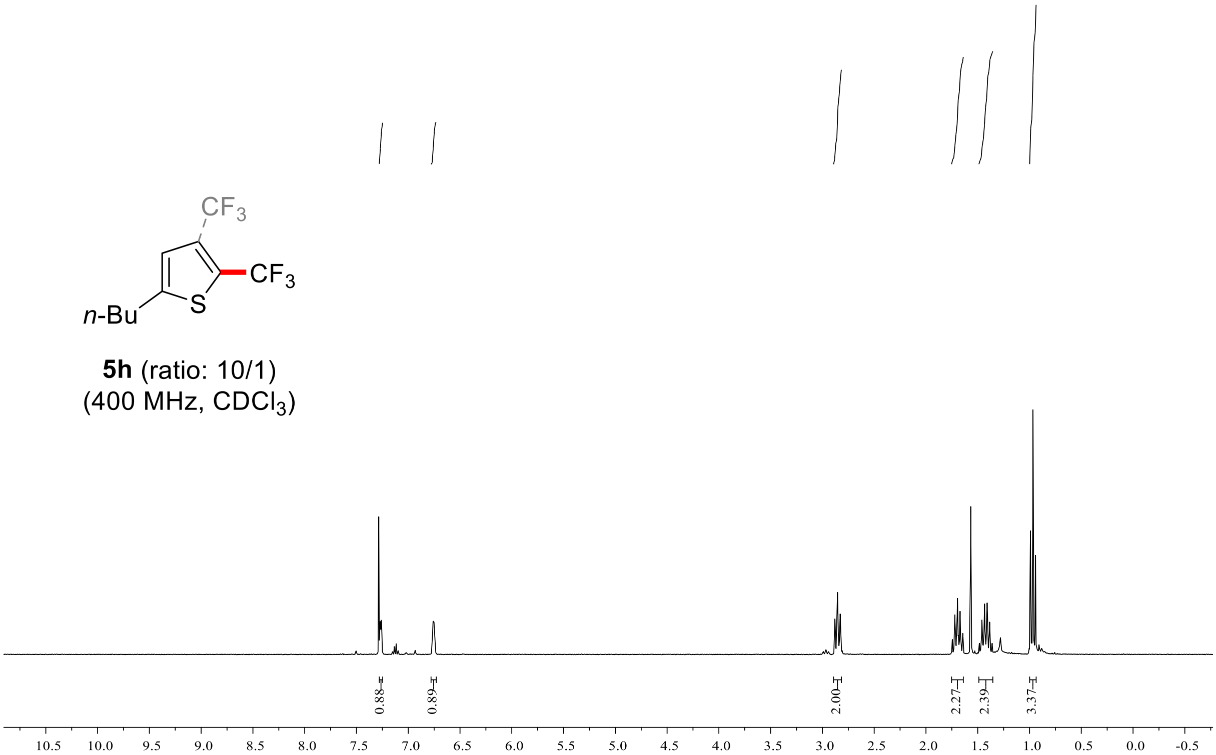

5h (ratio: 10/1)
(400 MHz, $CDCl_3$)

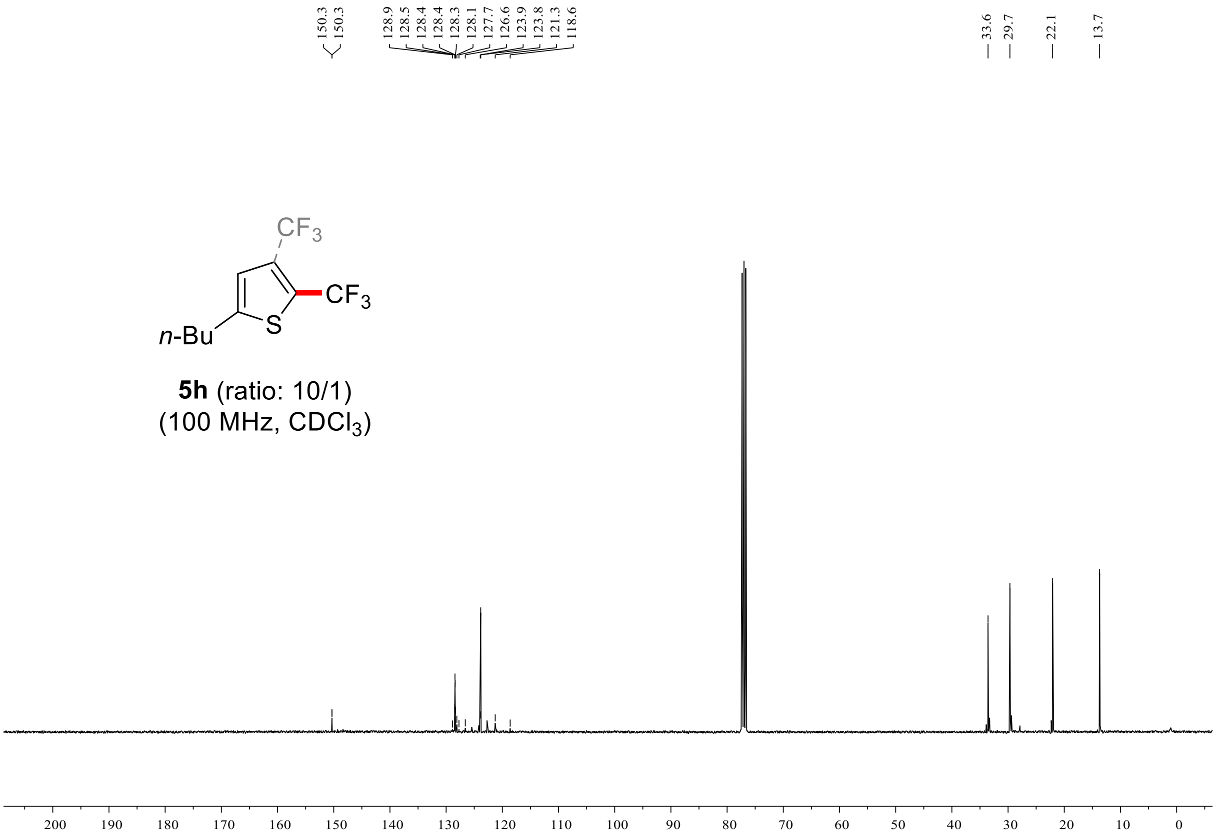
150.3
150.3
128.9
128.5
128.4
128.4
128.3
128.1
127.7
126.6
123.9
123.8
121.3
118.6
33.6
29.7
22.1
13.7
CF3
CF3
n-Bu
S
5h (ratio: 10/1)
(100 MHz, CDCl3)
200 190 180 170 160 150 140 130 120 110 100 90 80 70 60 50 40 30 20 10 0

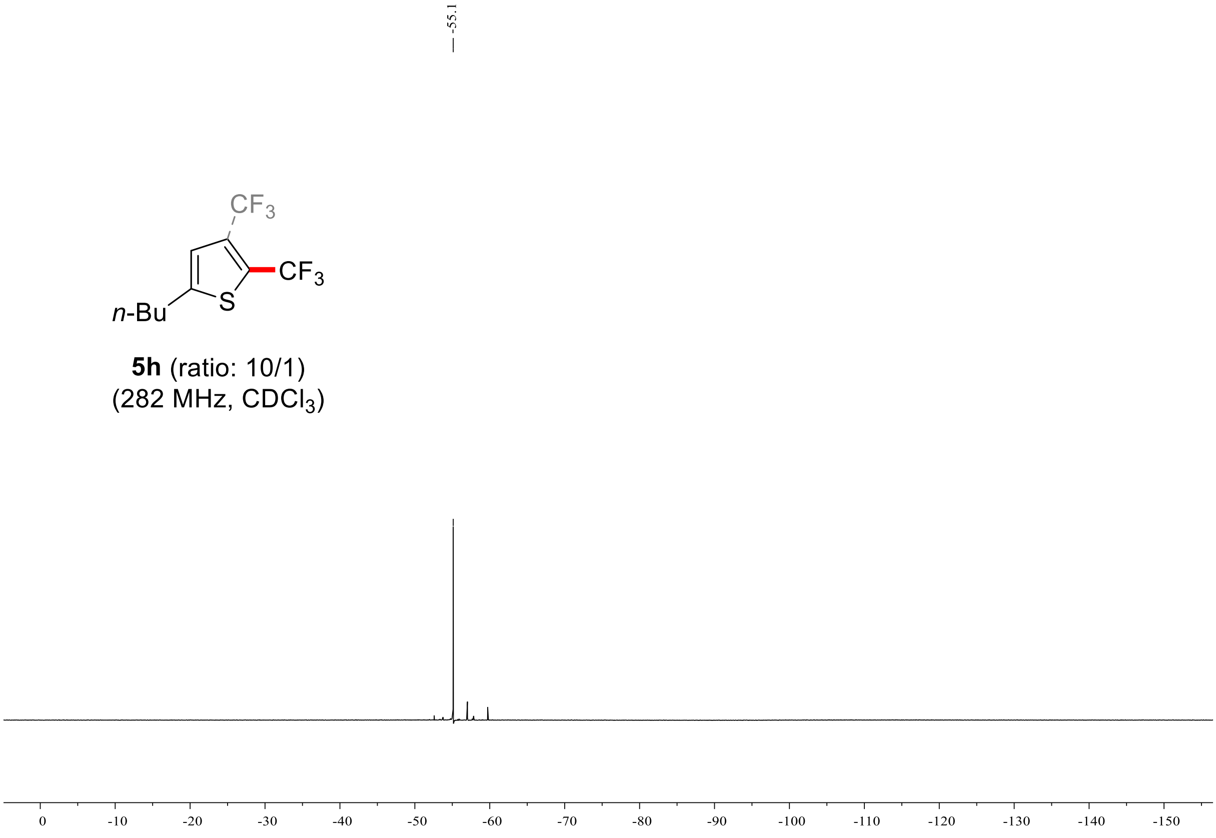
-55.1
CF3
CF3
n-Bu
S
5h (ratio: 10/1)
(282 MHz, CDCl3)
0 -10 -20 -30 -40 -50 -60 -70 -80 -90 -100 -110 -120 -130 -140 -150

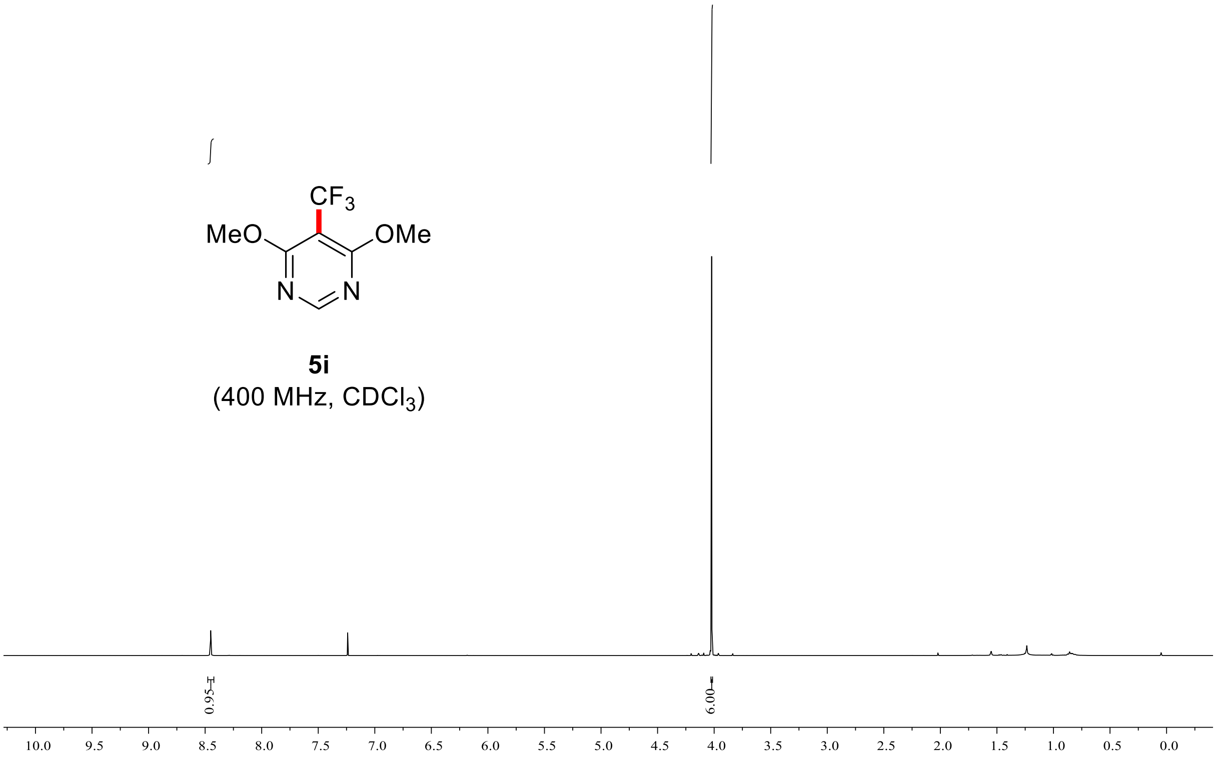
CF3
MeO
OMe
N
N
5i
(400 MHz, CDCl3)
0.95
6.00
10.0 9.5 9.0 8.5 8.0 7.5 7.0 6.5 6.0 5.5 5.0 4.5 4.0 3.5 3.0 2.5 2.0 1.5 1.0 0.5 0.0

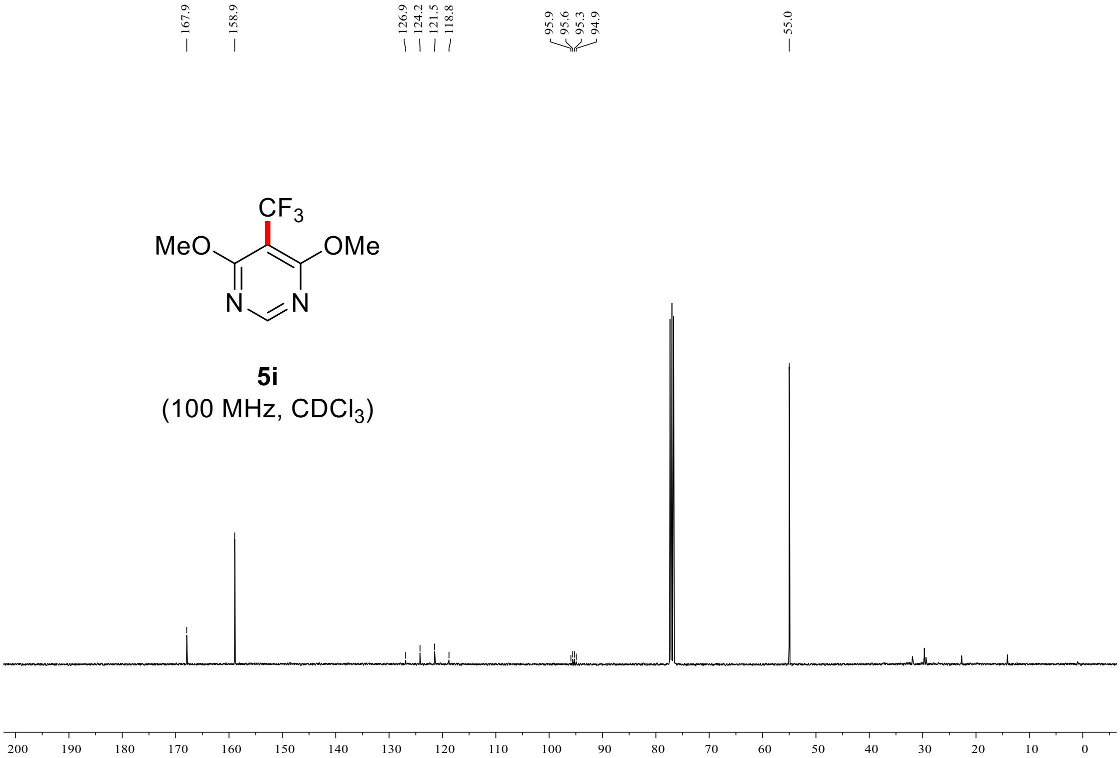
167.9
158.9
126.9
124.2
121.5
118.8
95.9
95.6
95.3
94.9
55.0
CF3
MeO
OMe
N
N
5i
(100 MHz, CDCl3)
200 190 180 170 160 150 140 130 120 110 100 90 80 70 60 50 40 30 20 10 0

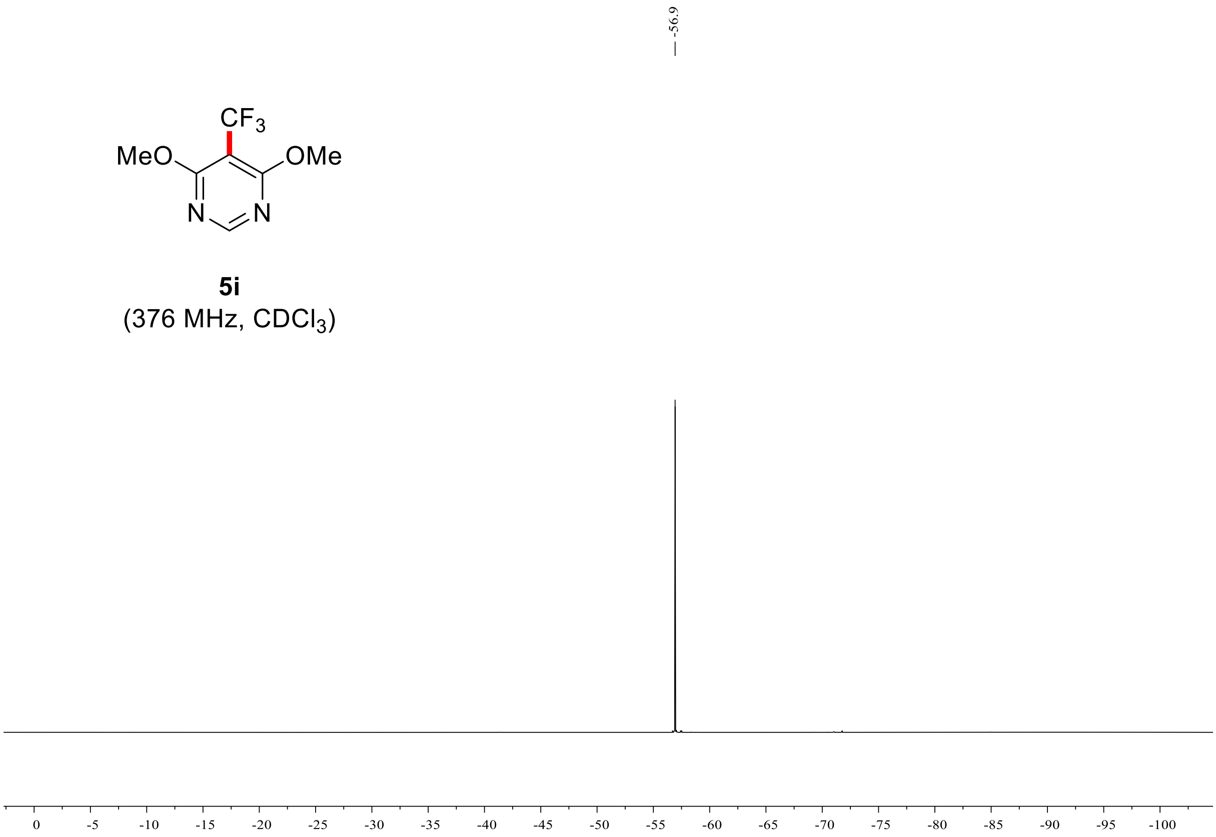
-56.9
CF3
MeO
OMe
N
N
5i
(376 MHz, CDCl3)
0 -5 -10 -15 -20 -25 -30 -35 -40 -45 -50 -55 -60 -65 -70 -75 -80 -85 -90 -95 -100

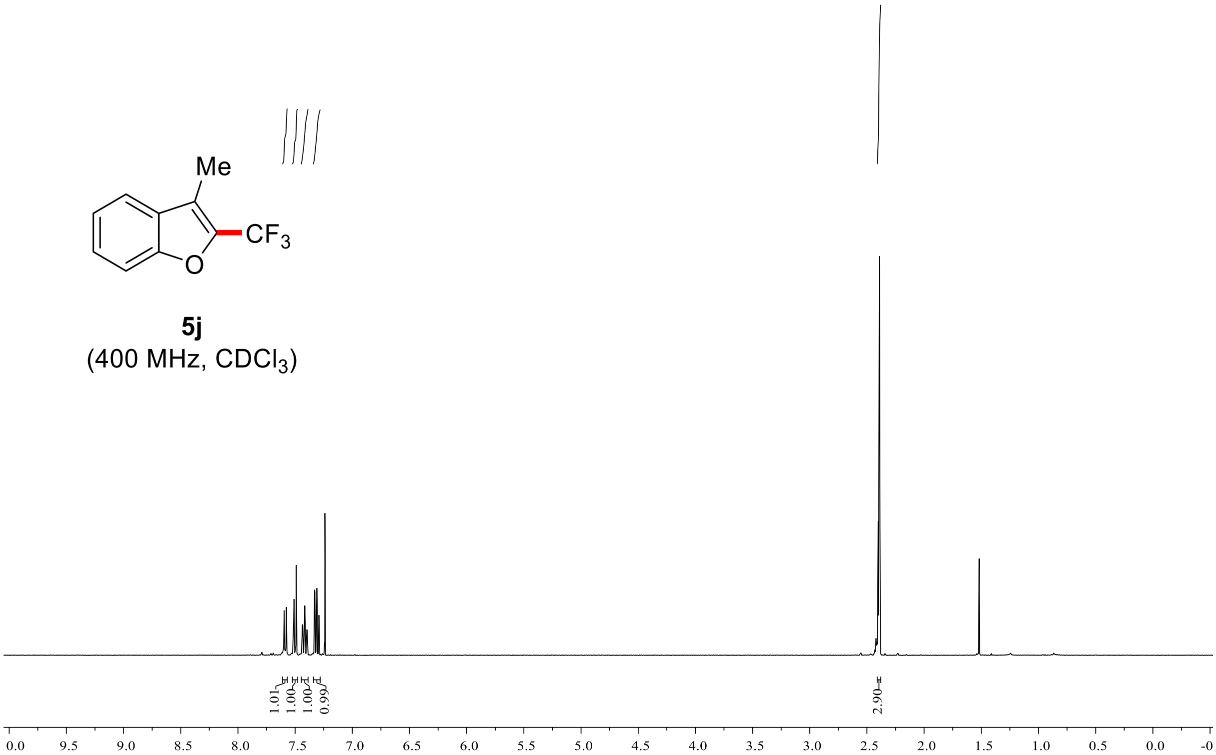
Me
CF3
O
5j
(400 MHz, CDCl3)
1.01
1.00
1.00
0.99
2.90
0.0 9.5 9.0 8.5 8.0 7.5 7.0 6.5 6.0 5.5 5.0 4.5 4.0 3.5 3.0 2.5 2.0 1.5 1.0 0.5 0.0 -0

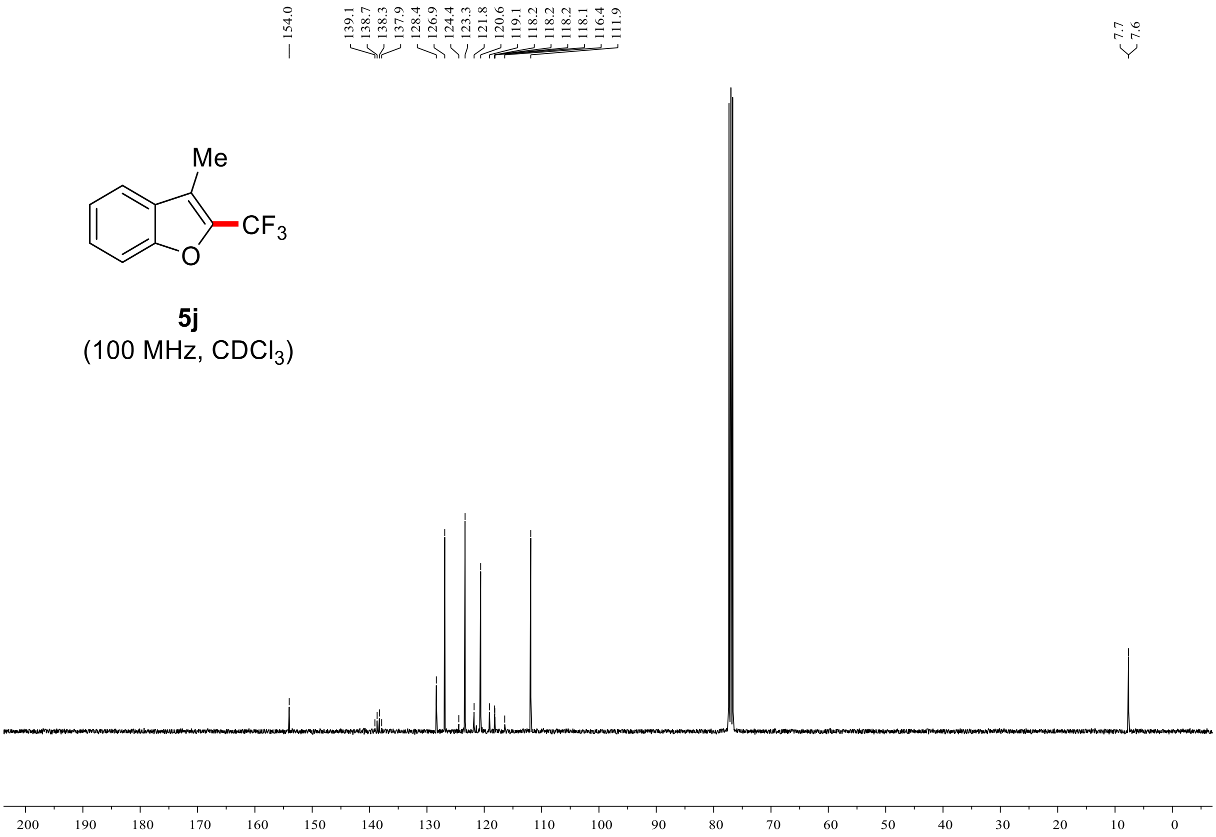
154.0
139.1
138.7
138.3
137.9
128.4
126.9
124.4
123.3
121.8
120.6
119.1
118.2
118.2
118.2
118.1
116.4
111.9
7.7
7.6
Me
CF3
O
5j
(100 MHz, CDCl3)
200 190 180 170 160 150 140 130 120 110 100 90 80 70 60 50 40 30 20 10 0

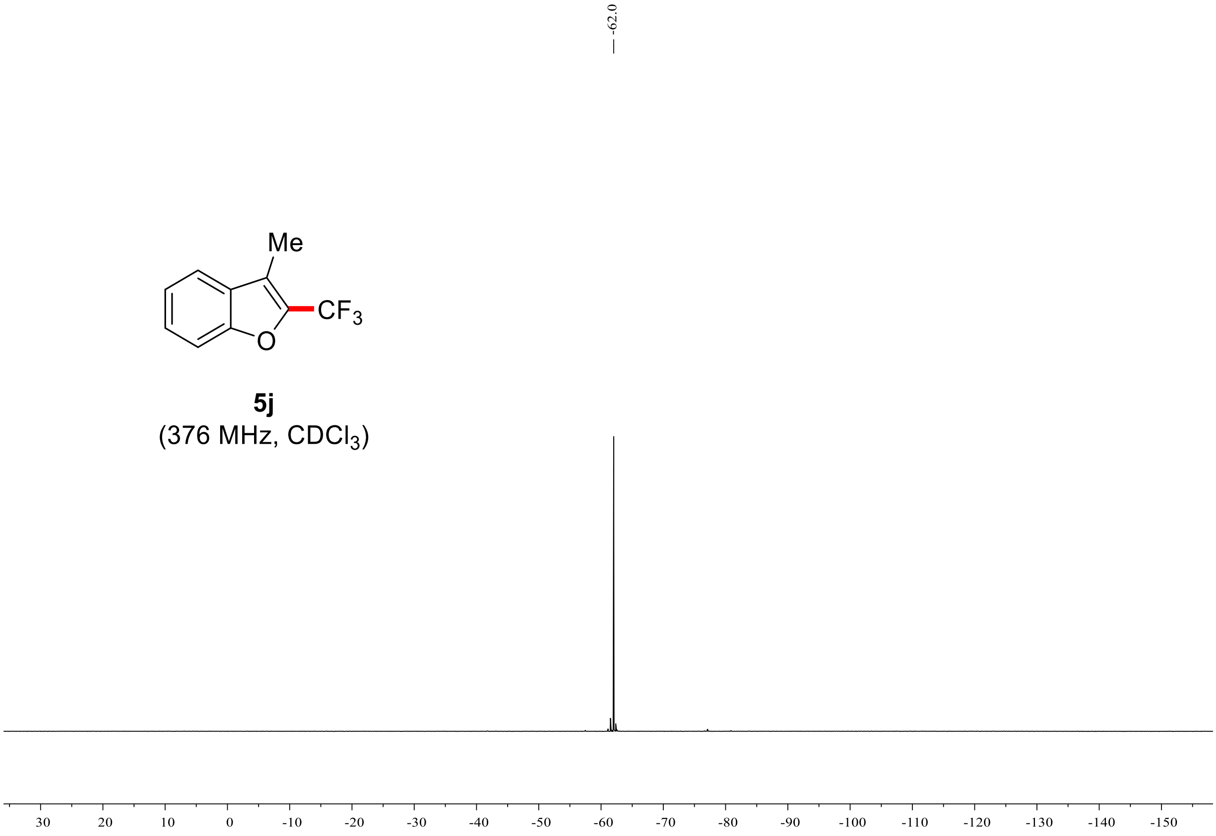
-62.0
Me
CF3
O
5j
(376 MHz, CDCl3)
30 20 10 0 -10 -20 -30 -40 -50 -60 -70 -80 -90 -100 -110 -120 -130 -140 -150

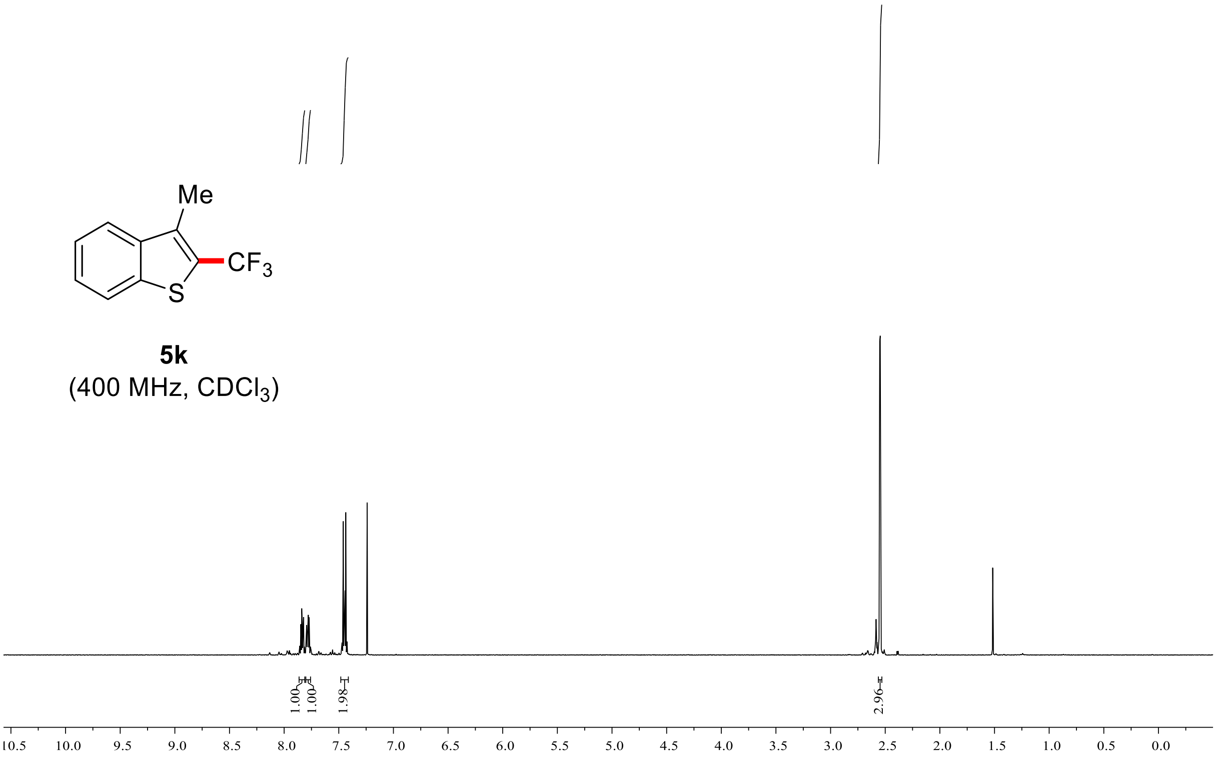
Me
CF3
S
5k
(400 MHz, CDCl3)
1.00
1.00
1.98
2.96
10.5 10.0 9.5 9.0 8.5 8.0 7.5 7.0 6.5 6.0 5.5 5.0 4.5 4.0 3.5 3.0 2.5 2.0 1.5 1.0 0.5 0.0

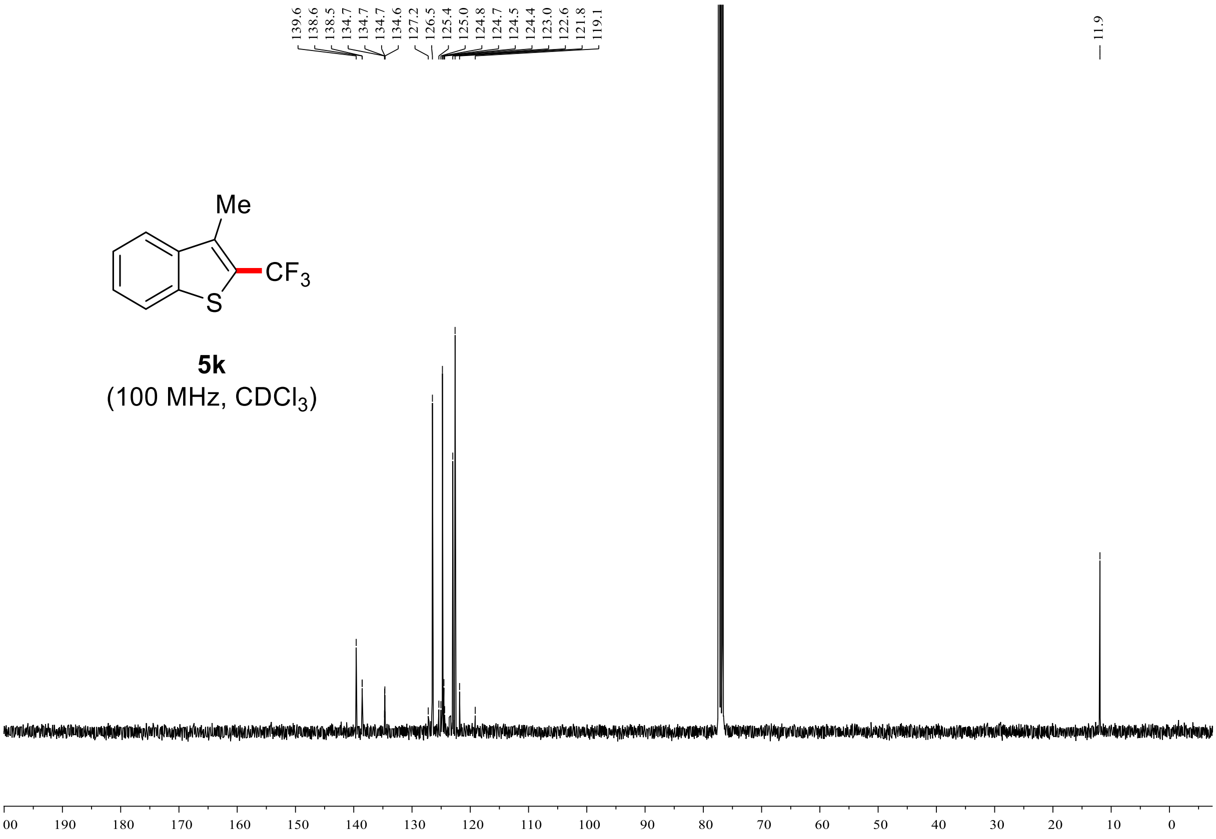
139.6
138.6
138.5
134.7
134.7
134.7
134.6
127.2
126.5
125.4
125.0
124.8
124.7
124.5
124.4
123.0
122.6
121.8
119.1
11.9
Me
CF3
S
5k
(100 MHz, CDCl3)
00 190 180 170 160 150 140 130 120 110 100 90 80 70 60 50 40 30 20 10 0

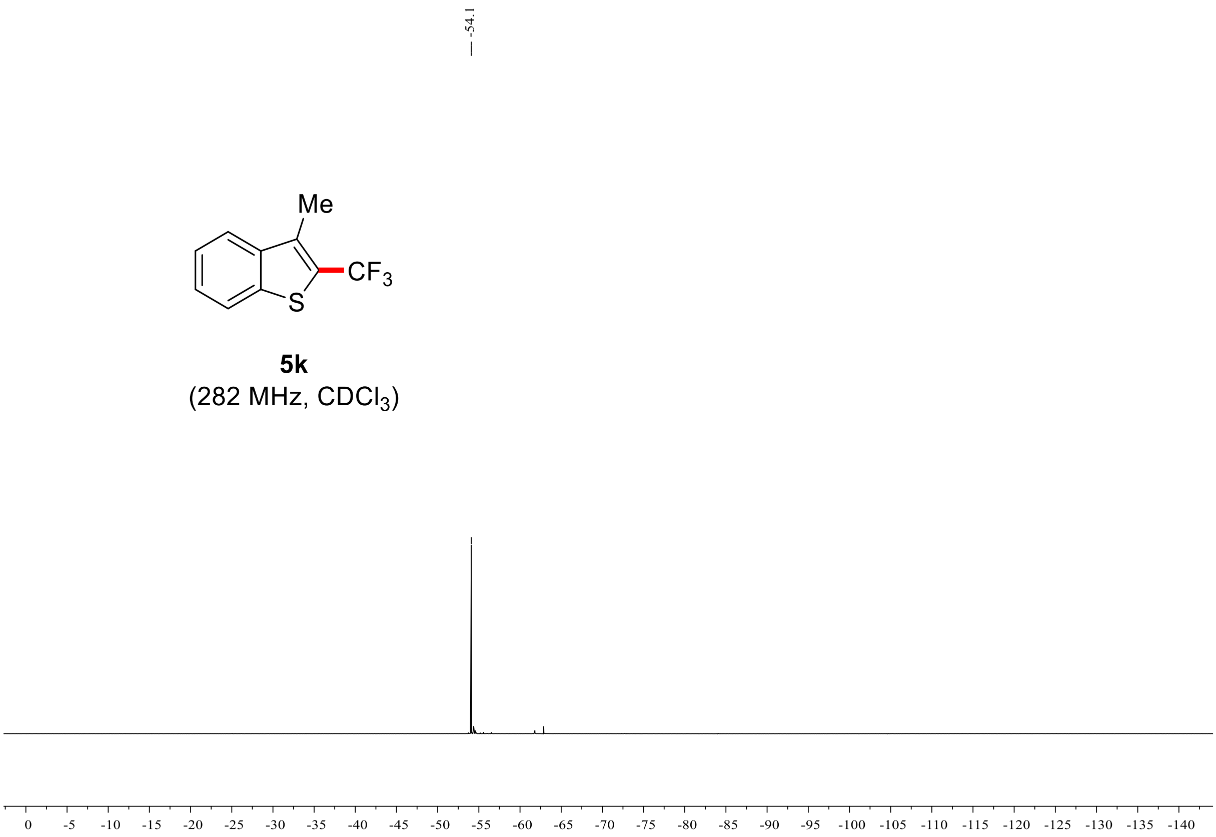
-54.1
Me
CF3
S
5k
(282 MHz, CDCl3)
0 -5 -10 -15 -20 -25 -30 -35 -40 -45 -50 -55 -60 -65 -70 -75 -80 -85 -90 -95 -100 -105 -110 -115 -120 -125 -130 -135 -140

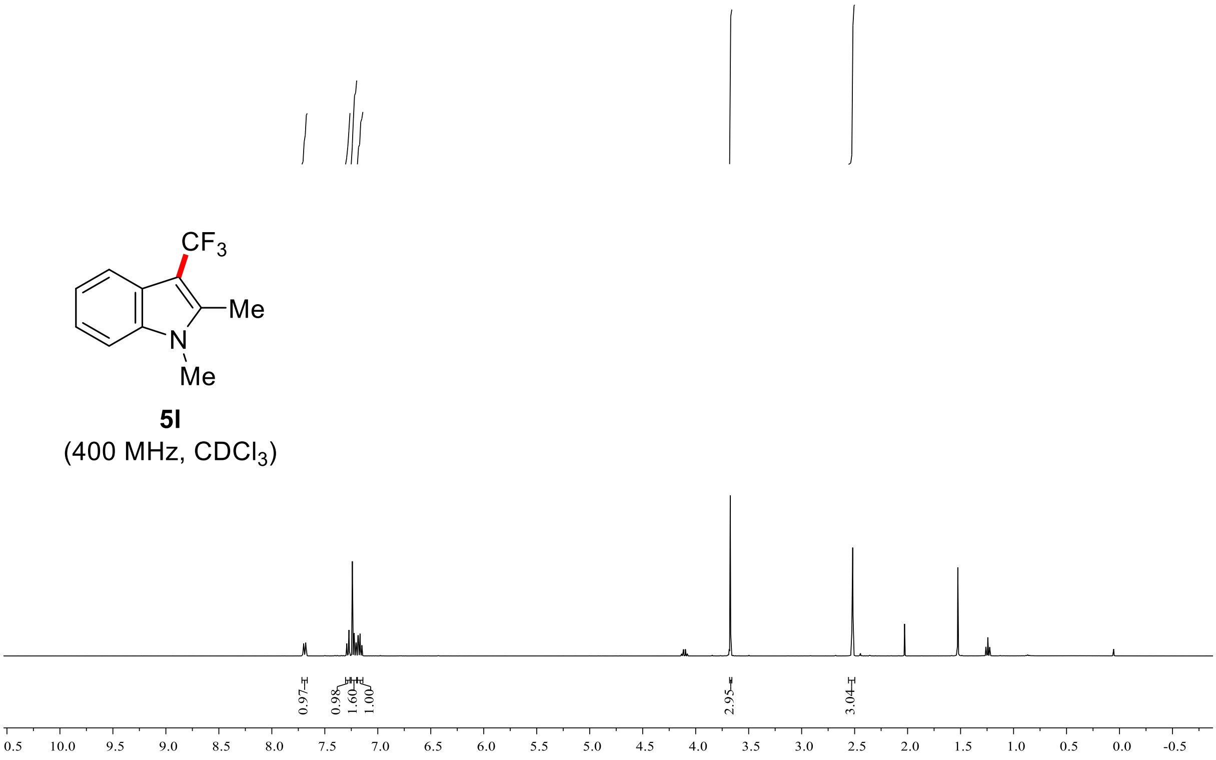
CF3
Me
N
Me
5l
(400 MHz, CDCl3)
0.97
0.98
1.60
1.00
2.95
3.04
0.5 10.0 9.5 9.0 8.5 8.0 7.5 7.0 6.5 6.0 5.5 5.0 4.5 4.0 3.5 3.0 2.5 2.0 1.5 1.0 0.5 0.0 -0.5

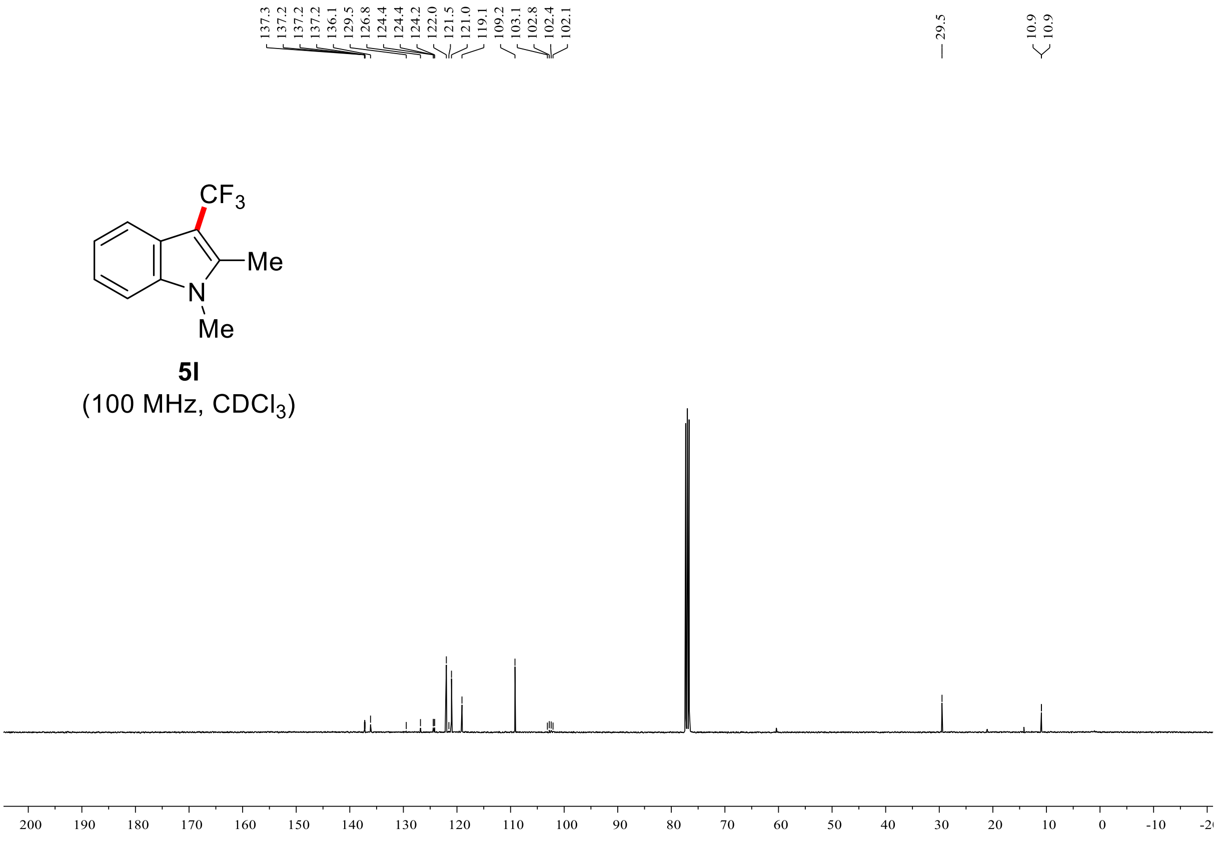
137.3
137.2
137.2
137.2
136.1
129.5
126.8
124.4
124.4
124.2
122.0
121.5
121.0
119.1
109.2
103.1
102.8
102.4
102.1
29.5
10.9
10.9
CF3
Me
N
Me
5l
(100 MHz, CDCl3)
200 190 180 170 160 150 140 130 120 110 100 90 80 70 60 50 40 30 20 10 0 -10

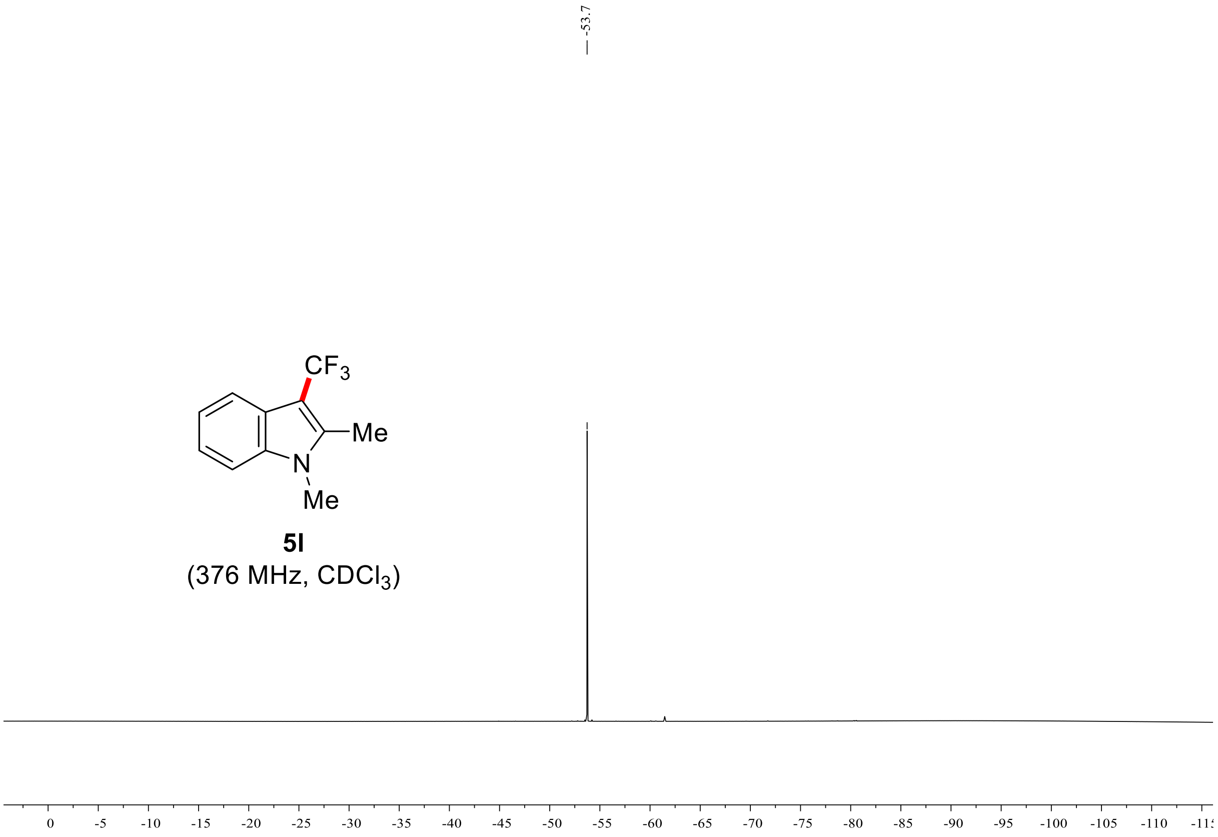
-53.7
CF3
Me
N
Me
5l
(376 MHz, CDCl3)
0 -5 -10 -15 -20 -25 -30 -35 -40 -45 -50 -55 -60 -65 -70 -75 -80 -85 -90 -95 -100 -105 -110

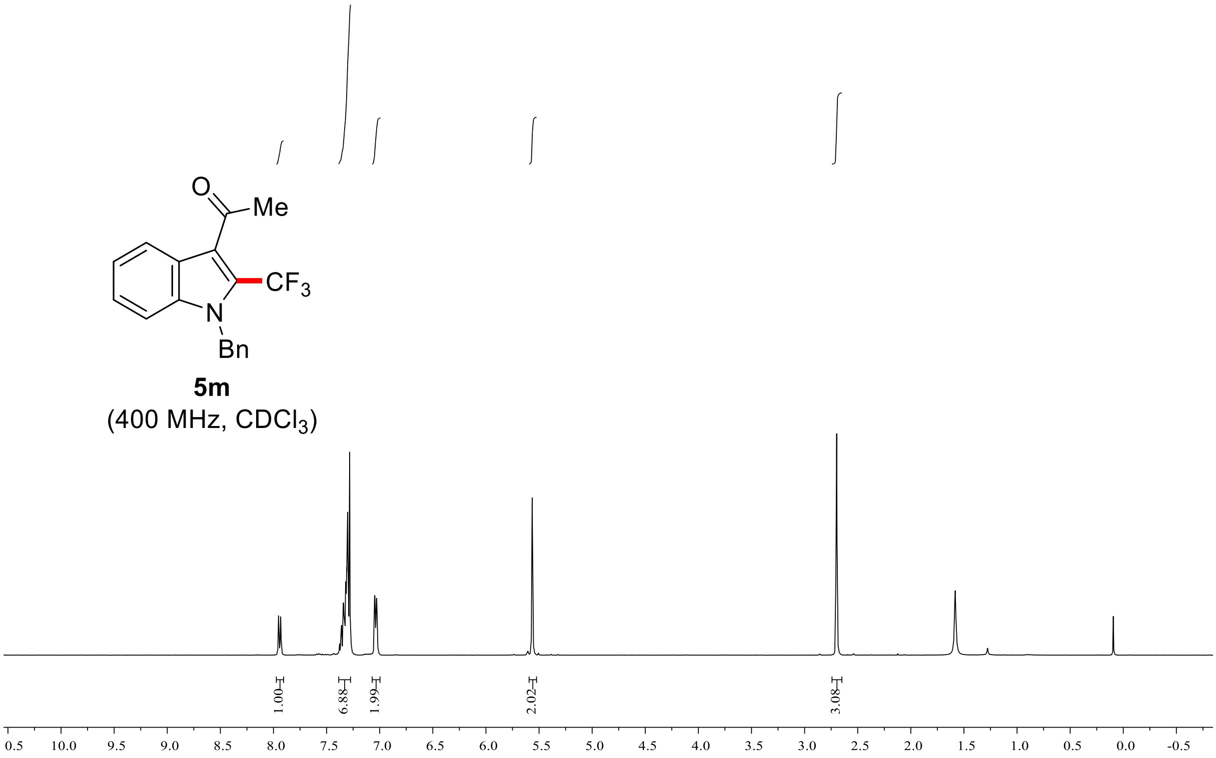
O
Me
CF3
N
Bn
5m
(400 MHz, CDCl3)
1.00
6.88
1.99
2.02
3.08
0.5 10.0 9.5 9.0 8.5 8.0 7.5 7.0 6.5 6.0 5.5 5.0 4.5 4.0 3.5 3.0 2.5 2.0 1.5 1.0 0.5 0.0 -0.5

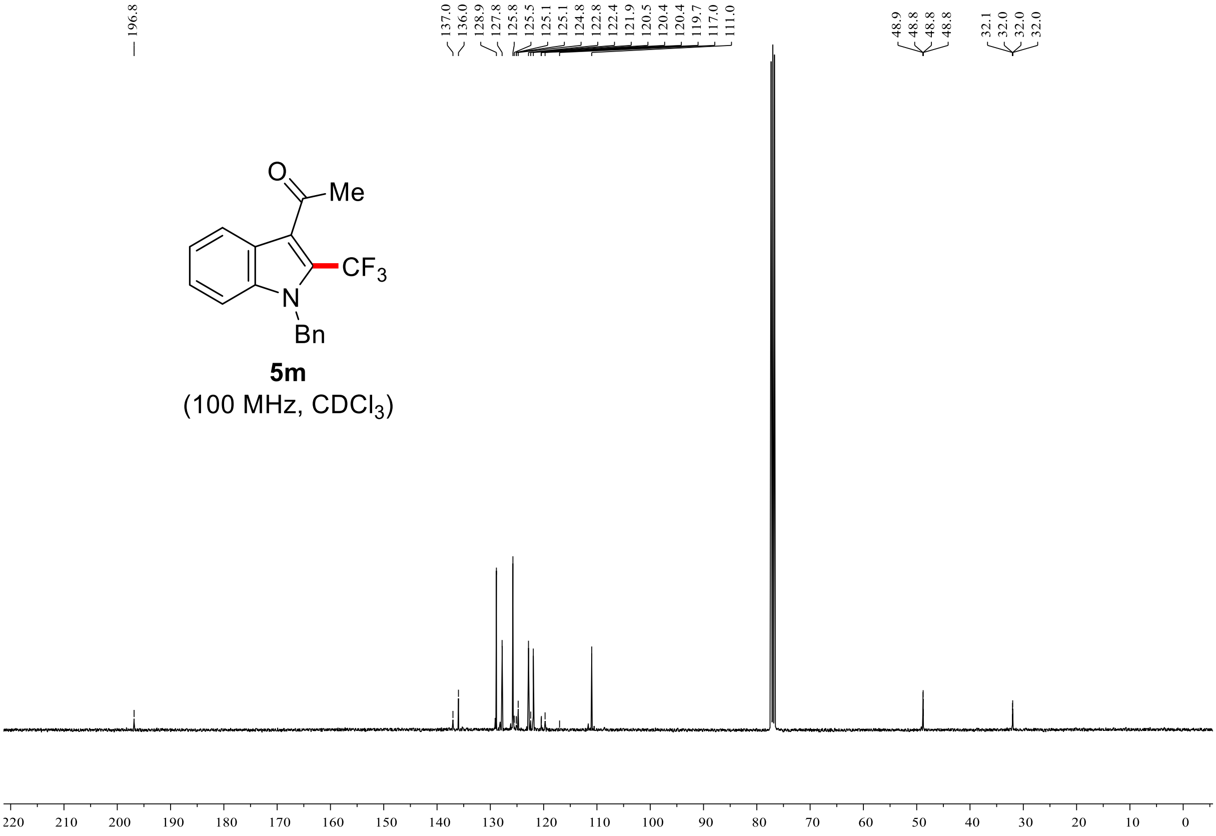
196.8
137.0
136.0
128.9
127.8
125.8
125.5
125.1
125.1
124.8
122.8
122.4
121.9
120.5
120.4
120.4
119.7
117.0
111.0
48.9
48.8
48.8
48.8
32.1
32.0
32.0
32.0
O
Me
CF3
N
Bn
5m
(100 MHz, CDCl3)
220 210 200 190 180 170 160 150 140 130 120 110 100 90 80 70 60 50 40 30 20 10 0

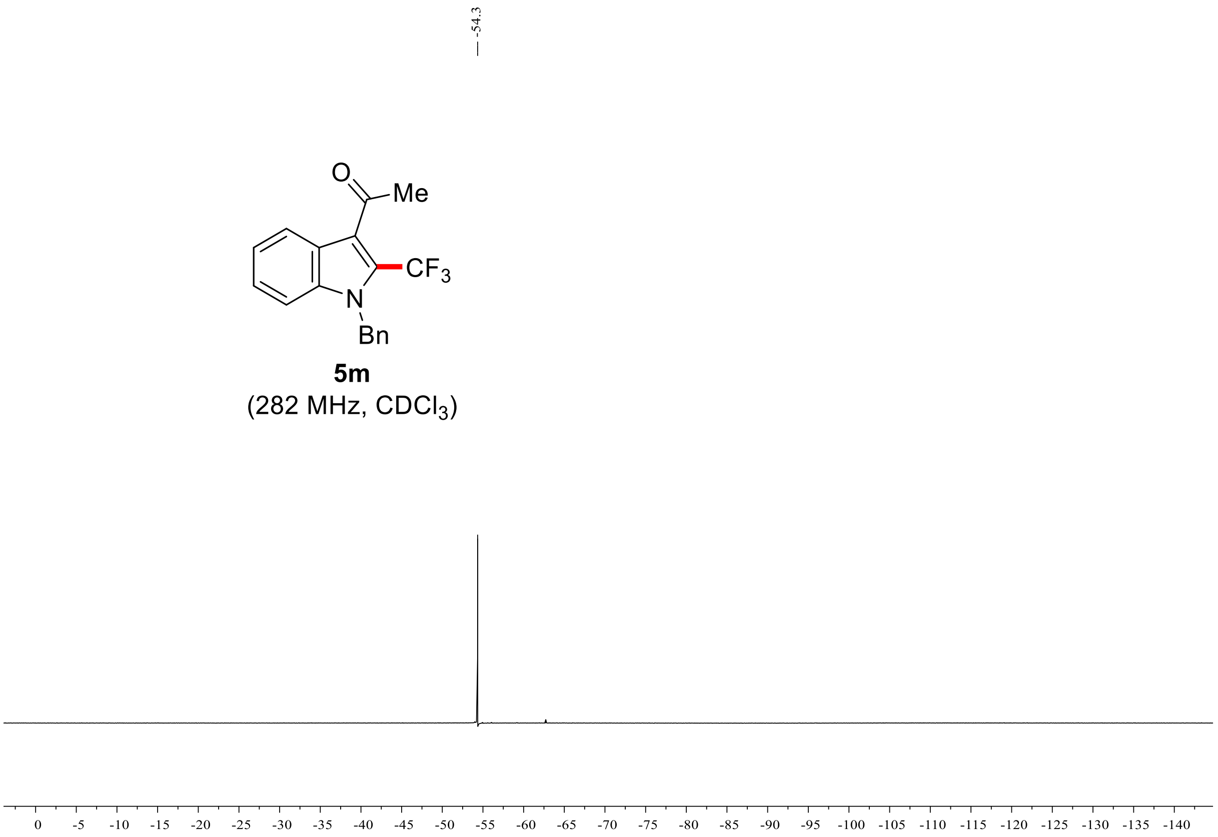

5m
(282 MHz, $CDCl_3$)

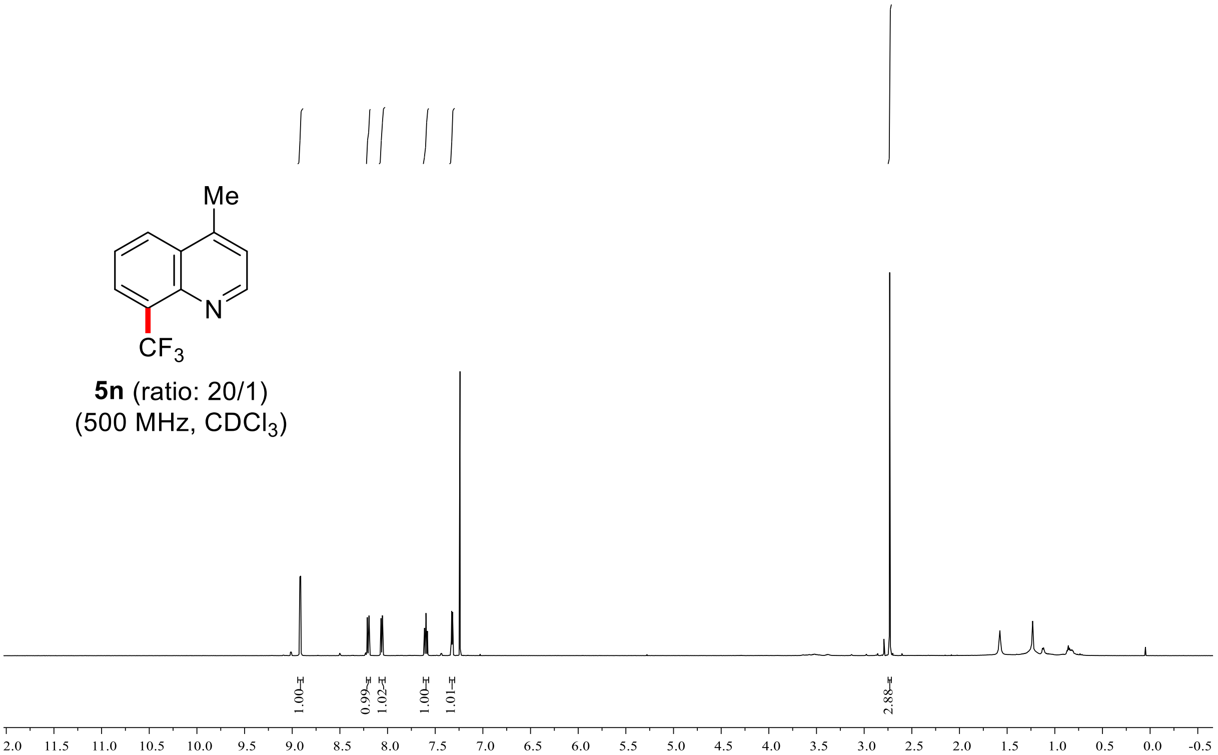

5n (ratio: 20/1)
(500 MHz, $CDCl_3$)

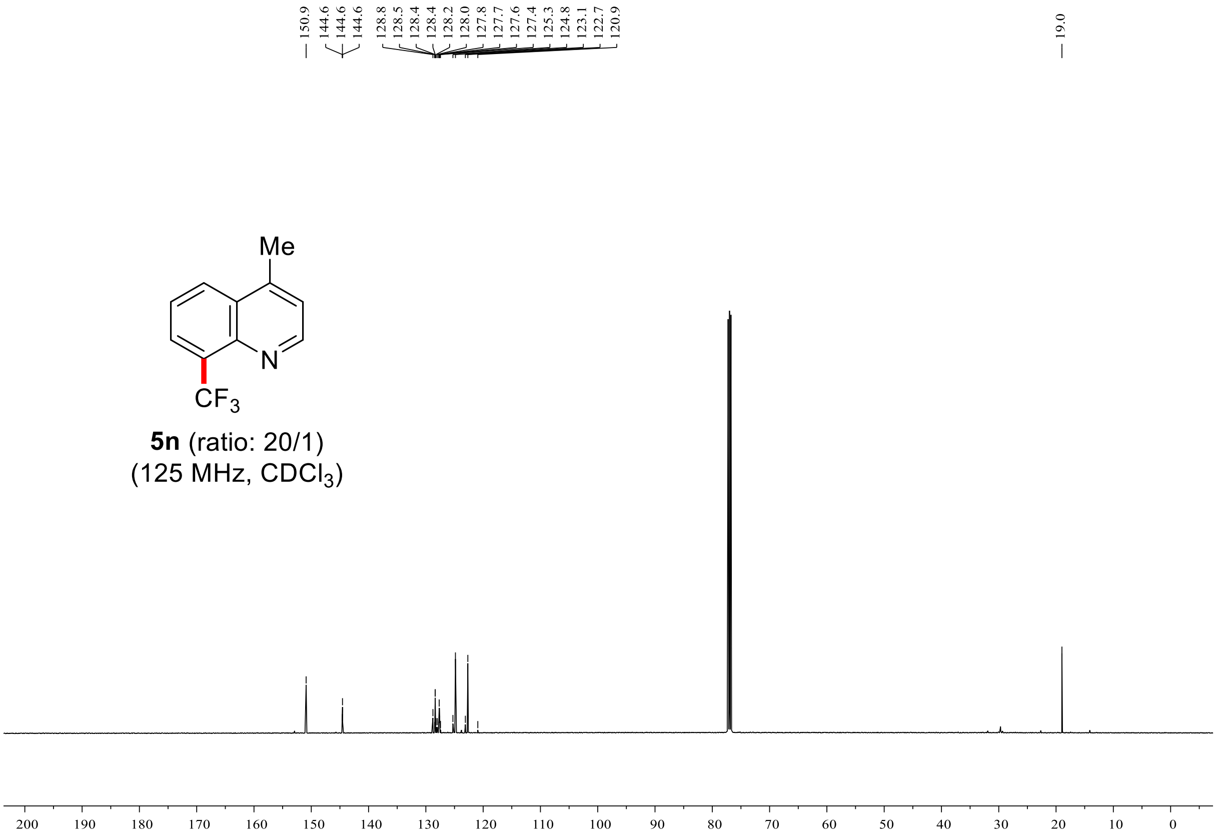

5n (ratio: 20/1)
(125 MHz, $CDCl_3$)

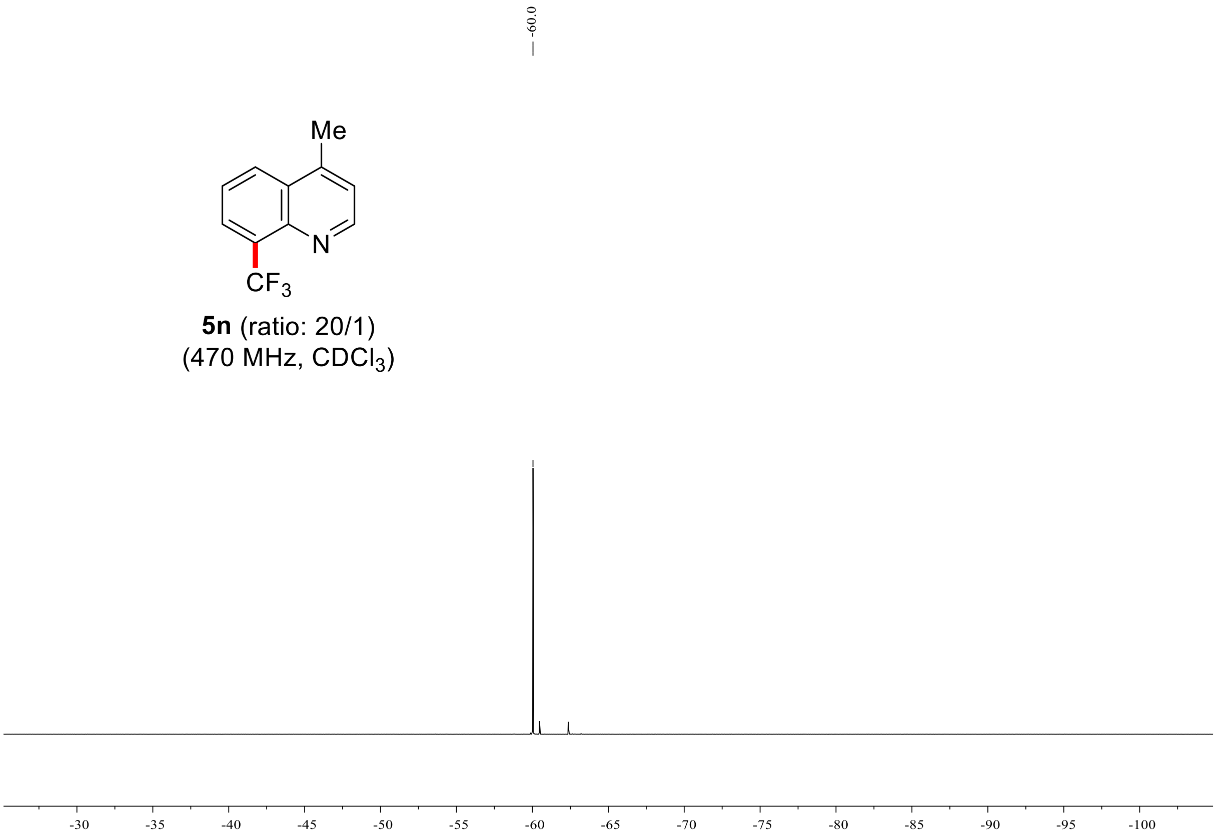

5n (ratio: 20/1)
(470 MHz, $CDCl_3$)

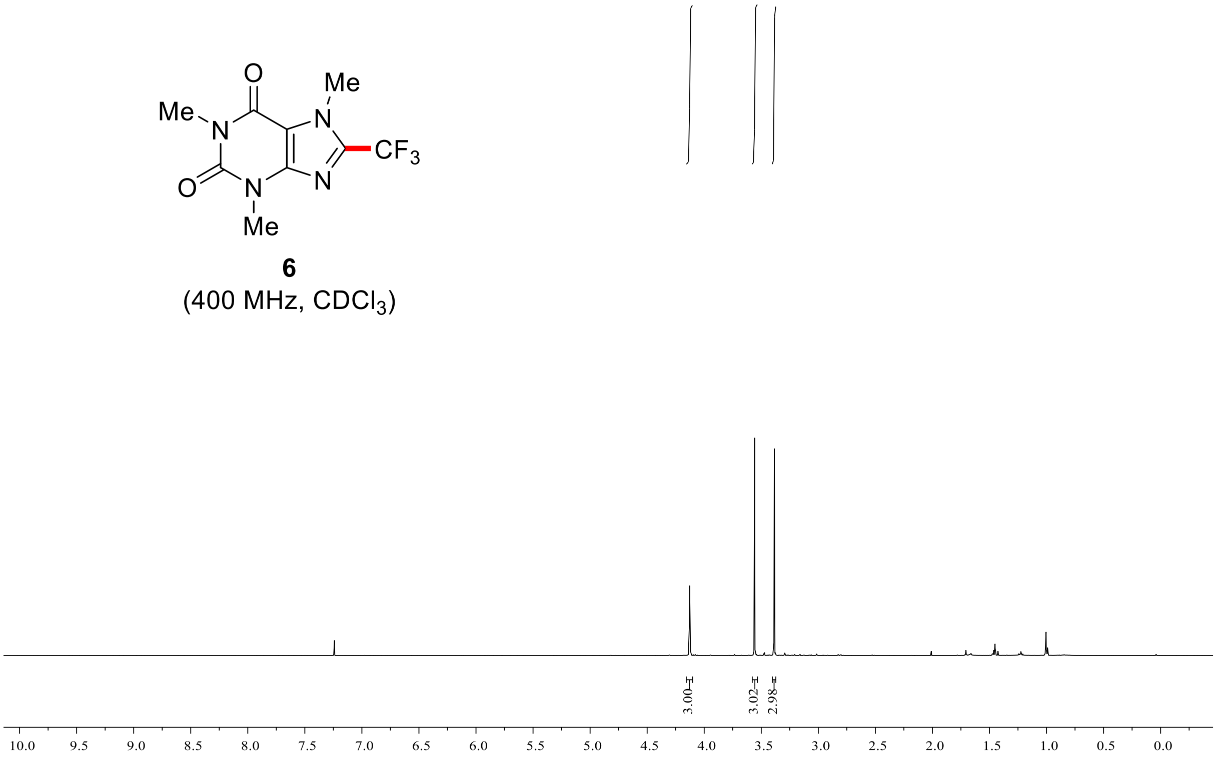
Me
O
Me
N
N
CF3
O
N
N
Me
6
(400 MHz, CDCl3)
3.00
3.02
2.98
10.0 9.5 9.0 8.5 8.0 7.5 7.0 6.5 6.0 5.5 5.0 4.5 4.0 3.5 3.0 2.5 2.0 1.5 1.0 0.5 0.0

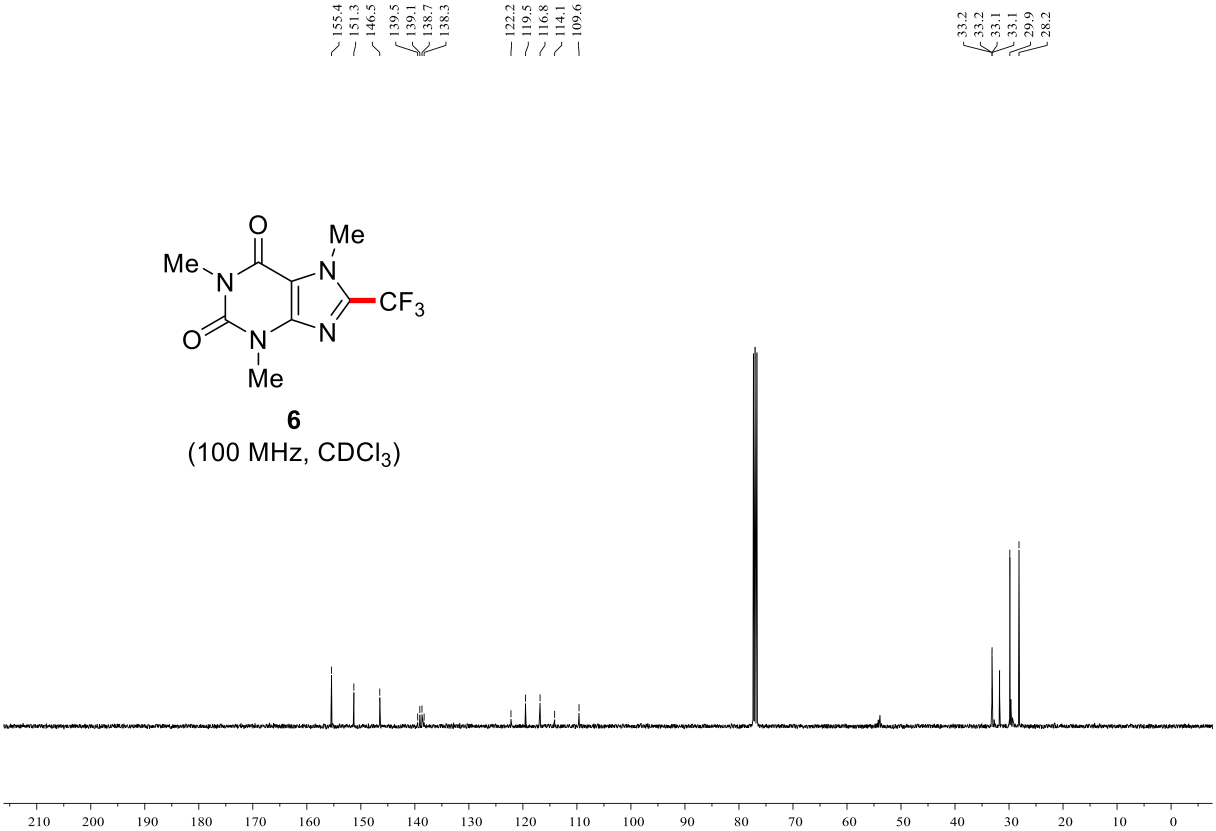
155.4
151.3
146.5
139.5
139.1
138.7
138.3
122.2
119.5
116.8
114.1
109.6
33.2
33.2
33.1
33.1
29.9
28.2
Me
O
Me
N
N
CF3
O
N
N
Me
6
(100 MHz, CDCl3)
210 200 190 180 170 160 150 140 130 120 110 100 90 80 70 60 50 40 30 20 10 0

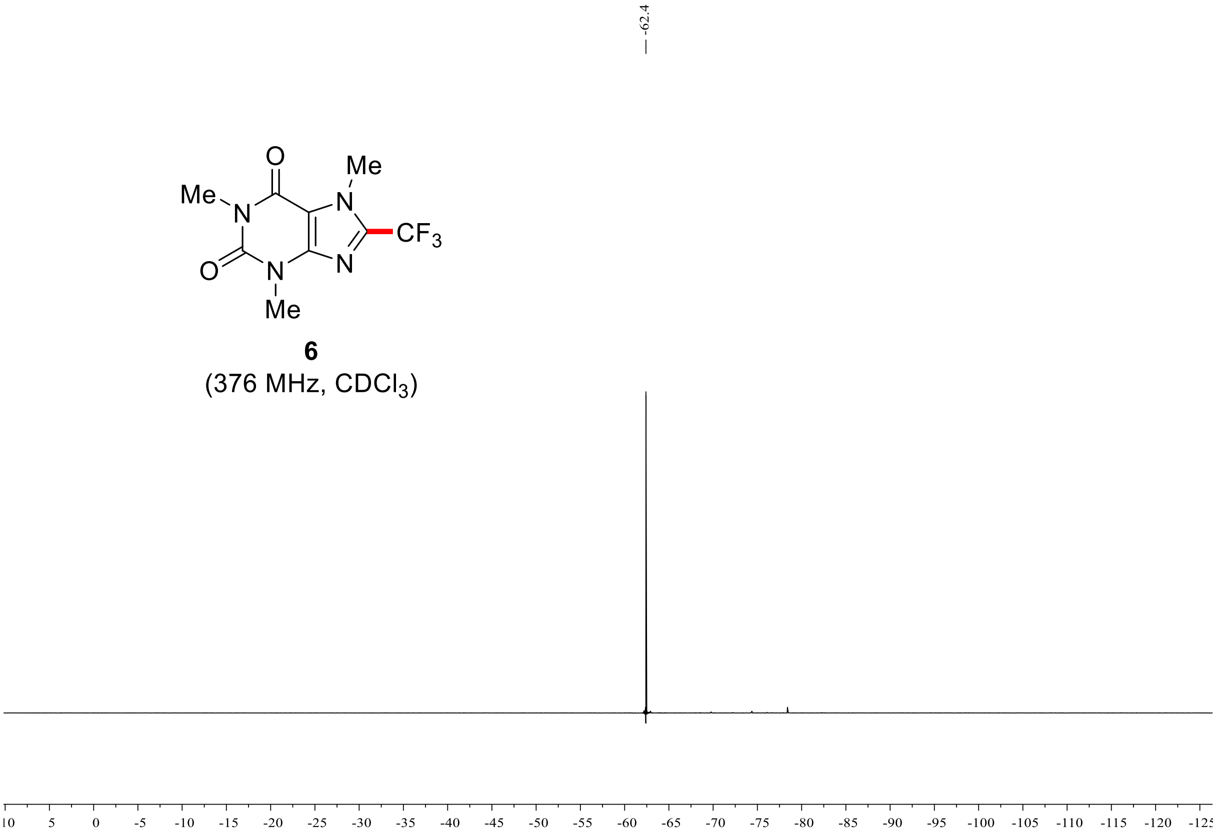

6
(376 MHz, $CDCl_3$)

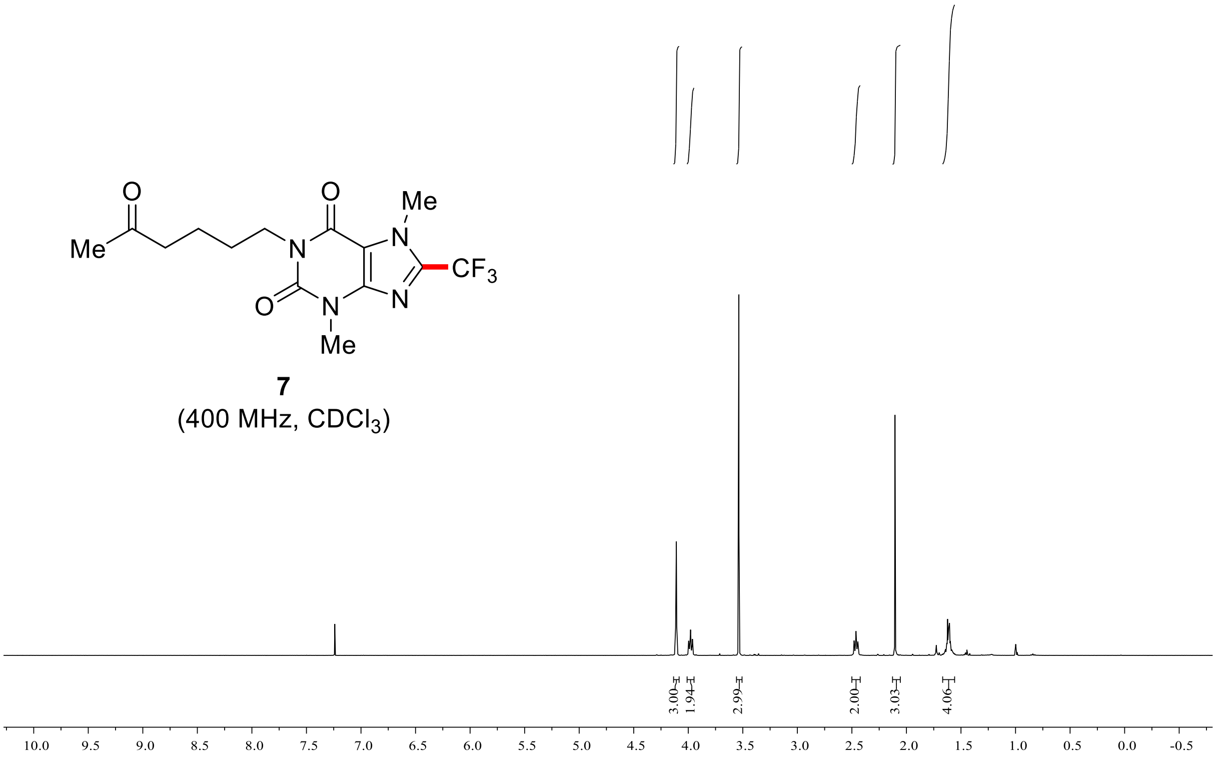

7
(400 MHz, $CDCl_3$)

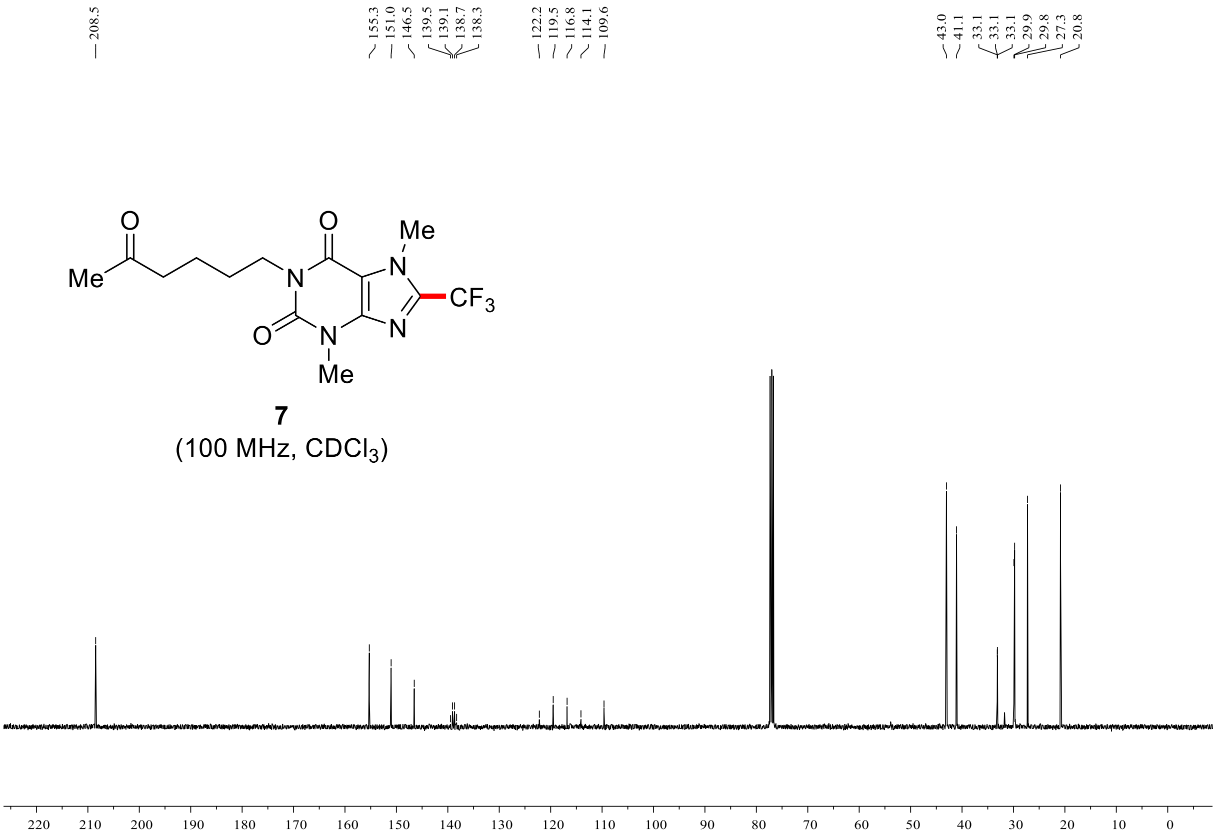

7
(100 MHz, $CDCl_3$)

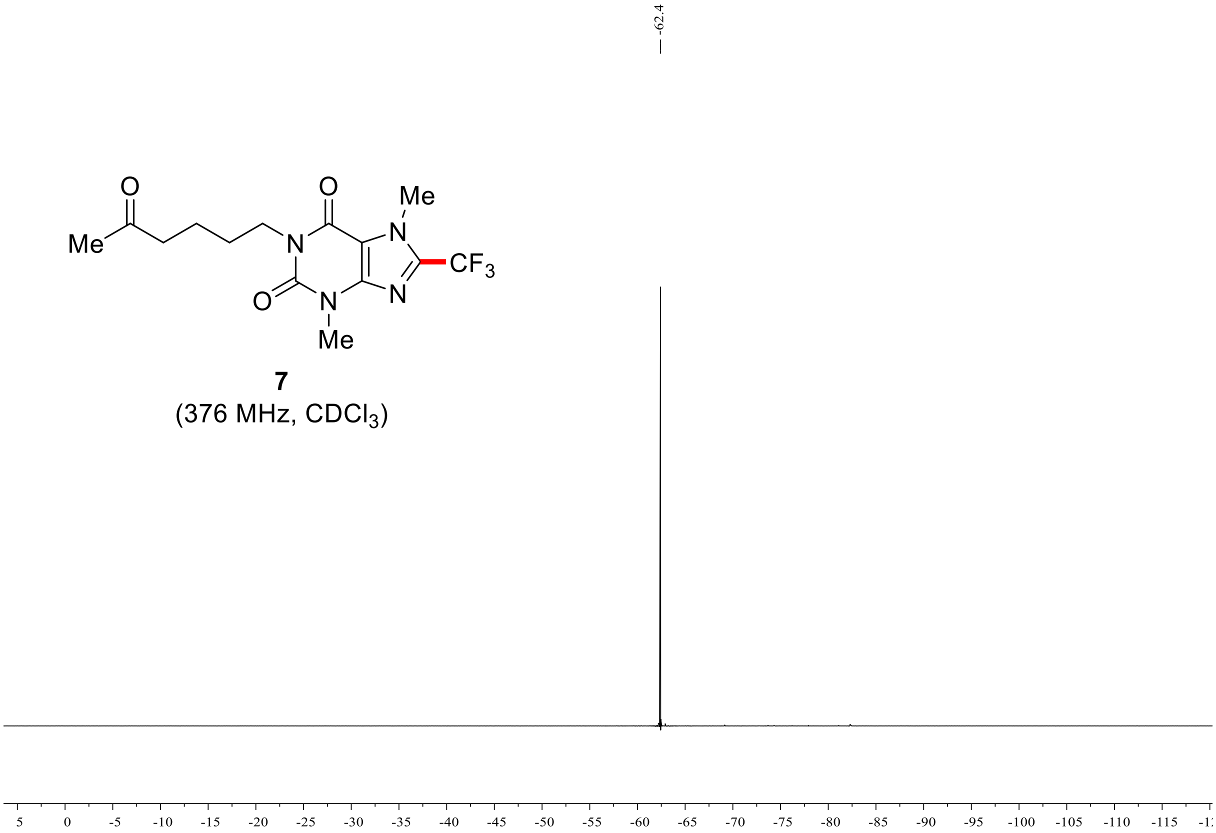

7
(376 MHz, $CDCl_3$)

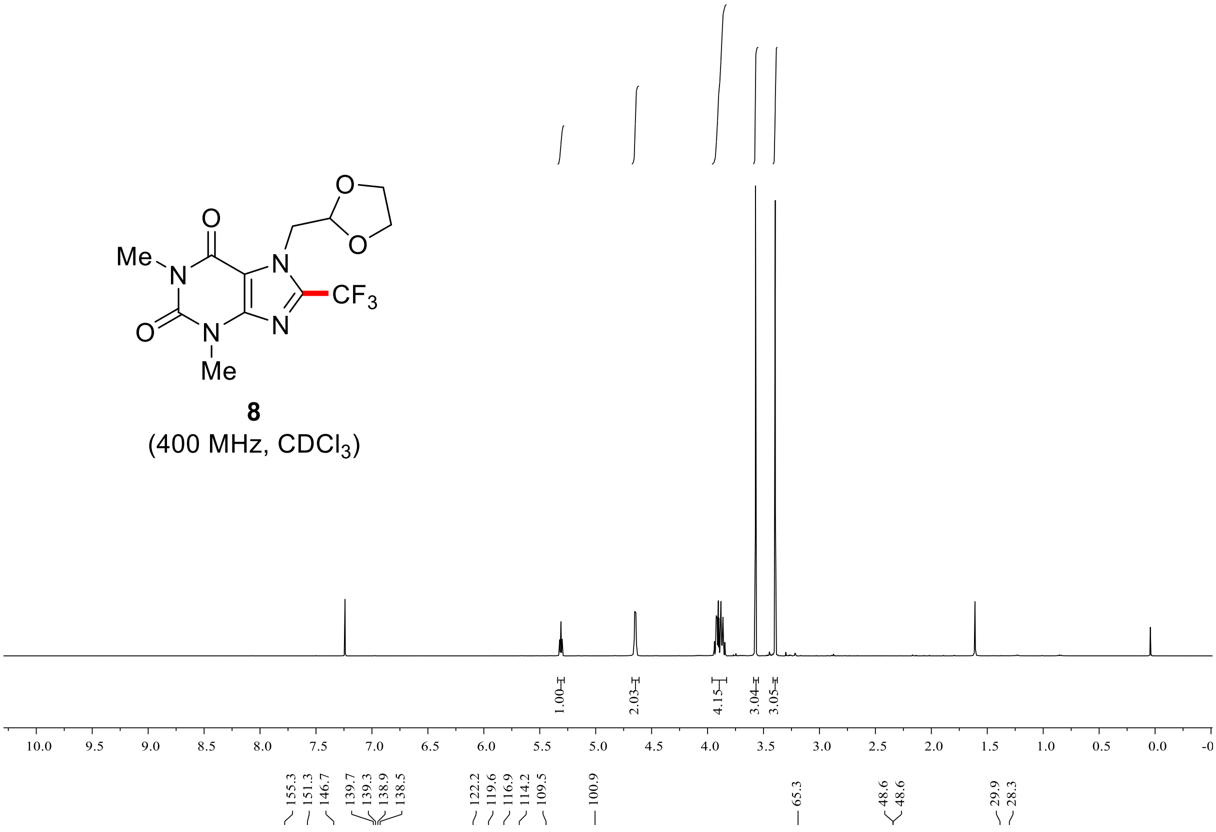

Me
N
O
N
Me
O
N
N
CF3
O
O
8
(400 MHz, CDCl3)
1.00
2.03
4.15
3.04
3.05
10.0 9.5 9.0 8.5 8.0 7.5 7.0 6.5 6.0 5.5 5.0 4.5 4.0 3.5 3.0 2.5 2.0 1.5 1.0 0.5 0.0 -0
155.3 151.3 146.7 139.7 139.3 138.9 138.5 122.2 119.6 116.9 114.2 109.5 100.9 65.3 48.6 48.6 29.9 28.3

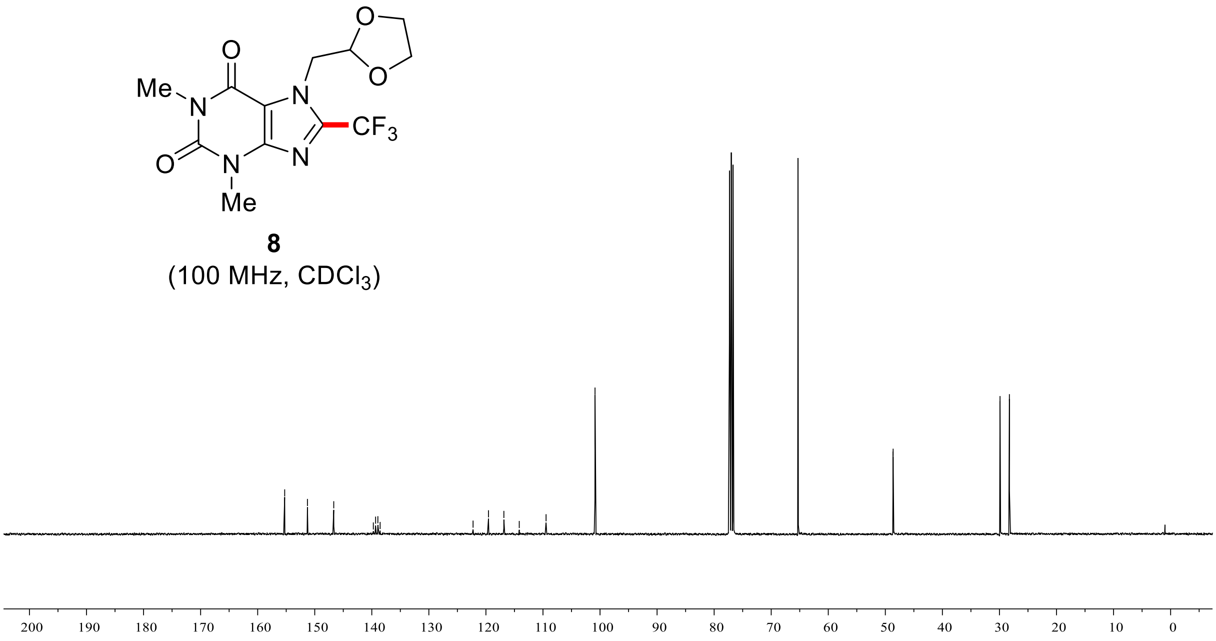

Me
N
O
N
Me
O
N
N
CF3
O
O
8
(100 MHz, CDCl3)
200 190 180 170 160 150 140 130 120 110 100 90 80 70 60 50 40 30 20 10 0

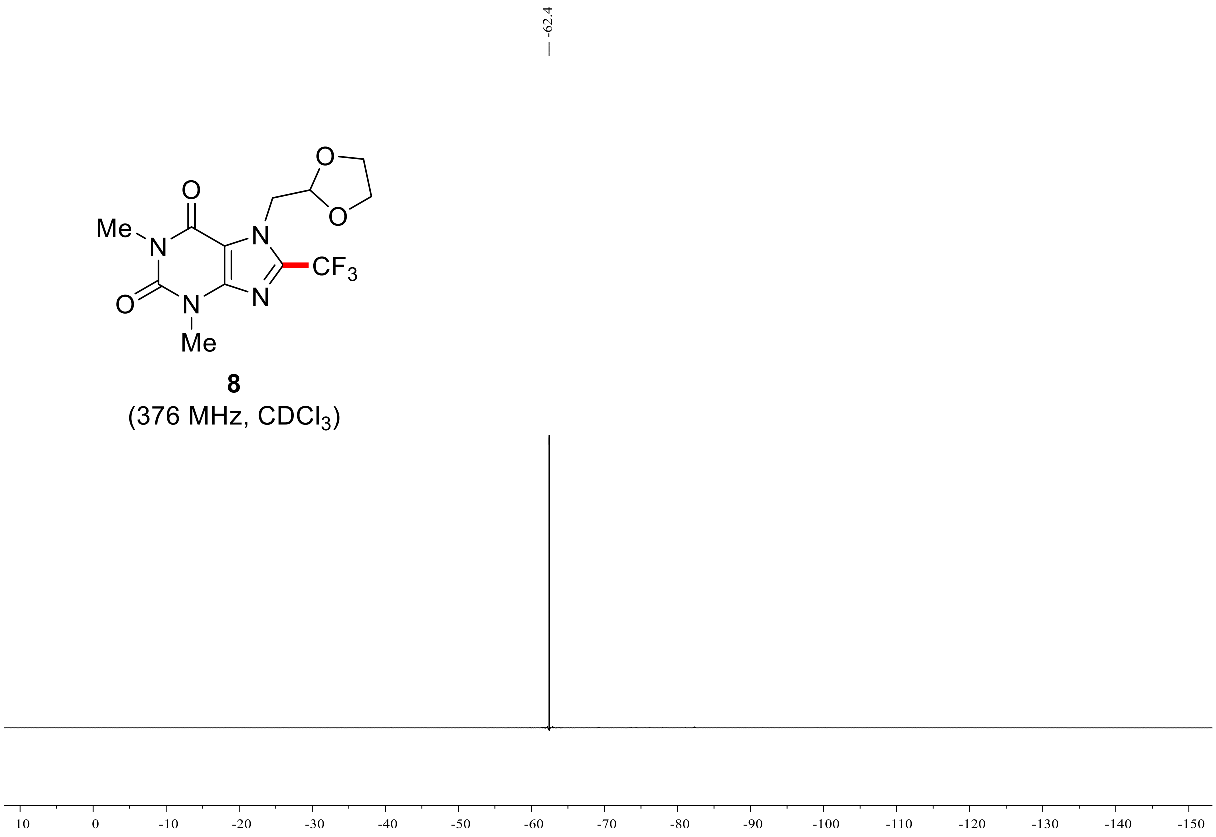
-62.4
Me
N
O
O
N
Me
N
N
CF3
O
O
8
(376 MHz, CDCl3)
10 0 -10 -20 -30 -40 -50 -60 -70 -80 -90 -100 -110 -120 -130 -140 -150

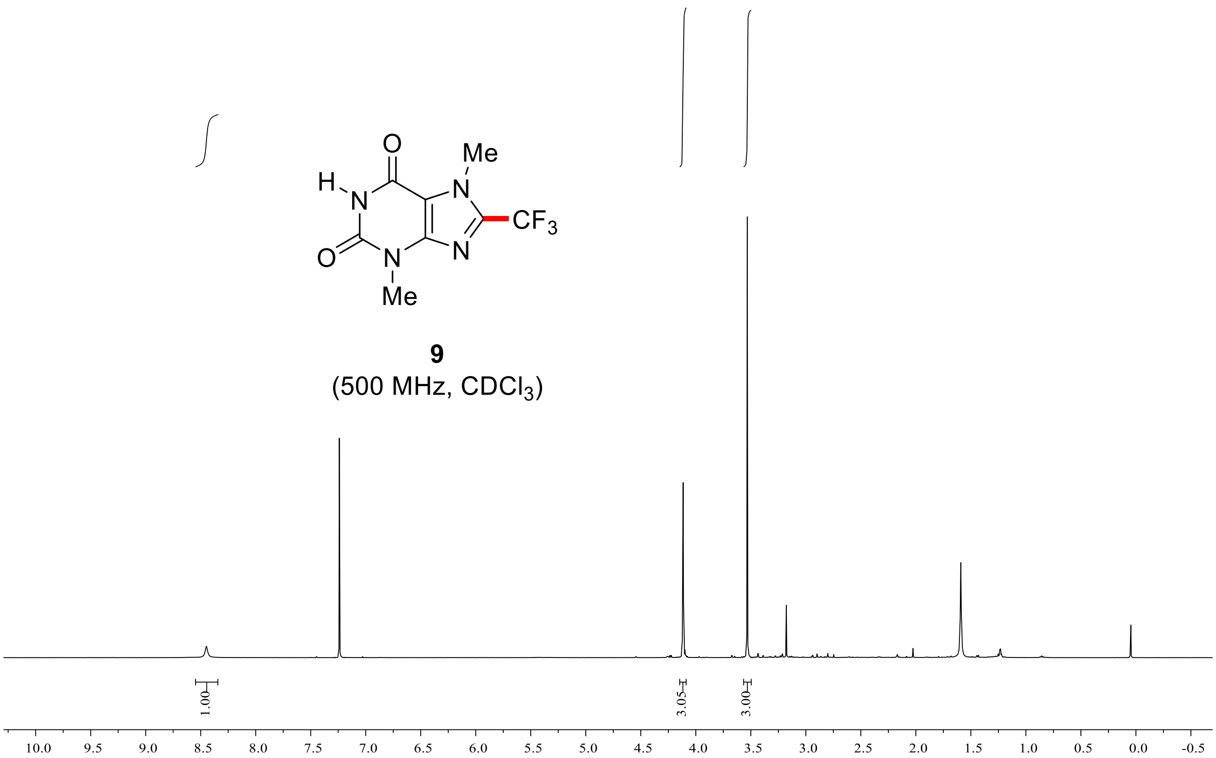
O
Me
H
N
N
CF3
O
N
N
Me
9
(500 MHz, CDCl3)
1.00
3.05
3.00
10.0 9.5 9.0 8.5 8.0 7.5 7.0 6.5 6.0 5.5 5.0 4.5 4.0 3.5 3.0 2.5 2.0 1.5 1.0 0.5 0.0 -0.5

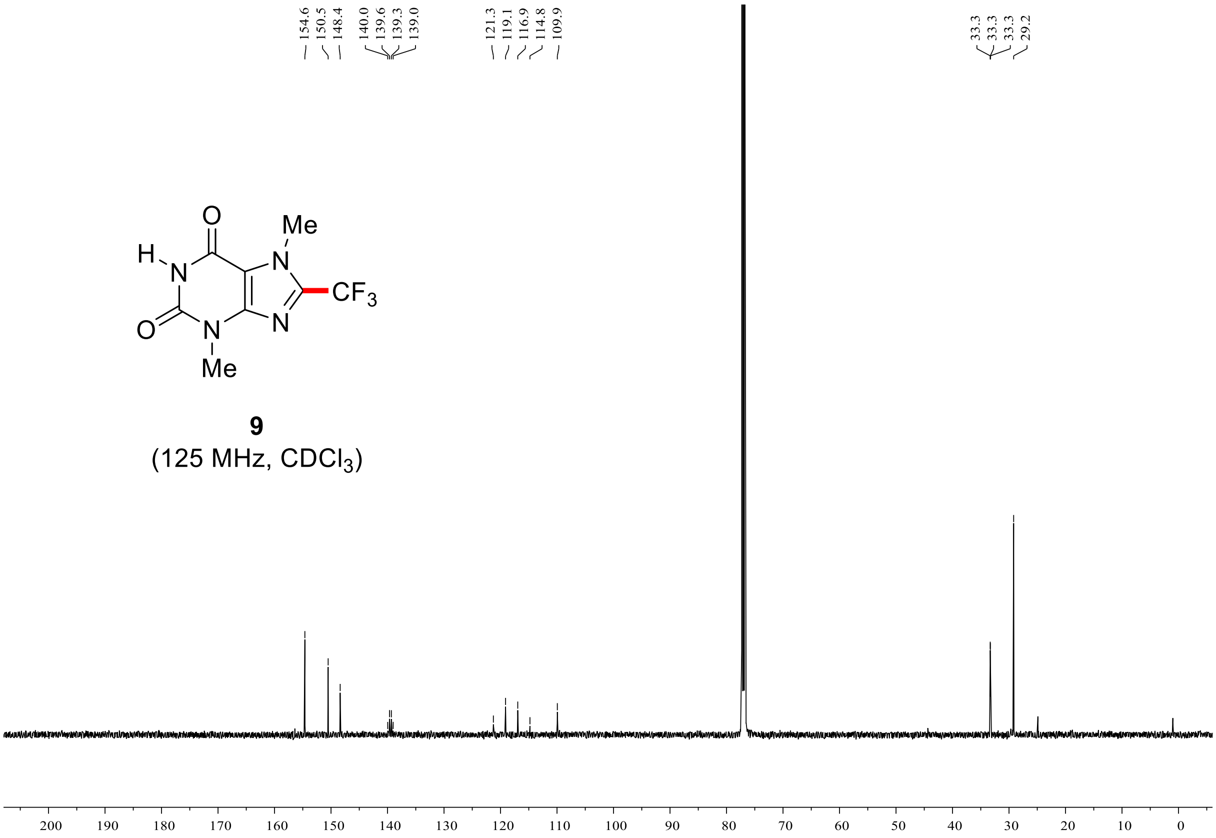

9
(125 MHz, $CDCl_3$)

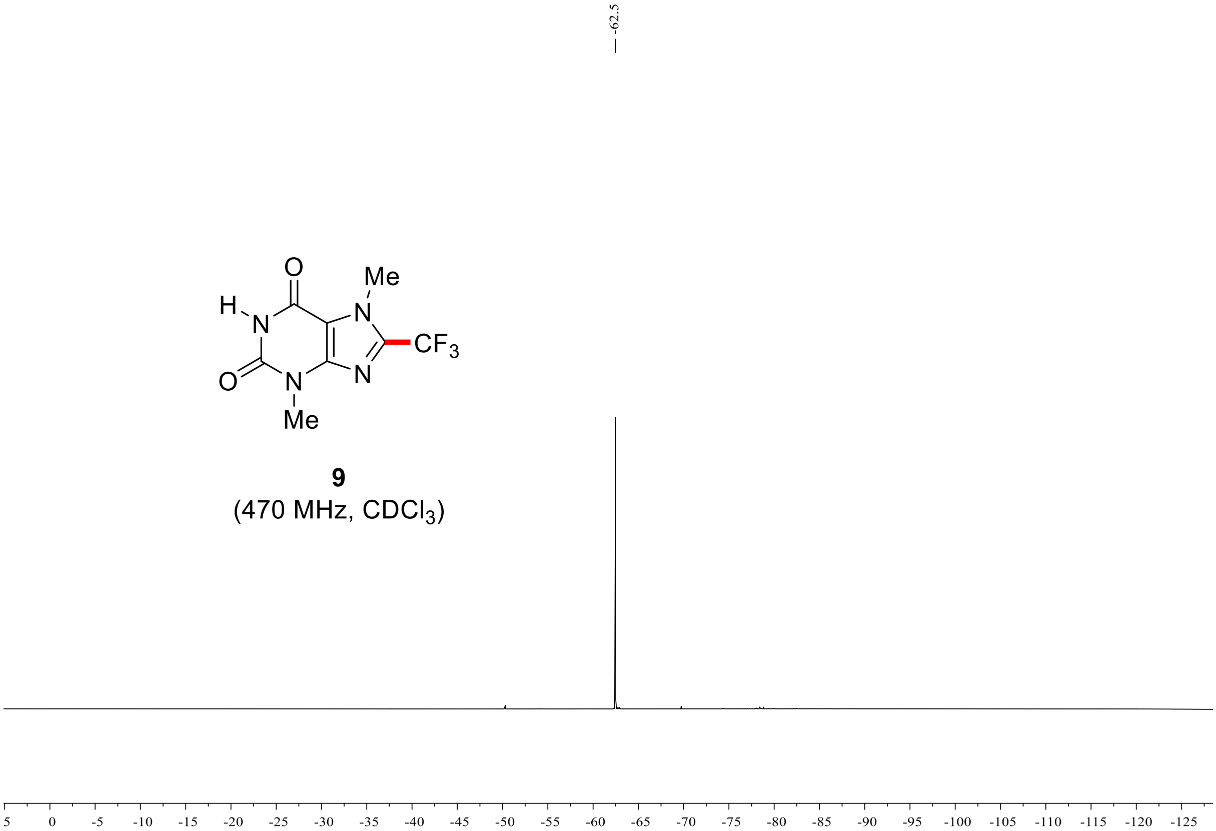

9
(470 MHz, $CDCl_3$)

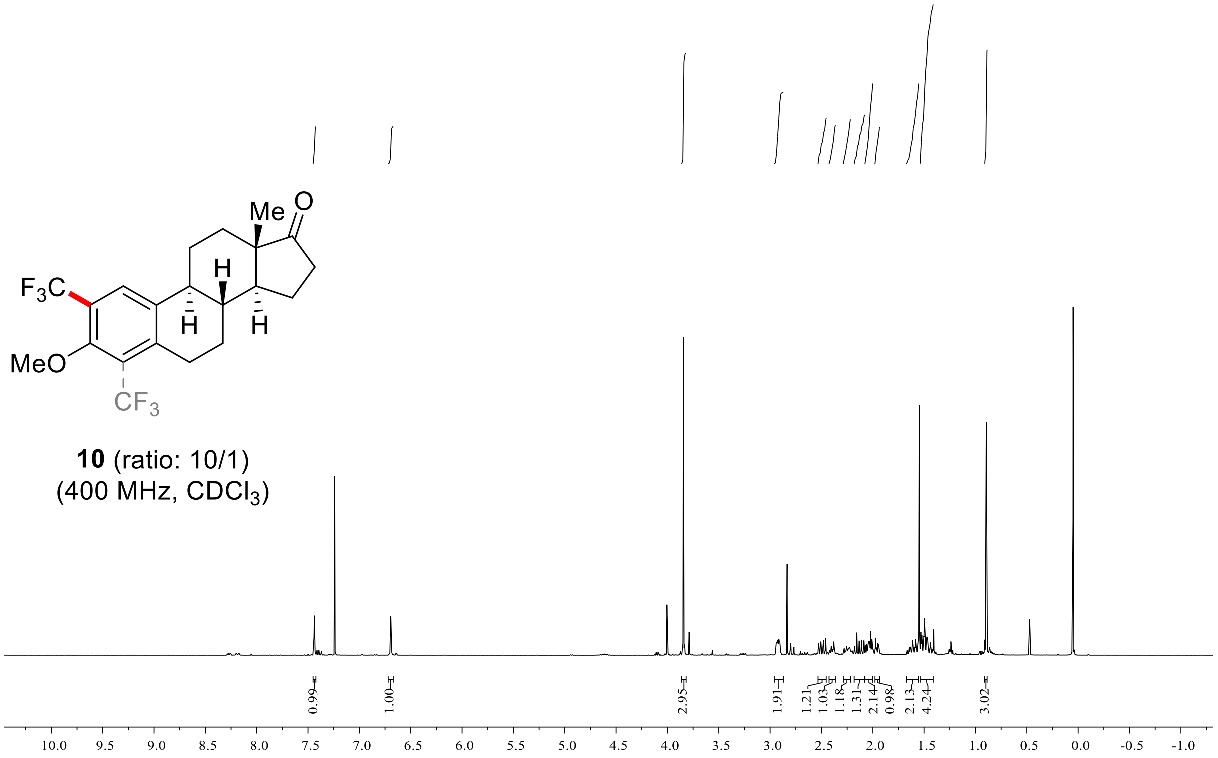

10 (ratio: 10/1)
(400 MHz, $CDCl_3$)

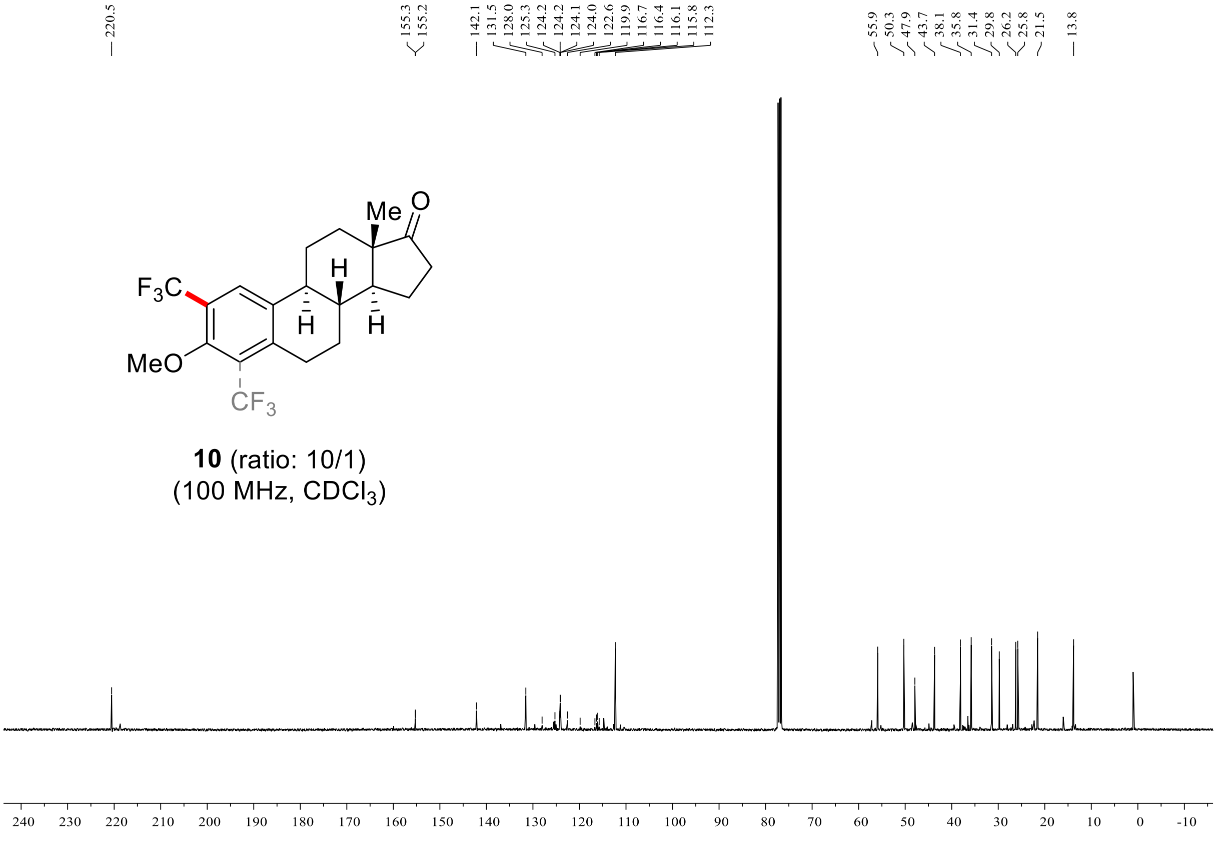

10 (ratio: 10/1)
(100 MHz, $CDCl_3$)

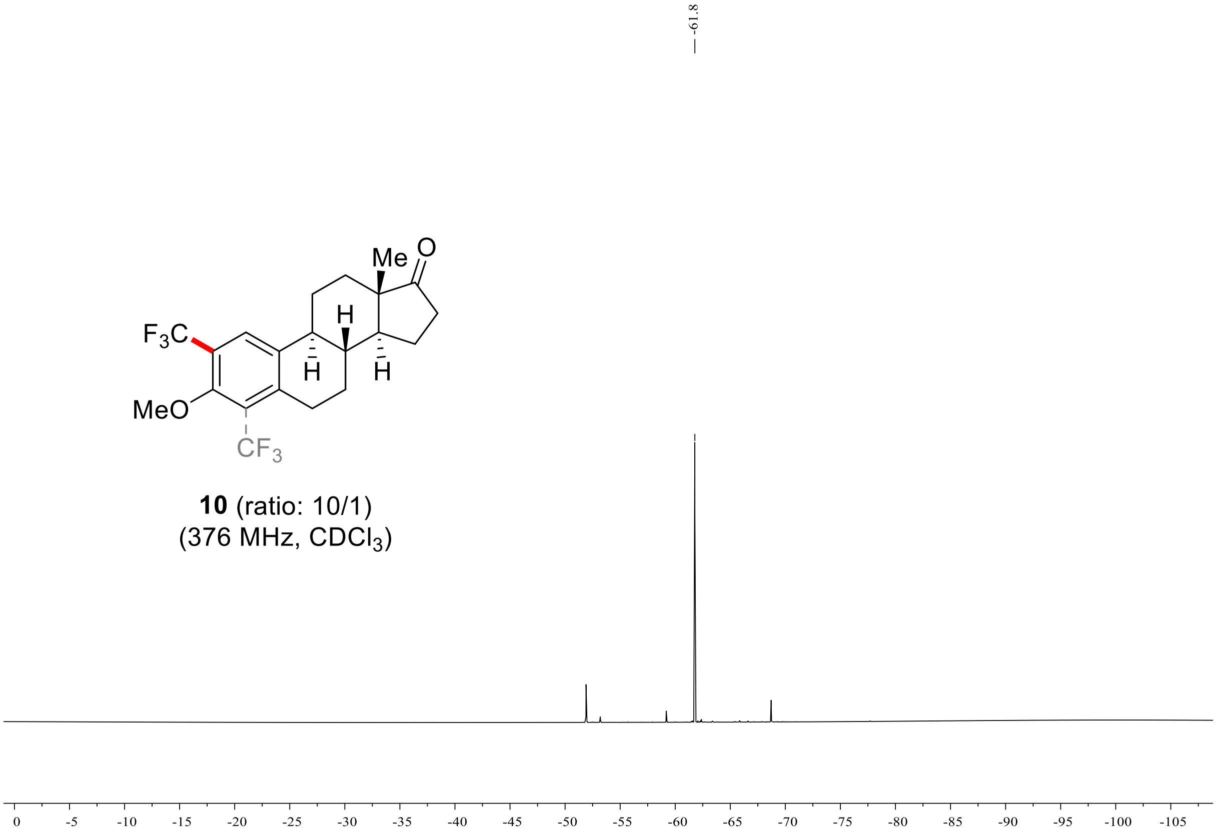

—61.8
F3C
MeO
CF3
Me
O
H
10 (ratio: 10/1)
(376 MHz, CDCl3)
0 -5 -10 -15 -20 -25 -30 -35 -40 -45 -50 -55 -60 -65 -70 -75 -80 -85 -90 -95 -100 -105

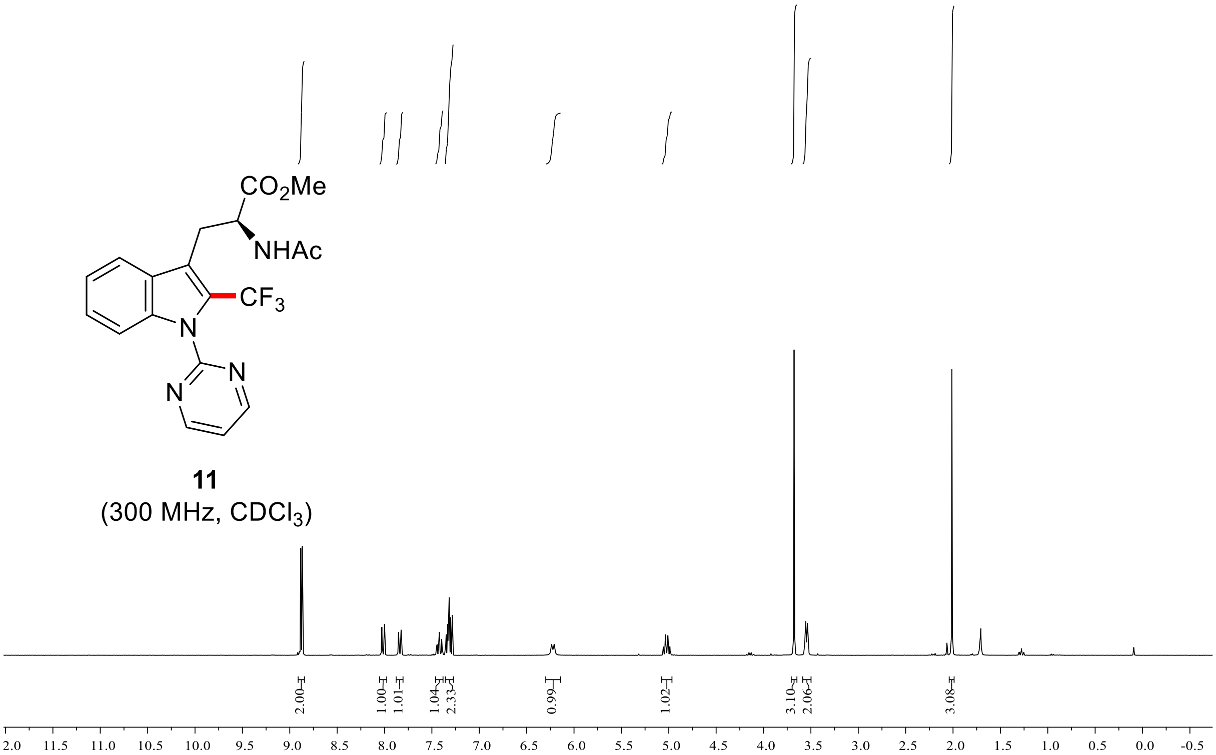

CO2Me
NHAc
CF3
N
11
(300 MHz, CDCl3)
2.00
1.00
1.01
1.04
2.33
0.99
1.02
3.10
2.06
3.08
2.0 11.5 11.0 10.5 10.0 9.5 9.0 8.5 8.0 7.5 7.0 6.5 6.0 5.5 5.0 4.5 4.0 3.5 3.0 2.5 2.0 1.5 1.0 0.5 0.0 -0.5

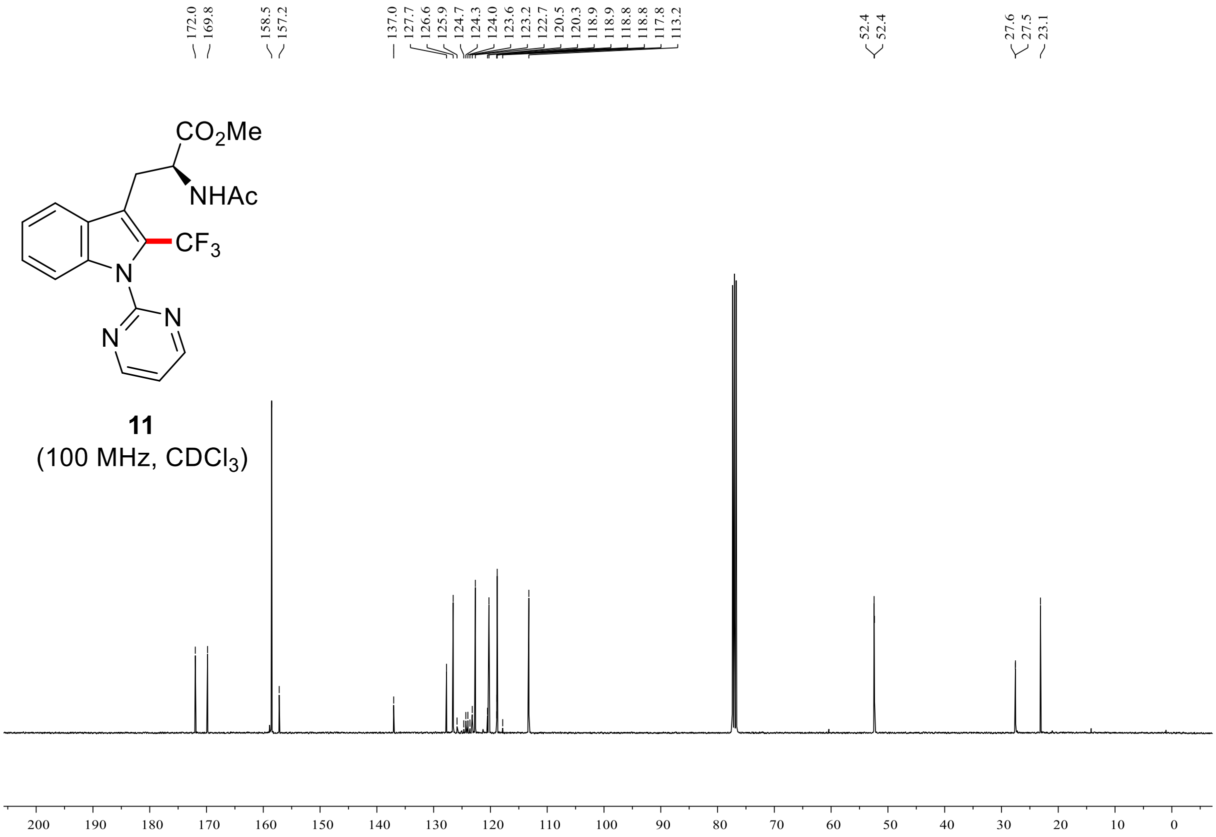
CO2Me
NHAc
CF3
N
N
N
11
(100 MHz, CDCl3)
172.0
169.8
158.5
157.2
137.0
127.7
126.6
125.9
124.7
124.3
124.0
123.6
123.2
122.7
120.5
120.3
118.9
118.9
118.8
118.8
117.8
113.2
52.4
52.4
27.6
27.5
23.1
200 190 180 170 160 150 140 130 120 110 100 90 80 70 60 50 40 30 20 10 0

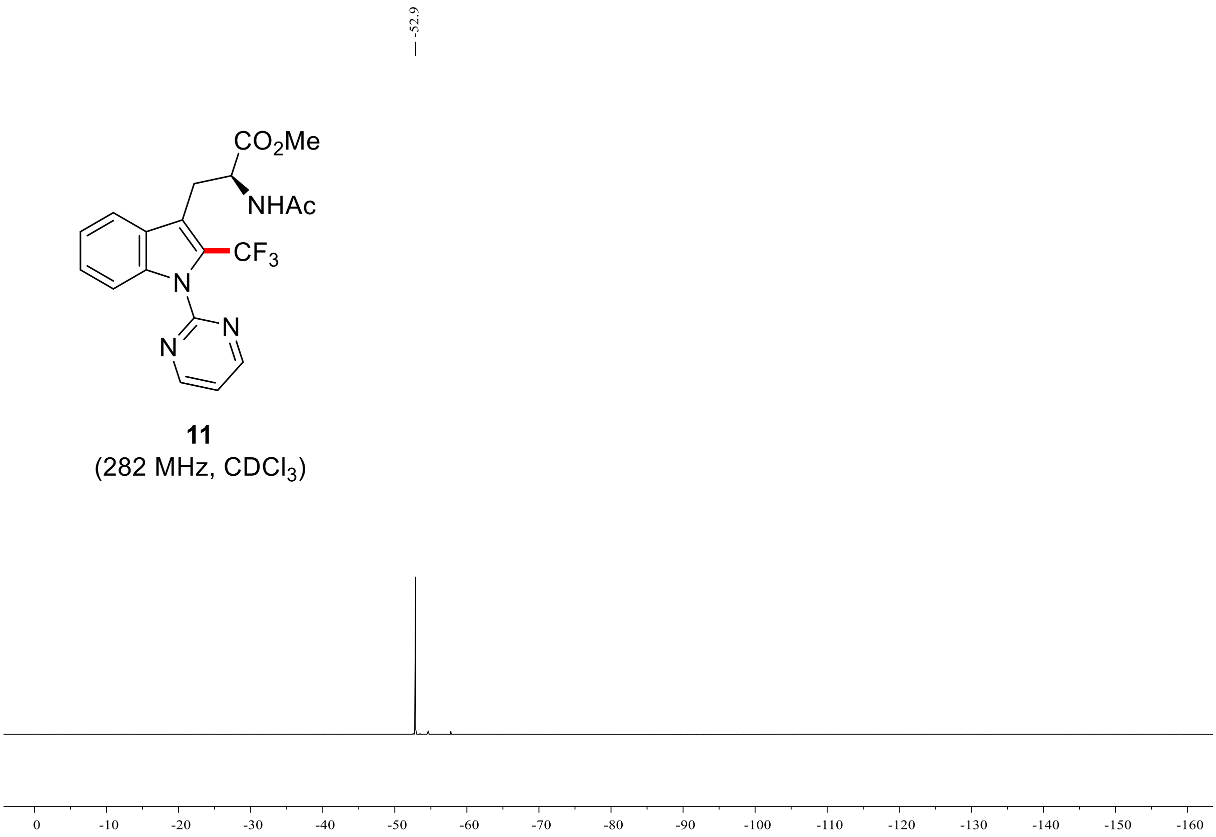
CO2Me
NHAc
CF3
N
N
N
11
(282 MHz, CDCl3)
-52.9
0 -10 -20 -30 -40 -50 -60 -70 -80 -90 -100 -110 -120 -130 -140 -150 -160

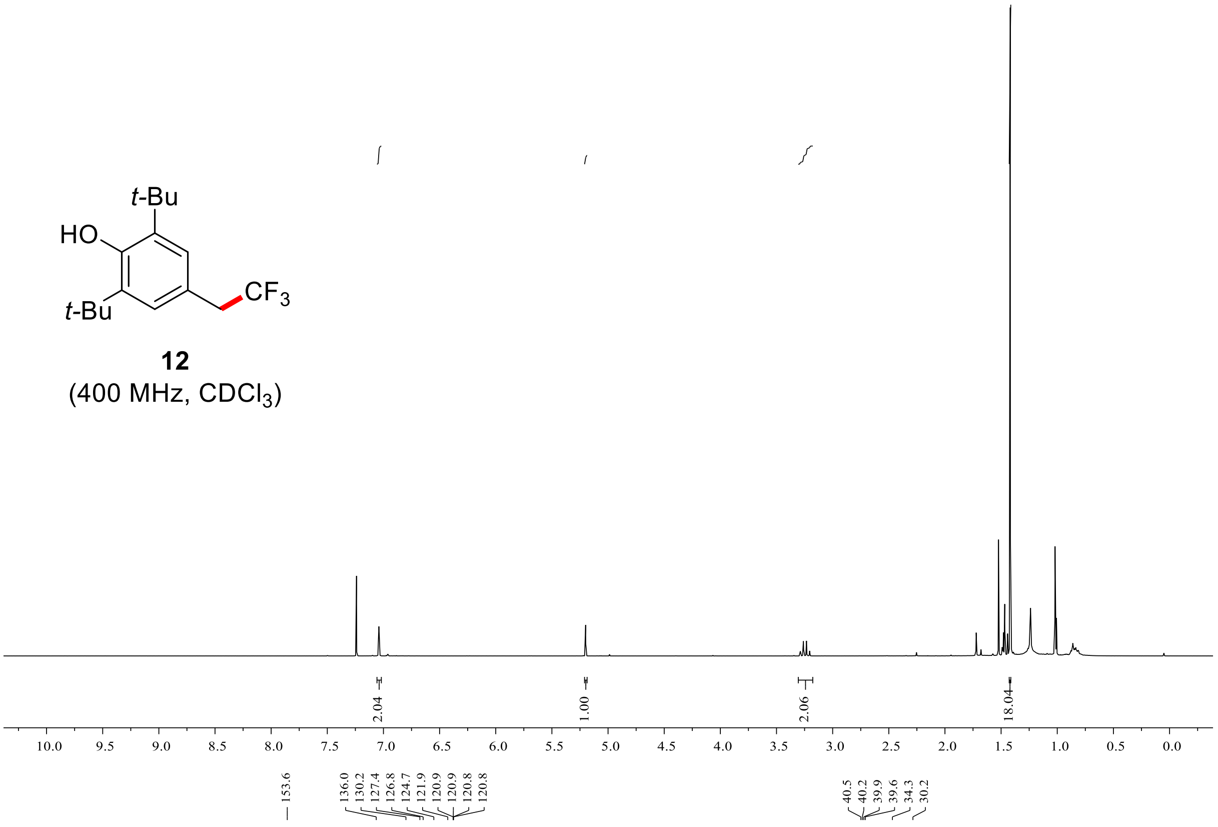
t-Bu
HO
t-Bu
CF3
12
(400 MHz, CDCl3)
2.04
1.00
2.06
18.04
10.0 9.5 9.0 8.5 8.0 7.5 7.0 6.5 6.0 5.5 5.0 4.5 4.0 3.5 3.0 2.5 2.0 1.5 1.0 0.5 0.0
153.6
136.0 130.2 127.4 126.8 124.7 121.9 120.9 120.9 120.8 120.8
40.5 40.2 39.9 39.6 34.3 30.2

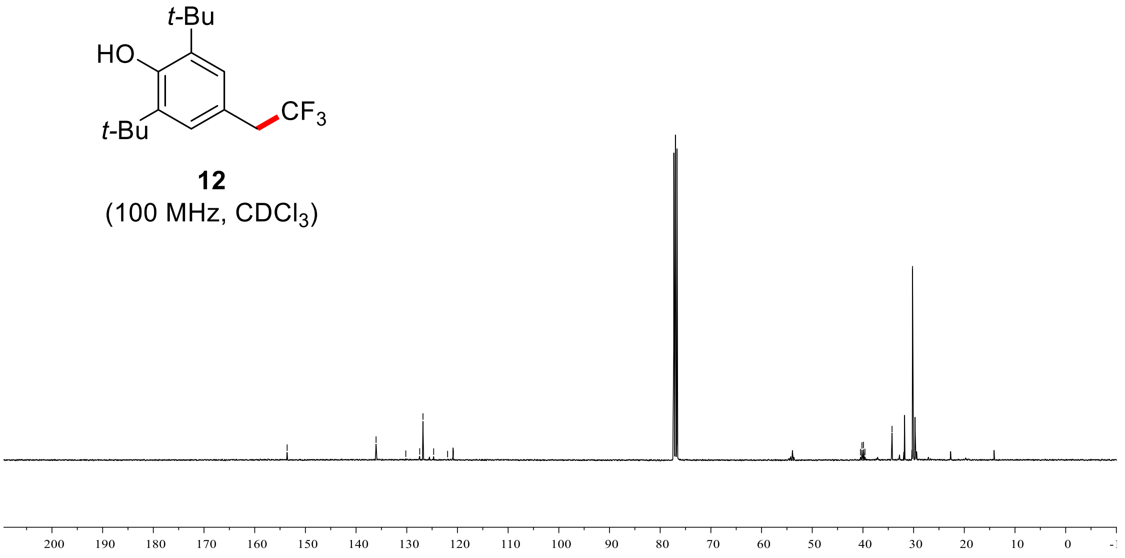
t-Bu
HO
t-Bu
CF3
12
(100 MHz, CDCl3)
200 190 180 170 160 150 140 130 120 110 100 90 80 70 60 50 40 30 20 10 0

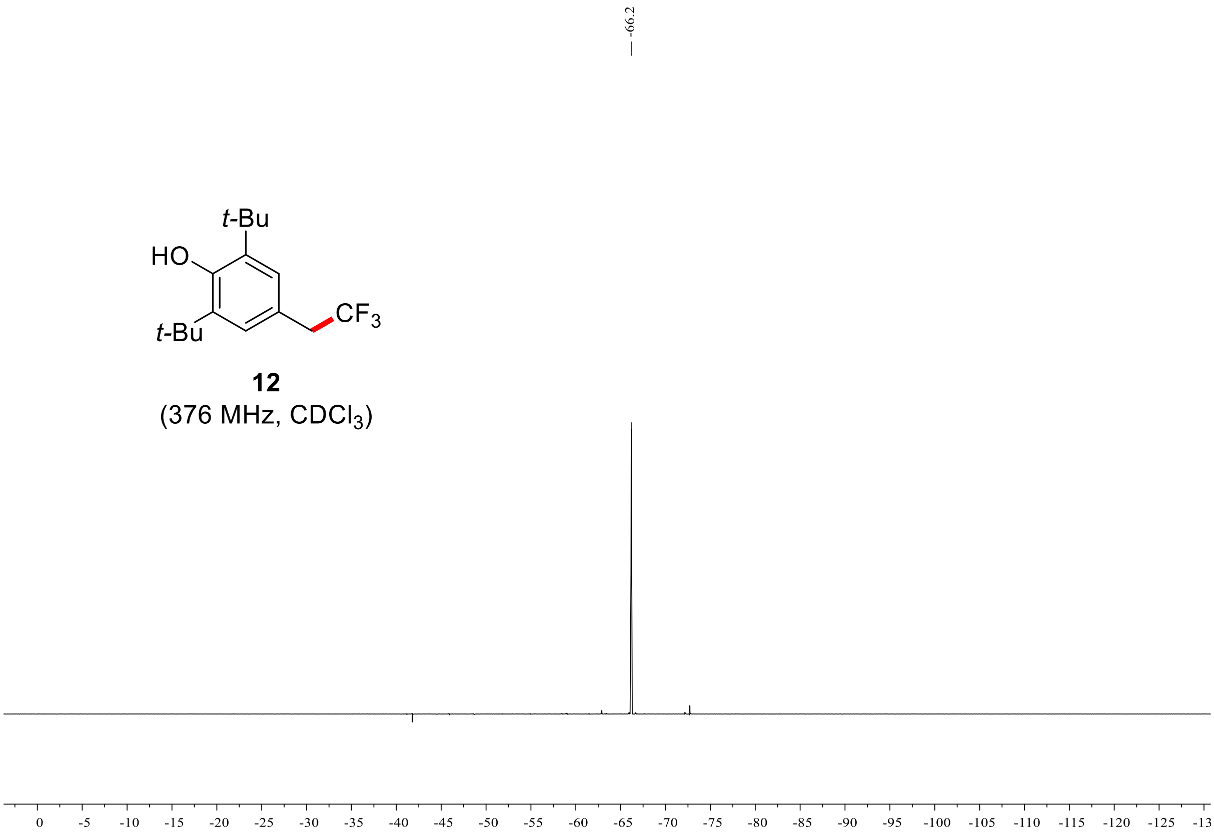
-66.2
t-Bu
HO
t-Bu
CF3
12
(376 MHz, CDCl3)
0 -5 -10 -15 -20 -25 -30 -35 -40 -45 -50 -55 -60 -65 -70 -75 -80 -85 -90 -95 -100 -105 -110 -115 -120 -125 -13

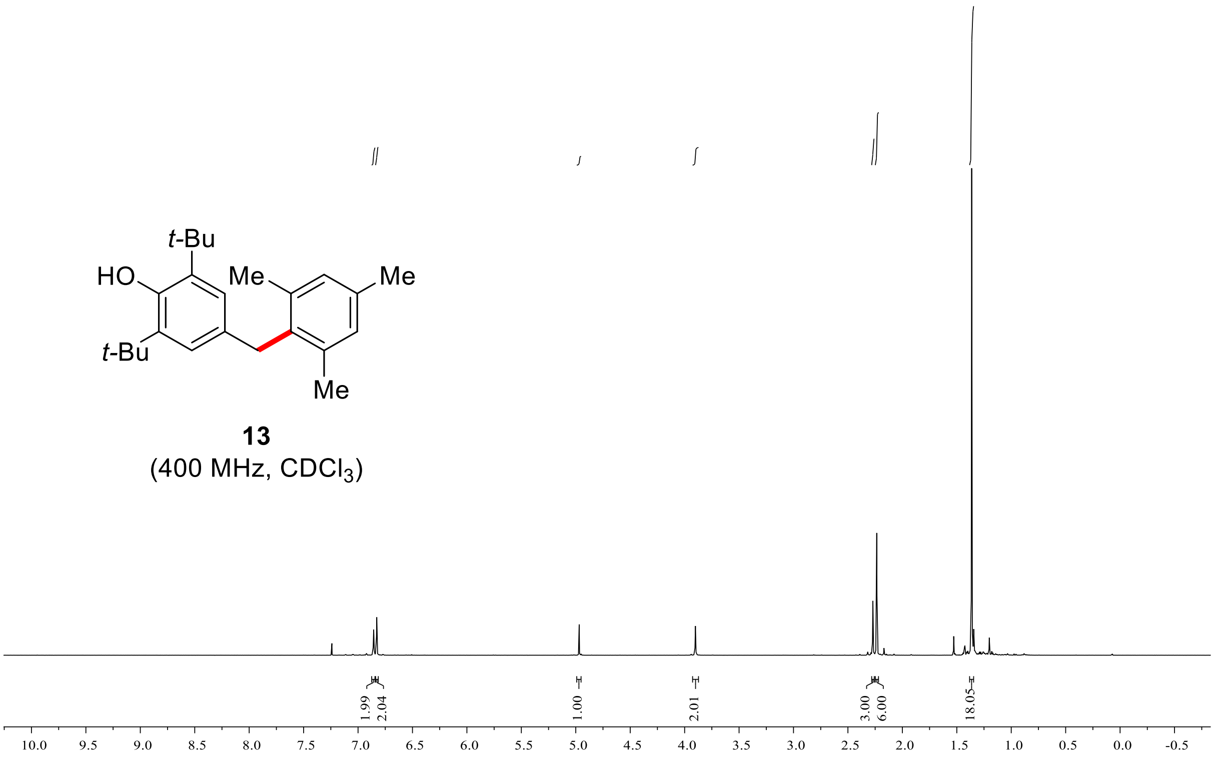
t-Bu
HO
Me
Me
t-Bu
Me
13
(400 MHz, CDCl3)
1.99
2.04
1.00
2.01
3.00
6.00
18.05
10.0 9.5 9.0 8.5 8.0 7.5 7.0 6.5 6.0 5.5 5.0 4.5 4.0 3.5 3.0 2.5 2.0 1.5 1.0 0.5 0.0 -0.5

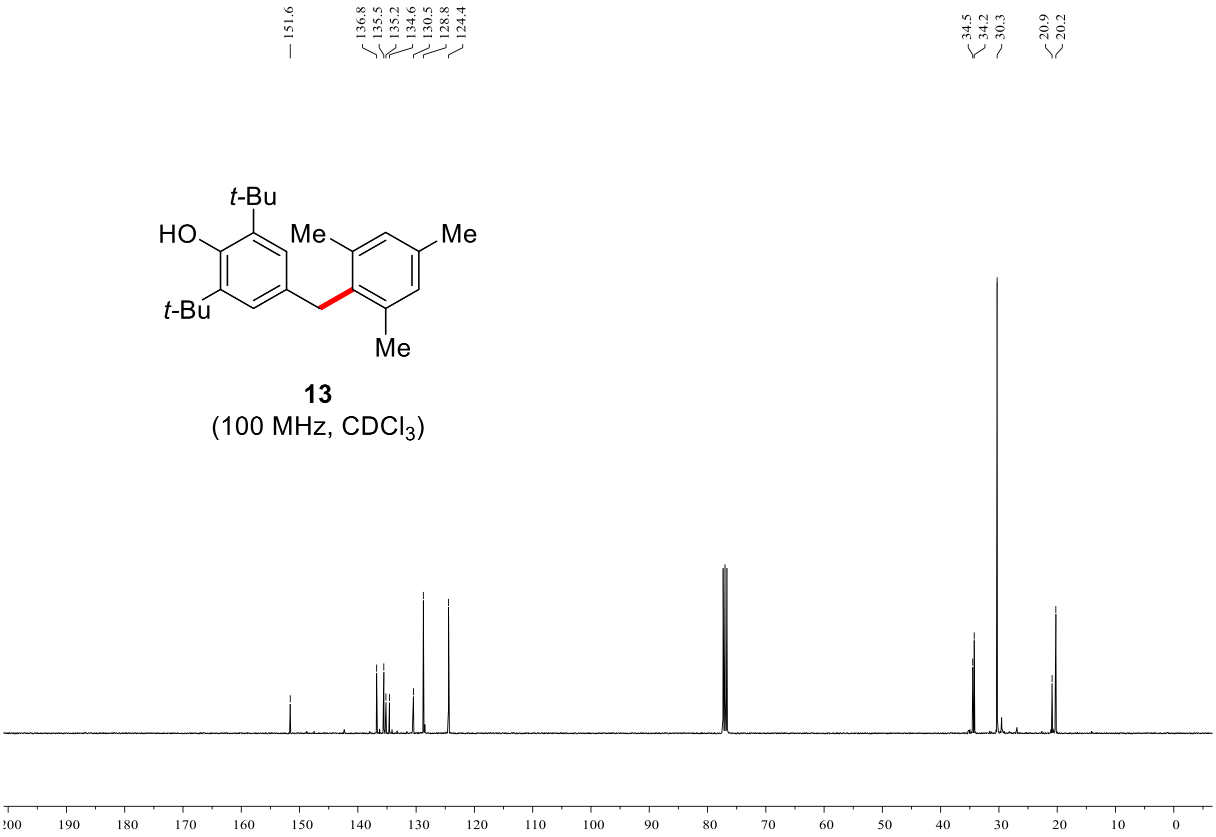

13

(100 MHz, $CDCl_3$)
